# vEMRec: High‐Resolution Volume Electron Microscopy Reconstruction Based on Structure‐Preserving and High‐Fidelity 3D Alignment

**DOI:** 10.1002/advs.202519098

**Published:** 2026-02-20

**Authors:** Zhenbang Zhang, Hongjia Li, Zhongjun Yang, Zhiqiang Xu, Duanchen Sun, Xin Gao, Fa Zhang, Renmin Han

**Affiliations:** ^1^ School of Medical Technology Beijing Institute of Technology Beijing China; ^2^ Research Center for Mathematics and Interdisciplinary Sciences Shandong University Qingdao China; ^3^ College of Medical Information and Engineering Ningxia Medical University Yinchuan China; ^4^ Qilu Hospital Cheeloo College of Medicine Shandong University Qingdao China; ^5^ Department of Machine Learning Mohamed bin Zayed University of Artificial Intelligence, Masdar City Abu Dhabi UAE; ^6^ School of Mathematics Shandong University Jinan China; ^7^ Computer Science Program Computer, Electrical and Mathematical Sciences and Engineering(CEMSE) Division King Abdullah University of Science and Technology (KAUST) Thuwal Saudi Arabia

**Keywords:** deep learning, series section electron microscopy, volume electron microscopy

## Abstract

Three‐dimensional (3D) alignment is a key step in volume electron microscopy (vEM), aimed at addressing misalignment during data acquisition, thereby recovering the correct biological structures. However, automated 3D alignment has long been challenged by the dilemma between eliminating nonlinear distortions and capturing natural morphological variations inherent to biological specimens. Here, we present vEMRec, a paradigm‐shifting, fully automated algorithm for vEM 3D alignment. vEMRec redefines the 3D alignment problem by decoupling it into high‐frequency and low‐frequency subproblems. In this framework, precision rigid alignment is applied to correct rigid distortions, while a Gaussian filter‐driven elastic registration algorithm addresses nonlinear distortions, all the while faithfully preserving biologically plausible deformations. Extensive experiments demonstrate that vEMRec achieves a paradigm shift in 3D alignment. Serving as a critical preprocessing step, vEMRec enhances performance in downstream isotropic reconstruction and 3D segmentation tasks by improving axial continuity in anisotropic data while preserving the structural integrity of ultrastructural details. Moreover, vEMRec accomplishes this through efficient computation, enabling TB‐scale specimen analysis with biologically relevant throughput. vEMRec successfully optimized six representative large‐scale real‐world datasets, demonstrating its applicability, accuracy, and robustness for large‐scale data processing.

## Graphical abstract

vEMRec is a frequency‐adaptive computational framework for three‐dimensional alignment in volume electron microscopy. It integrates feature‐based rigid alignment with Gaussian filter‐guided elastic registration to correct rigid misalignments and nonlinear distortions while preserving structural fidelity. Designed from a reconstruction‐centric perspective, vEMRec enables accurate, scalable, and high‐throughput volumetric reconstruction through an efficient and fully automated processing architecture.

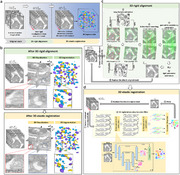

## 1 Introduction

Volume electron microscopy (vEM) is a powerful technique for high‐resolution 3D visualization of biological tissues and cells, revealing complex nanoscale cellular structures [1, 2] and generating detailed models of macromolecular complexes [3]. It has extensive applications in life sciences [4, 5], medicine [6, 7, 8], and clinical diagnostics [9, 10, 11, 12], contributing to significant breakthroughs. For example, in connectomics research, vEM is crucial for mapping neuronal connections and reconstructing the 3D ultrastructure of cells and tissues [13, 14, 15, 16]. Recently, MICrONS project [17, 18, 19, 20] utilizes vEM as the fundamental method for neural ciruit reconstruction and connectomic analysis. The vEM workflow typically begins with continuous serial section tomography, where biological samples are carefully sliced along the z‐axis. After slicing, the samples are stained and digitally imaged. The generated 2D digital images undergo 2D stitching, 3D alignment, and 3D segmentation to reconstruct the complete biological structure [21]. Among these, 3D alignment is a critical step in this workflow, as it corrects for rigid misalignments and nonlinear distortions introduced during sample preparation, ultimately determining the accuracy and quality of the reconstructed 3D structure.

However, with the continuous advancement of vEM slice preparation techniques, state‐of‐the‐art equipment can now generate terabyte‐scale image data on a daily basis [3]. The exponential growth of data imposes higher demands on the efficiency and scalability of 3D alignment algorithms. Moreover, error accumulation in long image sequences further increases the difficulty of achieving robust vEM alignment [21]. One of the core challenges in precise 3D alignment lies in effectively distinguishing between natural cellular morphological changes and nonlinear deformations introduced during sample preparation. The former refers to physiological variations in cell morphology, while the latter represents distortions caused by sample processing [22]. The ambiguous boundary between these two types of deformations significantly complicates the accurate restoration of the specimen's true 3D structure, making precise alignment a critical and highly challenging task.

Traditional methods for 3D alignment primarily rely on optimizing energy functions. TrakEM2 [23, 24] is a widely used software package, performing 3D alignment by iteratively optimizing a spring‐connected particle system modeled as a triangular spring mesh. In recent years, some deep learning‐based methods have been proposed to address the challenge of 3D alignment in vEM. Xin et al. [25] employed UTR descriptors to calculate slice similarity loss and used an optical flow network to estimate the deformation field, introducing a structured regression approach to minimize error accumulation; however, their method struggles to generalize to long sequences. Popovych et al. [26] introduced SEAMLeSS, a trillion‐scale computing pipeline utilizing a self‐supervised convolutional network for metric learning. Their method iteratively refines initial alignment and incorporates vector voting to enhance robustness, dividing images into overlapping blocks for distributed computing while achieving global alignment through combined attenuation transformations. Although these deep learning‐based methods [25, 26] show improvements in accuracy and speed compared to traditional methods, they often overlook the natural deformations between slices. If the focus is solely on pixel‐level similarity between adjacent slices, it may inadvertently eliminate the natural deformations between them, thereby destroying the axial structure of biological tissues.

In this work, we introduce vEMRec, a paradigm‐shifting computational framework that fundamentally redefines 3D alignment through a frequency‐adaptive processing architecture. vEMRec synergistically combines an efficient, fully automated 3D alignment algorithm with dedicated software, enabling scalable, high‐throughput analysis. vEMRec focuses on 3D alignment, it is designed from a reconstruction‐centric perspective, as accurate and structure‐preserving alignment constitutes the fundamental prerequisite for reliable volumetric reconstruction in volume electron microscopy. It synergistically couples feature‐based rigid alignment with Gaussian filter–guided elastic registration, enabling the simultaneous correction of rigid misalignments and nonlinear distortions while maintaining biologically realistic structural fidelity. We further designed a novel metric to quantitatively assess alignment quality, addressing the challenge of evaluation in the absence of ground truth. We validated vEMRec on six synthetic datasets representing diverse cell types and six widely used real‐world datasets. Across these benchmarks, vEMRec consistently achieved a 20%–30% improvement in alignment accuracy, along with significantly enhanced computational efficiency, underscoring its potential for large‐scale applications. vEMRec exhibits robust performance across diverse imaging conditions, effectively coping with high noise, alignment errors, and complex deformations. Furthermore, we demonstrate that vEMRec, as a critical upstream preprocessing step, substantially enhances the performance of downstream tasks, including isotropic reconstruction fidelity and 3D segmentation accuracy. These synergistic improvements establish vEMRec as a powerful and indispensable tool for volume electron microscopy, providing a reliable foundation for high‐throughput biological imaging with both computational efficiency and alignment fidelity at scale.

## 2 Results

### 2.1 Overview of the vEMRec Procedure

**FIGURE 1:**
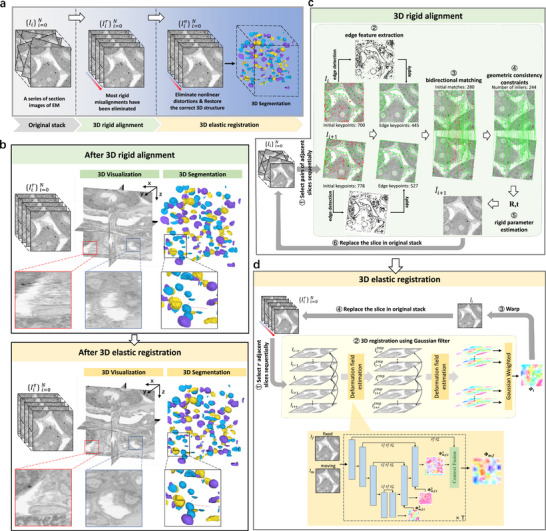
Overview of the vEMRec processes. (a) vEMRec consists of two core modules: 3D rigid alignment and 3D elastic registration. (b) Visualization of image stacks and corresponding 3D segmentation results at different stages (using mouse liver images (FIB‐SEM) [27] as example data). After the 3D rigid alignment step, the image stack ${\left\{I_{i}^{r}\right\}}_{i=0}^{N}$ provides a preliminarily corrected 3D segmentation structure, though minor nonlinear distortions persist. These distortions are corrected during the 3D elastic registration process, resulting in a smoother and more continuous 3D structure, as seen in ${\left\{I_{i}^{e}\right\}}_{i=0}^{N}$. (c) 3D rigid alignment pipeline. For each adjacent slice pair $\left\{I_{i},I_{i+1}\right\}$, edge feature points are extracted and then filtered by an edge mask, followed by bidirectional nearest‐neighbor matching. Geometric consistency constraints are then applied to establish robust correspondences, from which the rigid transformation parameters are computed, yielding the aligned stack ${\left\{I_{i}^{r}\right\}}_{i=0}^{N}$. The alignment process for a slice pair is illustrated, showing edge feature extraction, matching, outlier filtering, and parameter estimation, with red points representing non‐edge features, green points representing edge features, and green and red lines indicating correct and incorrect correspondences, respectively. (d) 3D elastic registration pipeline. For each slice $I_{i}^{r}$ and its neighboring slices $N(I_{i}^{r})$, the dense displacement field is estimated using a cascaded pyramid optical flow network. The deformation fields are then refined through a Gaussian filtering technique. This process is applied sequentially across the image stack, producing the fully elastically registered image stack ${\left\{I_{i}^{e}\right\}}_{i=0}^{N}$.

**FIGURE 2:**
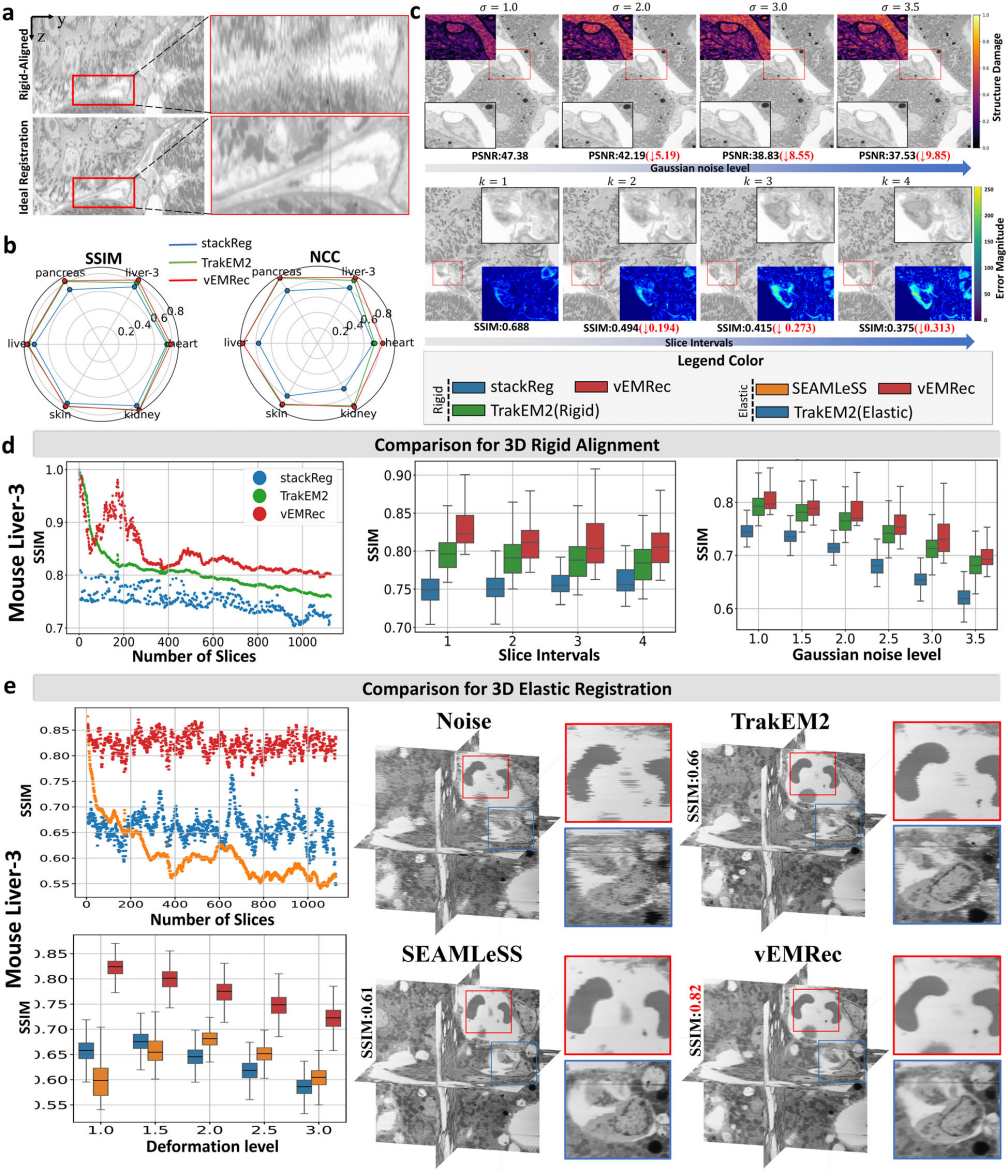
vEMRec excels in eliminating rigid misalignment and nonlinear distortion, enabling precise 3D structural alignment. (a) Despite rigid alignment, the resulting image stacks still exhibit abrupt and subtle nonlinear misalignments. Ideal registration restores smooth and continuous axial structures. (b) Quantitative comparison of the average alignment accuracy, measured by SSIM (Structural Similarity Index Measure) and NCC (Normalized Cross‐Correlation), for rigid alignment across six synthetic datasets. Each slice was cropped to a resolution of 1024×1024 pixels. The translation parameters $\left(t_{x},t_{y}\right)$ were randomly sampled from the range [0,100], and the rotation angles θ were randomly selected from the interval $\left[-35^{\circ },35^{\circ }\right]$. For further details, please refer to Section S1 (c) Visualization of slice distortions under different Gaussian noise levels and slice intervals. The heatmaps illustrate the intensity of structural damage (1−SSIM) and the corresponding differential maps highlighting inter‐slice variations. (d) Rigid alignment performance on the Mouse Liver‐3 dataset. Left: SSIM degradation due to accumulated alignment error as slice count increases. Middle: SSIM variation under different levels of Gaussian noise. Right: SSIM changes across increasing deformation severity (larger slice intervals). (e) Elastic registration performance on the Mouse Liver‐3 dataset. Left: Trends of average registration accuracy (SSIM(GT)) with increasing slice numbers, and SSIM(GT) under varying deformation levels (α). Right: 3D visualizations of registration results showing improved structural continuity(deformation levels α=1.0).

Figure 1a illustrates the overall vEMRec pipeline, which comprises two main stages: a rigid alignment module (Figure 1c) and a core elastic registration module (Figure 1d). The goal of rigid alignment is to provide an initial global registration of the image stack, serving as a preprocessing step. In contrast, the elastic registration module constitutes our core algorithmic contribution, enabling the correction of complex nonlinear distortions and the recovery of the true 3D structure of the specimen. Specifically, during the rigid alignment stage, each adjacent slice pair $\left\{I_{i},I_{i+1}\right\}$ is first processed by extracting robust feature points using SuperPoint [28]. These features are refined using an edge mask and matched through bidirectional nearest‐neighbor search. The Locality Preserving Matching (LPM) algorithm [29] is then applied to further filter the correspondences, improving the robustness of alignment. Rigid transformation parameters are estimated and propagated sequentially across the image stack, producing a rigidly aligned sequence ${\left\{I_{i}^{r}\right\}}_{i=0}^{N}$ (see top row of Figure 1b). To eliminate remaining nonlinear distortions that compromise continuity along the z‐axis, a 3D elastic registration is performed. As shown in Figure 2a, fine, abrupt nonlinear distortions correspond to high‐frequency information, while smooth, continuous natural deformations reflect low‐frequency information. This step decomposes the registration problem into high‐ and low‐frequency components, allowing us to separate imaging artifacts from biologically plausible deformations. A cascaded pyramid optical flow network is used to estimate voxel‐wise deformation fields, which are subsequently refined and integrated using an extended 3D Gaussian smoothing technique. The final aligned image stack is then obtained by warping the rigidly aligned volumes with the integrated deformation field, effectively restoring the underlying anatomical structure (see bottom row of Figure 1b). This enables our approach to remove nonlinear distortions while preserving natural deformations between adjacent slices, thereby accurately restoring the 3D structure of the image stack. The reconstructed 3D image stack can be directly used in downstream applications such as isotropic volume reconstruction and 3D segmentation. Further details about the algorithm and network architecture are provided in Section 4.

### 2.2 Comprehensive Validation of the Rigid Alignment and Elastic Registration Modules in vEMRec

To rigorously assess the performance of vEMRec in high‐precision 3D alignment, we systematically validated its rigid alignment and elastic registration modules across six synthetic datasets representing diverse tissue types (Section S1). In the rigid alignment stage, the goal is to remove global translational and rotational misalignments, providing accurate spatial initialization for downstream registration. The vEMRec rigid alignment module outperformed two widely used 3D alignment tools available on the FIJI platform, a widely used open‐source application for biological image analysis, namely StackReg and TrakEM2 [24] (Figure 2b). Subsequently, vEMRec applied elastic registration to correct local nonlinear deformations, enabling high‐fidelity alignment of tissue morphology (Figure 2b). We compared vEMRec against the elastic registration module in TrakEM2 [24] and the recent deep learning‐based method SEAMLeSS [26]. vEMRec consistently achieved the highest registration fidelity. Across these benchmarks, vEMRec consistently achieved a 20%–30% improvement in alignment accuracy (Figure 2e, left), along with significantly enhanced computational efficiency (Figure 4a). Evaluations were based on standard metrics, including Normalized Cross‐Correlation (NCC), Structural Similarity Index Measure (SSIM), and Mutual Information (MI).

**FIGURE 3:**
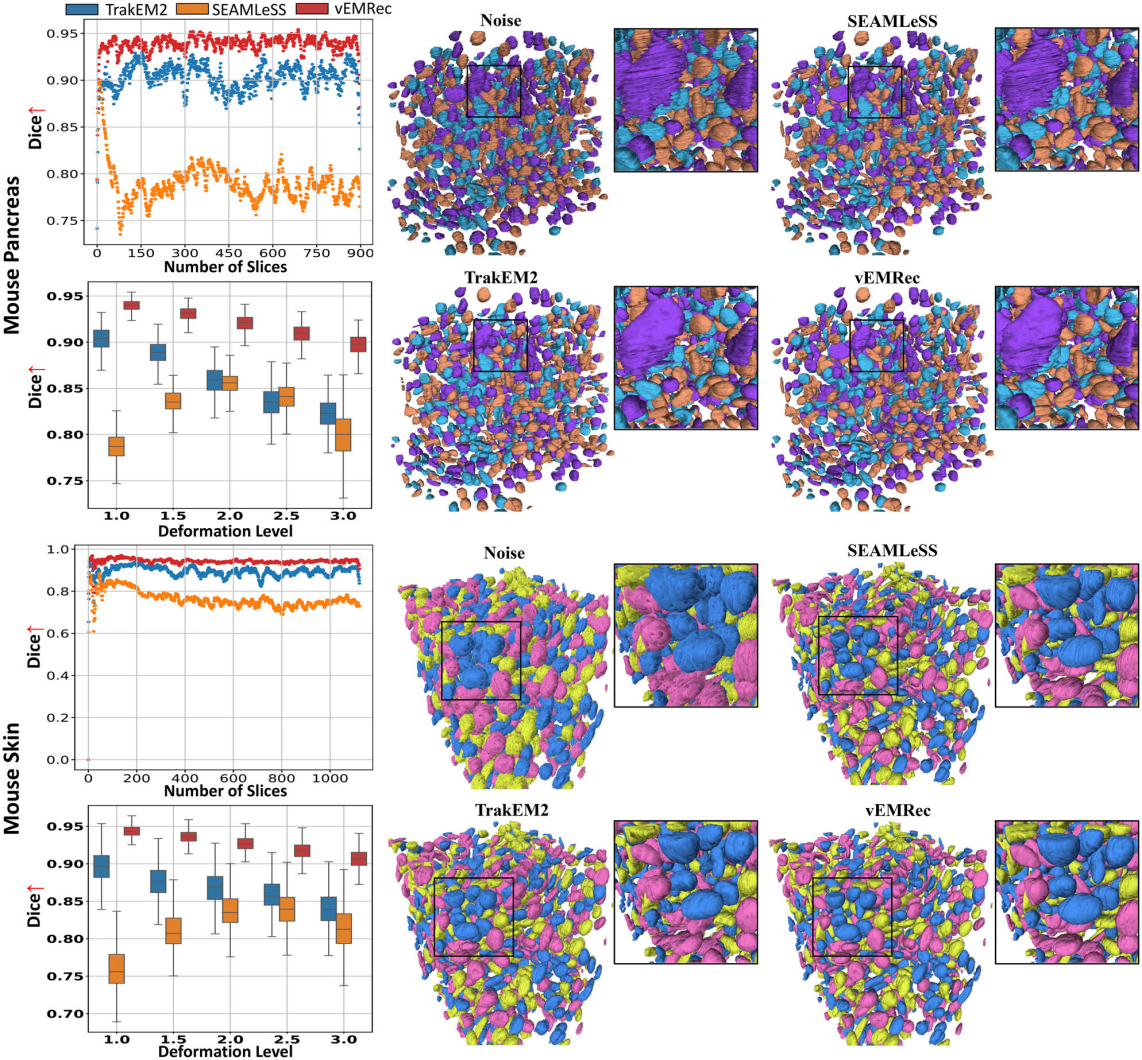
Our method demonstrates superior performance in the downstream task of 3D segmentation. Left: Trends in 3D segmentation accuracy (Dice) for various methods as the number of slices increases, and the Dice of different methods under varying levels of nonlinear distortion. (Additional experimental results are provided in Figures S15 and S16) Right: 3D visualizations of segmentation results.

**FIGURE 4:**
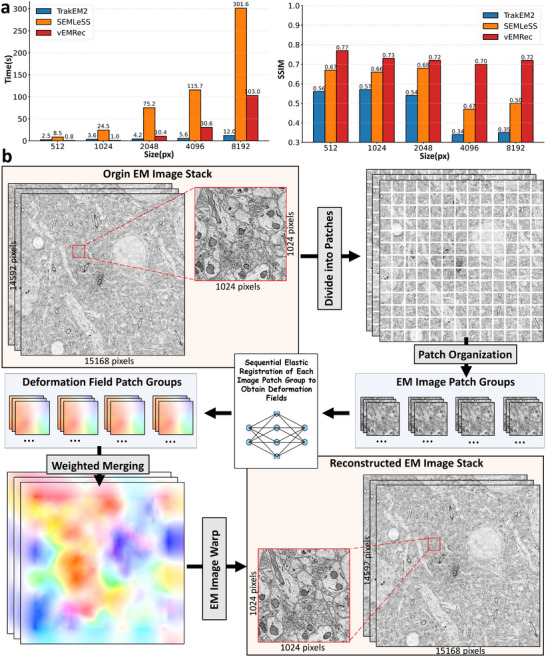
(a) Processing time (s) and SSIM of a single slice processed by vEMRec under different slice sizes. This benchmark was measured on a single high‐performance workstation equipped with an NVIDIA A100 GPU (40 GB memory) and two AMD EPYC 7T83 CPUs (96 physical cores, 192 threads) with 512 GB of system RAM. The measurement corresponds to a single‐batch inference setting, where one 1024×1024 slice was processed sequentially per batch on the GPU, resulting in an average processing time of about 1 s per slice. (b) Pipeline of our alignment strategy adapted for high‐resolution EM data. The example images are taken from the mouse cortical dataset (ATUM‐SEM) [31].

#### 2.2.1 Addressing Error Accumulation in Long Image Sequences

In real‐world applications, serial‐section EM (ssEM) datasets often comprise hundreds to thousands of slices, where even minor alignment errors can accumulate, leading to significant drift and compromising 3D alignment. To investigate this issue, we conducted experiments on a mouse liver dataset containing 1127 slices (The results of other datasets can be found in Figures S5 and S6), focusing on error accumulation in rigid alignment. We observed that the alignment accuracy (measured by SSIM) of all tested rigid alignment algorithms decreased with increasing slice count due to accumulated errors. Notably, vEMRec consistently outperformed competing methods across all stages (Figure 2d, left). Although vEMRec's rigid alignment effectively suppresses error propagation, residual misalignments due to natural tissue deformation remain. These were further corrected by vEMRec's elastic registration module, which exhibited excellent stability even in long sequences (Figure 2e left; The results of other datasets can be found in Figures S11 and S12). Across datasets exceeding 1000 slices, vEMRec maintained the highest overall registration accuracy with no noticeable degradation, significantly outperforming both TrakEM2 and SEAMLeSS, achieved a 20%–30% improvement in SSIM.

#### 2.2.2 Handling High‐level Deformations and Noise in Image Sequence

Electron microscopy images often suffer from elastic deformations stemming from limited axial resolution, mechanical sectioning artifacts, and varying noise levels, posing major challenges to precise 3D alignment. To further evaluate robustness, we conducted experiments under two challenging conditions: severe noise and complex elastic deformation. Figure 2c illustrates that as noise levels increase, biological structures in the images become progressively blurred and less distinguishable. In addition, Figure S3 provides a complementary visualization using Laplacian‐based high‐frequency residual maps and the radial PSD comparison, which explicitly reveals the spatial amplification of high‐frequency noise and clarify how increasing noise degrades structural fidelity beyond what is readily observable in the raw images. As shown in Figure 2d right, vEMRec consistently achieved higher average alignment accuracy (SSIM) than other methods at all noise levels (The results of other datasets can be found in Figures S7 and S8). To simulate stronger inter‐slice deformations, we increased slice intervals in the image stacks where larger intervals correspond to greater tissue variability between adjacent slices (blue difference maps in Figure 2c). vEMRec again achieved the highest rigid alignment accuracy under all interval settings (Figure, 2d middle). Further evaluation of the elastic registration module across deformation levels (Figure S4) showed vEMRec consistently provided the highest registration accuracy (SSIM) (Figure 2e left; Figures S13 and S14). Notably, vEMRec successfully reconstructed precise 3D structures and preserved z‐axial continuity and smoothness(Figure 2d, right). In contrast, SEAMLeSS exhibited structural truncations, producing stair‐step artifacts, while TrakEM2 restored overall structure but retained minor distortions at cell edges (Figures S17 and S18).

#### 2.2.3 Registration Enhances Downstream 3D Segmentation Accuracy

vEM 3D segmentation is a crucial downstream task of 3D alignment, with broad applications in tissue recognition [30] and cell segmentation [27]. The quality of alignment directly impacts segmentation performance by mitigating structural deformations, scale variations, and inter‐slice misalignments, especially across heterogeneous imaging modalities. To evaluate this impact, we compared the alignment performance of vEMRec, TrakEM2, and SEAMLeSS on downstream 3D segmentation using the Dice Similarity Coefficient (Dice score). Detailed experimental configurations are provided in Sections S3 and S4. We analyzed the average Dice scores across varying slice counts and deformation levels. vEMRec consistently maintained the highest Dice scores across entire stacks, even as the sequence length approached 1000 slices, demonstrating strong robustness in long‐sequence alignment (Figure 3, left). Furthermore, despite the presence of complex nonlinear deformations, vEMRec sustained high segmentation accuracy, achieving the top Dice scores at all tested deformation levels (Figure 3, left). Visual inspection of the segmentation outputs (Figure 3, right) further highlights vEMRec's superiority. It captures subtle anatomical details and fine structures within liver tissue more clearly than other approaches, yielding more precise and continuous segmentation results. Additional examples are provided in Figures S19 and S20. These results demonstrate that accurate structural alignment by vEMRec provides a robust foundation for high‐quality 3D segmentation and facilitates deeper analysis of complex biological structures.

### 2.3 Validation of vEMRec on Large‐Scale Real‐World Data

As large‐scale electron microscopy (EM) image datasets become increasingly common, the demand for efficient, high‐throughput 3D alignment methods has intensified [32]. Compared with TrakEM2 and SEAMLess, vEMRec demonstrates superior alignment accuracy and competitive computational efficiency on a mouse liver dataset [32], with image sizes ranging from 512×512 to 8192×8192 pixels (Figure 4a). For a fair comparison with the CPU‐based TrakEM2, we used a batch size of 1 for the GPU‐based SEAMLess and vEMRec. To further ensure the flexibility and scalability of vEMRec for ultra–high‐resolution EM datasets exceeding 10000×10000 pixels, we designed an efficient hierarchical registration pipeline (Figure 4b). For ultra‐large EM images (e.g., Figure 4, resolution 14592×15168 pixels), we first divided each image into multiple overlapping patches (each of size 1024×1024 pixels, which was demonstrated to get the best efficiency), which are then grouped based on their spatial locations in the XY plane. Within each patch group, a local deformation field is computed. These local deformation fields are then fused using a spatially weighted strategy to generate a global high‐resolution deformation field, which is applied to correct and align the original image stack. This patch‐wise strategy enables our algorithm to scale efficiently while maintaining high registration accuracy for massive datasets.

To demonstrate the practicality of the proposed parallel strategy for large‐scale data processing, we further conducted experiments on multi‐batch processing using a single GPU. As summarized in Table 1, taking the 1024×1024 case as an example, increasing the batch size from 1 to 4 resulted in a moderate increase in GPU memory usage from approximately 3 to 7 GB, while reducing the average processing time per slice from 0.98 to 0.38 s. When the batch size was further increased to 8, the memory usage rose to around 15 GB, and the per‐slice processing time decreased to 0.21 s. At a batch size of 16, GPU memory consumption reached about 37 GB, with the per‐slice processing time further reduced to only 0.16 s. These experiments were performed on a single NVIDIA A100 GPU (40 GB memory). The results indicate that the proposed parallel strategy effectively improves computational efficiency with increasing batch size while maintaining reasonable GPU memory requirements. Furthermore, this design can be readily extended to multi‐GPU environments, enabling near‐linear increases in processing throughput as more GPUs are utilized. In practice, the overall scalability is mainly constrained by data I/O bandwidth and storage concurrency, but the approach nonetheless provides a practical and efficient pathway for aligning very large‐scale vEM datasets.

**TABLE 1:** Average processing time (s) per slice and estimated GPU memory usage under different batch sizes.

Batch size	512×512	1024×1024	2048×2048	4096×4096	8192×8192	GPU memory
1	0.78	0.98	10.42	30.56	103.01	∼3 GB
4	0.22	0.38	3.80	10.52	48.21	∼7 GB
8	0.13	0.21	1.61	6.83	25.55	∼15 GB
16	0.09	0.16	1.12	4.38	16.10	∼37 GB

To validate the effectiveness and generalizability of our alignment framework, we evaluated vEMRec on five publicly available large‐scale volumetric EM datasets acquired using different imaging modalities, including the FAFB (Full Adult Fly Brain, ssTEM/TEMCA) [13], the *Caenorhabditis elegans* cell dataset (TEM) [33], the mouse cortical dataset (ATUM‐SEM) [31], the female fruit fly brain neural dataset (TEM) [34], and the human term placental villi dataset (SBF‐SEM) [35]. The image sizes range from 3000×3000 to 21504×26624 pixels (see Section 4.1 for dataset details). All alignments were performed on raw image stacks without any prior alignment to demonstrate that *vEMRec* can robustly handle raw EM datasets and produce improved 3D alignment with enhanced structural clarity and visual quality.

#### 2.3.1 Multi‐Scale Difference Filtering Evaluation Strategy for Real Datasets

To evaluate registration performance on real datasets, a reliable ground‐truth–free metric is required. Existing approaches [24, 36, 37] are labor‐intensive, require substantial domain expertise, and are difficult to automate. To address these limitations, we propose a multi‐scale difference filtering evaluation strategy, motivated by the observation that poor registration often introduces random, independent noise in the difference maps between adjacent slices, whereas successful registration preserves biologically meaningful texture patterns corresponding to natural structural variations. Our strategy evaluates registration quality from two complementary aspects: noise suppression and structure preservation.

**FIGURE 5:**
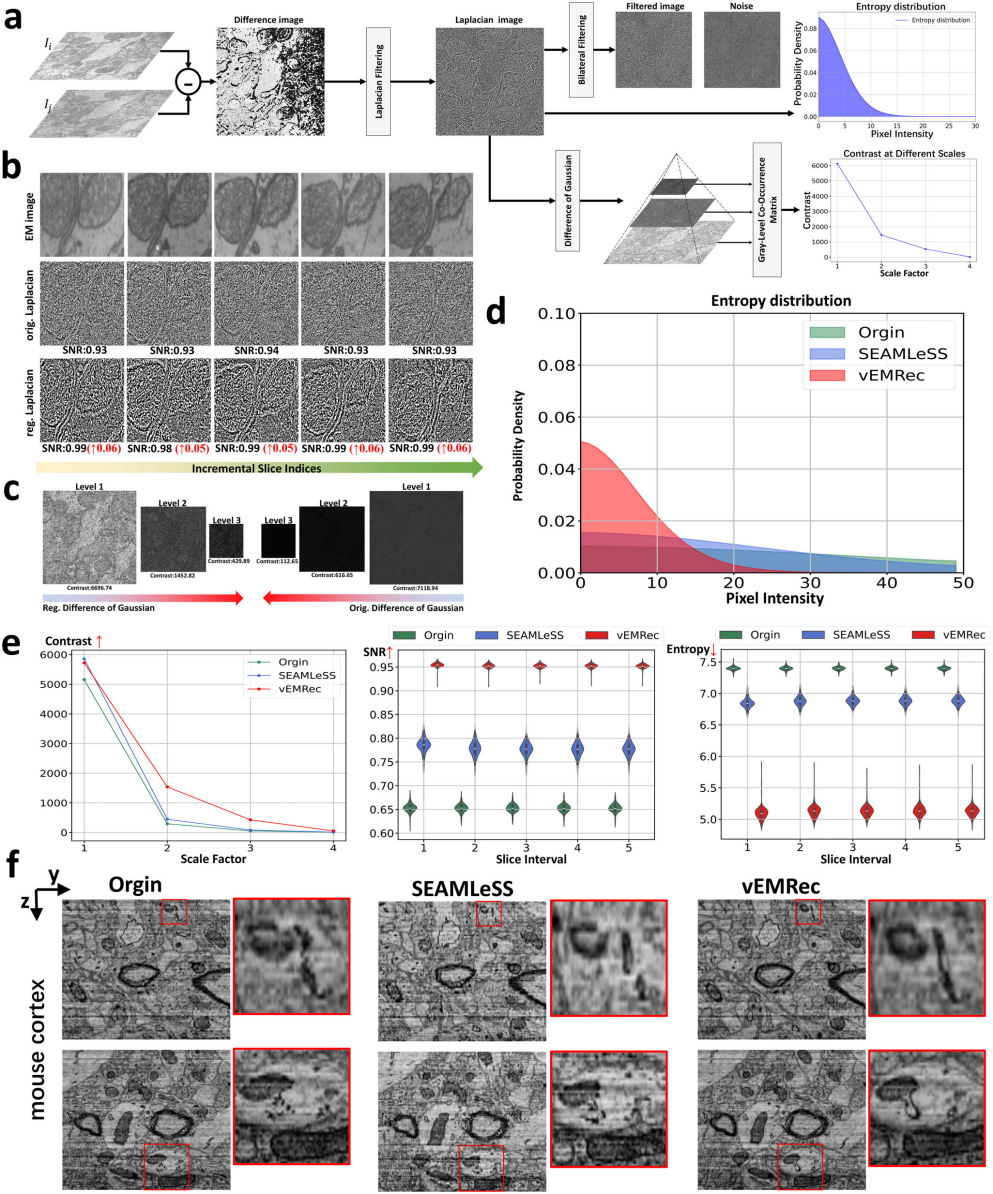
Our alignment strategy performs exceptionally well on real datasets. (a) The pipeline of our proposed multi‐scale differential filtering evaluation strategy. We first compute the differences between adjacent slices, then apply Laplacian filtering to the difference images and construct a Difference of Gaussian (DoG) pyramid. Finally, we perform multi‐dimensional evaluations using signal‐to‐noise ratio (SNR), image entropy distribution, and multi‐scale contrast analysis. (b) Laplacian‐filtered images of the original data and our registration results, showing reduced noise and improved z‐axial continuity in the registered results. (c) Difference of Gaussian (DoG) comparisons between the original and registered data, where the registered data preserves more texture information across multiple scales. (d) Entropy distribution histograms of the original and registered image data. (e) Comparison of multi‐scale contrast, signal‐to‐noise ratio (SNR), and image entropy metrics between the original and registered data. (f) Side view comparisons between the original and registered image data.

As illustrated in Figure 5a, our evaluation begins by computing slice‐to‐slice difference images and applying Laplacian filtering to highlight rapid intensity changes. Noise characteristics are quantified using entropy and SNR, obtained by decomposing the Laplacian‐filtered image into structural and noise components via bilateral filtering. To assess structural preservation, we construct a multi‐scale Difference‐of‐Gaussians (DoG) pyramid and compute local contrast across scales (Figure 5b,c). Unregistered images exhibit high noise levels and weak structural continuity, whereas registered images show clearer cellular contours and consistently higher multi‐scale contrast. This combined noise–structure analysis enables simultaneous evaluation of distortion suppression and preservation of biological morphology, and is further validated on synthetic datasets with known ground truth (Figures S24 and S25). For a detailed description of the evaluation procedure, please refer to the Method Section 4.4.

#### 2.3.2 Performance Comparison of Different Registration Methods on Real Datasets

We compared vEMRec with the current state‐of‐the‐art 3D elastic registration algorithm, SEAMLeSS, across multiple real‐world datasets. TrakEM2 was not included in the real‐data evaluation because, as shown in Figure 4a, it exhibits an unfavorable accuracy–runtime trade‐off and requires extensive parameter tuning and computational resources when applied to large‐scale datasets. SEAMLeSS serves as a modern deep‐learning‐based baseline, providing a fair and scalable comparison for evaluating the performance of vEMRec.

The results in Figure 5 demonstrate that vEMRec consistently outperforms SEAMLeSS in both quantitative metrics and qualitative visualizations. First, vEMRec‐registered volumes show significantly higher contrast in the Difference of Gaussian (DoG) images (Figure 5e, left), indicating improved registration accuracy and better preservation of fine biological textures. This is crucial for downstream analysis, where structural details must be preserved. Moreover, Laplacian‐filtered images from vEMRec exhibit a 0.15 increase in SNR and approximately 2‐unit reduction in entropy compared to SEAMLeSS (Figure 5e, middle and right), suggesting effective suppression of nonlinear misalignments and random noise between adjacent slices. These improvements enhance the clarity and continuity of biologically relevant structures in the registered volumes. In addition, vEMRec results in a more compact and concentrated entropy distribution (Figure 5d). As registration corrects irregular distortions, the overall image complexity and uncertainty decrease, leading to tighter clustering of entropy values. Qualitative comparisons further support these findings. As illustrated in the side‐view visualizations (Figure 5f), vEMRec successfully corrects subtle nonlinear deformations and restores the true axial morphology of biological specimens. Notably, it recovers continuous cell membranes and intact vesicle structures, effectively removing distortion artifacts that persist in SEAMLeSS‐processed results. Together, these results strongly demonstrate the superior performance and robustness of vEMRec in real‐world registration tasks. Additional examples and comprehensive visualizations are provided in Figures S21 and S22.

### 2.4 Impact of vEMRec Alignment on the Performance of Downstream Tasks

#### 2.4.1 Performance on Downstream Isotropic Reconstruction Tasks

Acquiring high‐quality isotropic vEM data remains technically challenging, especially at large scales [38, 39]. For high‐resolution, large‐volume data, existing approaches are limited by two major factors: the severe axial discontinuities present in raw anisotropic vEM stacks and the substantial computational and memory demands of current isotropic reconstruction algorithms. Our experiments show that vEMRec significantly improves the structural continuity and fidelity of anisotropic volumes, enabling high‐resolution isotropic reconstruction even with a simple interpolation step (e.g., FIJI's default interpolation).

**FIGURE 6:**
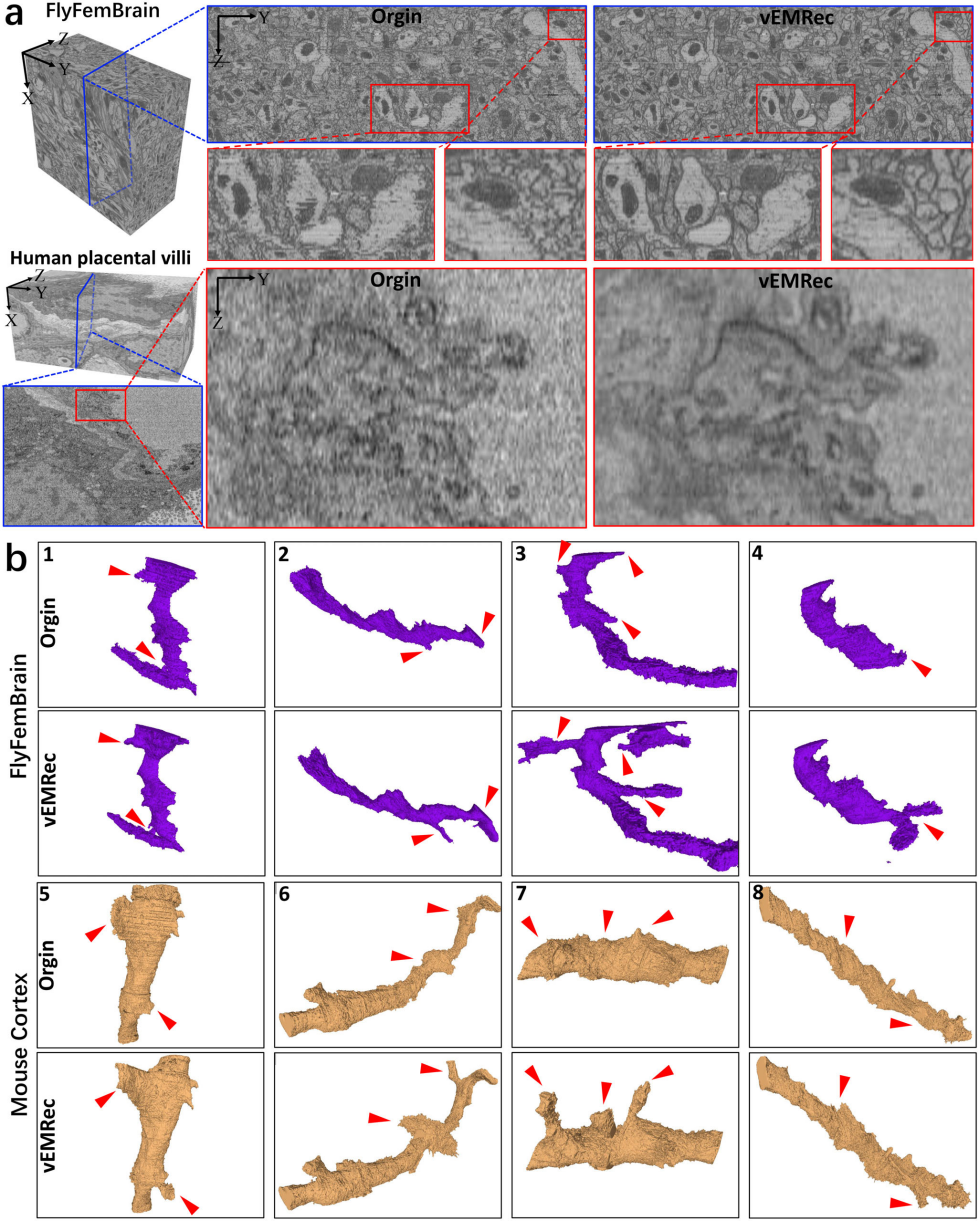
vEMRec enhances the performance of downstream tasks: isotropic reconstruction and 3D segmentation. (a) Side‐view visualization of isotropic reconstruction results on the FlyFemBrain and Human Term Placental Villi (SBF‐SEM) datasets. Even with a simple interpolation algorithm, the vEMRec‐registered data retain clearly discernible ultrastructural features. (b) Neuron segmentation results on the FlyFemBrain and mouse cortex datasets (additional segmentation visualizations are provided in Figure S35). Segmentation is performed on both raw and vEMRec‐registered data. The vEMRec‐aligned data yield more accurate branching structures, facilitating improved anatomical fidelity and potential scientific insights.

We conducted experiments on five real‐world datasets, including the Caenorhabditis elegans cell dataset [33], mouse cortical dataset [31], female fruit fly brain neural dataset [34], and the human term placental villi dataset [35]. (see Section 4.1 for dataset details). After applying vEMRec for 3D alignment, we interpolated the resulting volumes along the axial direction using a basic bilinear interpolation scheme. The resulting isotropic volumes exhibit excellent preservation of fine cellular structures such as membranes, mitochondria, and neurite boundaries, with clear continuity across slices (Figure 6a). Additional visualization results are provided in Figures S26– S33. These results strongly indicate that vEMRec effectively mitigates the nonlinear distortions and axial inconsistencies that typically hinder isotropic reconstruction. Moreover, the ability to generate high‐quality isotropic volumes with only minimal post‐processing significantly lowers the technical barrier for downstream analysis and visualization. Importantly, our findings suggest a paradigm shift: instead of relying on increasingly complex isotropic reconstruction algorithms, it is possible to achieve reliable and scalable isotropy through high‐fidelity anisotropic reconstruction followed by lightweight interpolation. This approach dramatically improves the practicality of isotropic vEM analysis, particularly for large‐scale datasets where conventional methods struggle with efficiency and scalability.

#### 2.4.2 Performance on Downstream Neuron 3D Segmentation Tasks

Three‐dimensional segmentation of ultrastructural features, especially neurons, is a key downstream task in vEM analysis and critically depends on the continuity and quality of the aligned image volume. In particular, axial discontinuities and nonlinear distortions can severely degrade segmentation accuracy. Therefore, high‐fidelity 3D alignment provides an essential foundation for reliable structural analysis.

To evaluate the effectiveness of our approach for this task, we conducted extensive experiments on three widely used, large‐scale neuron datasets, including the FAFB (Full Adult Fly Brain) [13], the mouse cortical dataset [31], and the female fruit fly brain neural dataset [34], (see Section 4.1 for dataset details). The raw images were first reconstructed using vEMRec, and, due to the anisotropic nature of the original datasets, we applied FIJI's interpolation function after alignment to generate isotropic volumes. We then performed 3D neuron segmentation using the segmentation tools provided by Microscopy Image Browser (MIB) [40] on both the raw and vEMRec‐reconstructed datasets. Our results show that vEMRec‐reconstructed volumes significantly improve neuron segmentation performance compared to the unprocessed raw data. More importantly, the vEMRec‐processed data enables the identification of a larger number of reliable and morphologically coherent synaptic branches, which are essential for connectomic analysis (Figure 6b and Figure S35). The segmentation results on vEMRec‐reconstructed data display enhanced structural continuity. Neurons exhibit smooth and continuous boundaries, effectively eliminating the jagged edge artifacts seen in the raw data (for example, the first comparison image from FlyFemBrain). In addition, the number and clarity of synaptic branches (highlighted by red arrows) are greatly improved, facilitating more accurate anatomical mapping and structural connectivity analysis.These findings highlight the substantial benefits of vEMRec for downstream computational analysis. By producing clean and morphologically accurate 3D alignment, vEMRec not only improves segmentation quality but also supports deeper insights into the ultrastructural organization of neural circuits, helping to accelerate biological discovery.

## 3 Discussion

In this study, we introduce vEMRec, an efficient 3D alignment algorithm, designed to eliminate rigid misalignment and nonlinear distortion, thereby restoring the true 3D structure of biological specimens. Our approach follows two key phases: first, a sequential rigid alignment to correct large‐scale misalignments; and second, Gaussian filtering to address nonlinear distortions while preserving the natural deformation. In the rigid alignment phase, we extract stable edge features from the images and match them to establish reliable point correspondences. These correspondences are then used to compute the rigid transformation parameters, ensuring precise 3D alignment, even in cases of significant misalignment. In the elastic registration phase, we introduce a 1D Gaussian filter into the 3D image stack. This filter effectively decouples nonlinear distortions from natural deformations in the frequency domain. Known for its noise reduction and smoothing capabilities, the Gaussian filter plays a central role in eliminating distortions while preserving the inherent deformation characteristics of biological tissues. Specifically, we use an optical flow estimation network to calculate dense displacement fields between slices. These displacement fields are then integrated with the Gaussian filter, producing a combined field that corrects the nonlinear distortions in each slice. By leveraging this method, we not only correct nonlinear distortions but also maintain the biological structure's integrity, providing a robust basis for further biological analysis. Additionally, we propose a multi‐scale difference filtering evaluation strategy that can assess real datasets without ground truth, enabling us to better understand and validate alignment performance.

Our method, for both 3D rigid alignment and 3D elastic registration, consistently outperforms existing approaches. We conducted comprehensive tests on datasets with varying degrees of distortion and diverse cellular structures. The results demonstrate that our method is highly robust in managing different data types and handling complex deformation environments. Additionally, to assess its practical applicability, we applied our method to the downstream tasks. Experimental results showed that our approach accurately restores the true structure of cellular tissues. This underscores the wide‐ranging potential of our method in real‐world biomedical analysis. However, it's important to acknowledge that our approach is built on the assumption that biological specimens exhibit smooth, low‐frequency 3D structures. While most biological structures do display these characteristics, some tissues and cells possess complex folds and twists, which can pose challenges for accurate 3D structure recovery. To address high‐frequency nonlinear distortions, we utilized a straightforward yet efficient 1D Gaussian filtering approach. Although this method effectively removes most high‐frequency distortions, some mid‐frequency nonlinear distortions, which resemble natural deformations, may still persist. Therefore, a key area for future research is developing adaptive smoothing filters that can better address both mid‐ and high‐frequency nonlinear distortions, further improving the accuracy and stability of 3D structure recovery.

Traditional alignment algorithms typically process each pair of adjacent slices using a Coarse‐to‐Fine neural network (e.g., multi‐scale convolutional architectures such as U‐Net) to estimate a deformation field, which can be expressed as:

(1) $$\begin{array}{c} \Phi_{i}=F_{\theta }\left(I_{i},I_{i-1}\right) \end{array}$$

where $\Phi_{i}$ denotes the deformation field, $I_{i}$ and $I_{i-1}$ are the paired slices, and $F_{\theta }$ represents the neural network. The multi‐scale design of $F_{\theta }$ enables the model to capture structural information at different spatial levels in a hierarchical Coarse‐to‐Fine manner, thereby improving deformation accuracy. However, regardless of how precisely $\Phi_{i}$ is estimated, such approaches remain fundamentally constrained by the pairwise optimization objective:

(2) $$\begin{array}{c} \Phi_{i}=\arg\min_{\Phi_{i}}\;\text{Loss}\left(I_{i},I_{i-1},\Phi_{i}\right)+\lambda \;{∥\nabla \Phi_{i}∥}_{2}^{2} \end{array}$$

Here, the Loss term measures the similarity (e.g., mean squared difference) between $I_{i}$ and $I_{i-1}$, while the regularization term enforces deformation smoothness. This formulation encourages adjacent slices to become as similar as possible, a reasonable assumption for medical image registration [41], where both images depict nearly identical anatomy. In contrast, for 3D volume electron microscopy (vEM) datasets, consecutive slices correspond to physically distinct tissue sections that naturally exhibit smooth morphological variations. Thus, rather than enforcing pixel‐level similarity, the objective should eliminate nonlinear distortions while preserving intrinsic biological deformations reflecting the genuine structural continuity along the z‐axis. While the Coarse‐to‐Fine network design can effectively remove nonlinear distortions, its similarity‐driven optimization tends to over‐align slices, suppressing low‐frequency morphological variations that represent true biological changes and thereby compromising structural fidelity in the reconstructed volume.

In contrast, the proposed frequency decoupling strategy addresses these limitations by jointly modeling the entire image stack along the z‐axis instead of treating slice pairs independently. Although nonlinear distortions and natural deformations may appear inseparable when viewed between a single pair of slices, their distinct properties become evident when analyzed across the entire volumetric sequence. Natural biological deformations exhibit smooth, manifold‐like variations along the z‐dimension, corresponding to low‐frequency components, whereas nonlinear distortions appear as abrupt geometric perturbations, corresponding to high‐frequency components. This frequency‐domain distinction provides a principled way to separate true structural continuity from artificial distortions. Inspired by the classical concept of 1D Gaussian filtering, which removes high‐frequency noise while preserving low‐frequency trends, we design a Gaussian filter–based 3D elastic registration algorithm that treats the z‐axis as a temporal dimension. This formulation explicitly enables the decoupling of nonlinear distortions from natural deformations, thereby preserving biologically meaningful structural continuity while effectively removing undesirable high‐frequency artifacts across the reconstructed volume.

In conclusion, vEMRec focuses on eliminating rigid misalignment and nonlinear distortion while preserving the natural deformation in vEM image stacks, with the goal of accurately restoring the 3D structure of biological specimens. This lays a solid foundation for researchers to explore the structure and function of cellular tissues at the nanoscale. By achieving more precise 3D biological structure alignment, researchers can more effectively identify and analyze the microscopic structures of biological specimens. This not only deepens our understanding of the spatial organization and functional connections within cellular tissues, but also provides comprehensive 3D data support for future biomedical research. With its exceptional registration performance and ability to adapt to complex deformations and cumulative errors, vEMRec serves as an essential tool for analyzing a continuous sequence electron microscopy images. It offers robust technical support for advancing cutting‐edge life science research and driving innovation in the biomedical field.

## 4 Methods

### 4.1 Datasets of Volume Electron Microscopy and Metrics

Both synthetic and real‐world datasets are utilized to evaluate the performance of our 3D alignment algorithm. For synthetic dataset, We selected six publicly available datasets from the OpenOrganelle platform [3] where the high‐resolution images provide a reliable reference. These datasets include electron microscope images of various mouse tissues, including the heart, kidney, liver, skin, and pancreas, enabling us to comprehensively assess the applicability and robustness of our method across different representative tissue types. For real‐world data, we evaluated our method on several publicly available large‐scale volumetric EM datasets acquired using different imaging modalities. Our real‐world evaluation includes not only widely used neuronal connectomics datasets, but also non‐neuronal datasets, which together represent diverse biological structures encountered in volume EM imaging. Specifically, we utilized the FAFB (Full Adult Fly Brain, ssTEM/TEMCA) dataset [13] with patches of 8192×8192 pixels, the Caenorhabditis elegans cell dataset (TEM) [33] with image sizes of 21504×26624 pixels, the mouse cortical dataset (ATUM‐SEM) [31] with images of 24576×20480 pixels, the female fruit fly brain neural dataset (TEM) [34] with 8192×8192 pixels, and the human term placental villi dataset (SBF‐SEM) [35] with 3000×3000 pixels. These large‐scale real‐world datasets, covering a wide range of cellular structures and biological variability, were chosen to demonstrate the robustness of vEMRec across diverse biological scenarios and to assess its scalability and reliability in handling massive real‐world EM data. Detailed information on all datasets used in our experiments is provided in Tables S1 and S2.

To quantitatively evaluate the performance of our method, we calculated several metrics, including Normalized Cross‐Correlation (NCC), Mutual Information (MI), and Structural Similarity Index (SSIM) between the registrated image series and the ground truth (GT). These metrics provide a comprehensive evaluation of the accuracy of our alignment and registration results from different perspectives. Specifically, NCC measures the similarity between the result images and the GT, MI reflects the mutual information between the two, and SSIM evaluates the accuracy based on structural information. Additionally, to assess the applicability and robustness of our method for the downstream task of 3D segmentation, we computed the Dice coefficient and Hausdorff Distance between the segmented output labels and the ground truth labels. This comprehensive evaluation is aimed to verify the accuracy and reliability of vEMRec in reconstructing the correct 3D structure of biological specimens and further demonstrate its effectiveness in downstream tasks.

### 4.2 3D Rigid Alignment

The vEM 3D rigid alignment workflow involves several key steps, First, for each adjacent slice pair $\left\{I_{t-1},I_{t}\right\}$, we employ a robust feature point extraction method to find keypoints, which are refined with an edge mask. Bidirectional nearest neighbor matching is then used to align feature points between slices, followed by the Locality Preserving Matching (LPM) [29] algorithm to constrain the geometric consistency. The rigid transformation parameters between adjacent slices are subsequently estimated using these filtered matches, and the resulting transformation is applied to each slice $I_{t}$. For clarity, the symbols are provided in Table S12.

#### 4.2.1 Edge Feature Extraction

A sufficient number of feature points is crucial for capturing the key characteristics of images, and precise point set matching plays a decisive role in reliably identifying similar regions within the input images. However, natural deformations in biological cells between adjacent images can significantly disrupt the feature point matching process. To overcome this challenge, we apply the SuperPoint feature extraction algorithm [28] to extract keypoints from the source image $I_{t-1}$ and the target image $I_{t}$. We then use edge detection [42] to filter and obtain the edge keypoint sets ${\left\{S_{i}\right\}}_{i=0}^{N}$ and ${\left\{T_{i}\right\}}_{i=0}^{N}$. Since natural deformations along the edges of biological structures are typically minimal, these edge feature points are more stable, thus improving the reliability of the matching process.

#### 4.2.2 Bidirectional Nearest Matching

We use a bi‐directional matching approach to establish initial correspondences between keypoints in the source image $I_{t-1}$ and the target image $I_{t}$. Our approach begins with nearest‐neighbor matching from ${\left\{S_{i}\right\}}_{i=0}^{N}$ to ${\left\{T_{i}\right\}}_{i=0}^{N}$, followed by reverse matching from ${\left\{T_{i}\right\}}_{i=0}^{N}$ to ${\left\{S_{i}\right\}}_{i=0}^{N}$. We assign a binary value δ(i,j) to each feature match $\left(S_{i},T_{j}\right)$: δ(i,j)=1 indicates a validated bidirectional match, suggesting greater accuracy and stability, while δ(i,j)=0 denotes an unreliable match that is discarded. The formal definition of δ(i,j) is given in Equation (3):

(3) $$\begin{array}{c} \delta \left(i,j\right)=NN\left(S_{i},T_{j}\right)\&\&NN\left(T_{j},S_{i}\right) \end{array}$$

where NN(·) returns a boolean indicating if the latter point is the nearest neighbor of the former. If the descriptor of point $S_{i}$ has the minimum Euclidean distance to the descriptor of $T_{j}$, then $NN(T_{j},S_{i})=1$.

#### 4.2.3 Geometric Consistency Constraints

In electron microscopy images, the prevalence of dense and repetitive small structures often results in a significant number of incorrect feature matches. These challenges are further exacerbated by natural deformations between consecutive slices, making precise matching even more difficult. However, our observations indicate that, despite these local deformations, the distribution of feature points along structural edges remains topologically consistent. In other words, the geometric configurations formed by feature points along these edges tend to exhibit a high degree of similarity across images. To effectively address the challenges posed by local deformations, we leverage the Locally Preserving Matching (LPM) algorithm [29], which is based on maintaining the local neighborhood structure of potential true matches. This method enhances the stability of feature matching by maintaining the spatial relationships among feature points, thereby ensuring more reliable and robust correspondences even in the presence of natural deformations. Finally, we filter the source point set ${\left\{S_{i}\right\}}_{i=0}^{N}$ and the target point set ${\left\{T_{i}\right\}}_{i=0}^{N}$ to obtain robust feature matches X and Y.

#### 4.2.4 Rigid Transformation Matrix Estimation

After obtaining the well‐matched point sets X and Y, we use Singular Value Decomposition (SVD) [43] to compute the closed‐form rigid transformation parameters, as shown in Equation (4):

(4) $$\left\{\begin{array}{c} S=XY^{T},\\ U\Sigma V=S,\\ R_{t}=VU^{T},\\ t_{t}=u-R_{t}v \end{array}\right.$$

Where UΣV is the SVD matrix, $R_{t}$ is the rotation matrix, $t_{t}$ is the translation vector, and u and v represent the centroids of point sets X and Y, respectively.

The obtained parameters $R_{t}$ and $t_{t}$ are then applied to perform the rigid transformation on the $t^{th}$ image in the image sequence, as shown in Equation (5).

(5) $$\begin{array}{c} \bar{I_{t}}=R_{t}I_{t}+t_{t},\;t=1,2,\text{…},n \end{array}$$

where $\bar{I_{t}}$ represents the aligned image.

### 4.3 3D Elastic Registration

The primary goal of 3D elastic registration is to correct non‐linear distortions that cannot be addressed by rigid alignment, thereby restoring z‐axial continuity and ensuring accurate reconstruction of the 3D structure of biological tissues. In this work, we extend a Gaussian filtering‐based 3D elastic registration method to effectively distinguish between nonlinear distortions and natural deformations in the frequency domain by decoupling high‐frequency and low‐frequency information [44]. Specifically, we developed a cascaded pyramid optical flow estimation network to incrementally estimate deformation fields between adjacent slices. Then, by extending the 1D Gaussian filtering technique to 3D image stacks, we leverage its robust smoothing capabilities to efficiently remove nonlinear distortions while preserving natural deformations. Finally, the estimated deformation fields are integrated using Gaussian filtering to achieve precise distortion correction and restoration of the true structure.

#### 4.3.1 Problem Formulation

In the problem of 3D elastic registration, we work with a stack of images, ${\left\{I_{i}\right\}}_{i=0}^{N}$, that are distorted due to non‐linear deformations caused by sample slicing or optical effects. The goal of 3D vEM registration is to determine a series of deformation fields, ${\left\{\varphi_{i}\right\}}_{i=0}^{N}$, between adjacent slices to correct these distortions, enabling accurate 3D structure reconstruction. For this, the deformation fields $\varphi_{i}$ must not only eliminate non‐linear distortions effectively but also preserve the natural axial deformations present in the images. We address this from a frequency domain perspective, treating axial natural deformations as low‐frequency components and non‐linear distortions as high‐frequency components. This perspective allows us to identify the optimal deformation field $\varphi_{i}$ that smooths out non‐linear distortions using a specially designed Gaussian filter. Based on this approach, we define the following objective function:

(6) $$\begin{array}{c} \varphi_{i}=\arg\min_{\varphi_{i}}\sum_{\forall I_{j}\in N\left(I_{i}\right)}F\left(I_{i},I_{j},\varphi_{i}\right)+\lambda ||\nabla \varphi_{i}{\left|\right|}_{2}^{2} \end{array}$$

where $I_{i}$ denotes the slice to be registered, $N(I_{i})$ represents the neighboring slices of $I_{i}$, function F estimates the difference between the $I_{i}$ deformed by $\varphi_{i}$ and the neighboring slice $I_{j}$, and $\lambda ||\nabla \varphi_{i}{\left|\right|}_{2}^{2}$ acts as a regularization term.

#### 4.3.2 Optical Flow Estimation Network

We adopted an optical flow estimation network to compute the deformation field between slices. The network architecture is shown in Figure 1d and Figure S38. Using a cascaded design, the network takes as input the fixed image $I_{f}$ and the moving image $I_{m}$, and progressively estimates the deformation field. The generation of the deformation field $\varphi_{m,f}$ is divided into T iterations. In each iteration t, the input comprises the fixed image $I_{f}$ and the warped image ${\hat{I}}_{m,t-1}$, which is registered by the deformation field $\varphi_{m,f,t-1}$ from the previous iteration. We first build contextual feature pyramids ${\left\{C_{f}^{s}\right\}}_{s=1}^{3}$, feature pyramids ${\left\{F_{f}^{s}\right\}}_{s=0}^{3}$ and ${\left\{F_{m}^{s}\right\}}_{s=0}^{3}$ using 2D convolutional encoder. ${\left\{F_{f}^{s}\right\}}_{s=0}^{3}$ and ${\left\{F_{m}^{s}\right\}}_{s=0}^{3}$ are employed to generate deformation fields for each layer, while ${\left\{C_{f}^{s}\right\}}_{s=1}^{3}$ provides prior information for field generation. The deformation field $\varphi_{m,f,t}^{s+1}$ from the previous layer is upsampled and fed into the next layer, yielding the updated field $\varphi_{m,f,t}^{s}$. $\varphi_{m,f,t}^{1}$ is then input into a fusion module along with $\left\{F_{f}^{0},F_{m}^{0}\right\}$, producing the final deformation field $\varphi_{m,f,t}$ for the current iteration. The details of the network structure and training and fine‐tuning procedure can be found in Sections S3 and S4.

#### 4.3.3 Gaussian Filter in Simple 1D Model

We first demonstrate our method on a simple 1D model and subsequently extend it to 3D image stacks. Given a 1D signal s of length N, and a Gaussian kernel g with the radius r=1, the filtering operation at point i is expressed as follows:

(7) $$\begin{array}{c} \hat{s_{i}}=g_{-1}s_{i-1}+g_{0}s_{i}+g_{1}s_{i+1} \end{array}$$

where $g_{k}$ denotes the weight of the k‐th element of the Gaussian kernel, $s_{i}$ represents the i‐th value of the signal, and ${\hat{s}}_{i}$ represents the filtered value at position i.

Let us define $d_{i,j}=s_{j}-s_{i}$ for any indices i and j, then Equation(7) can be rewritten as:

(8) $$\begin{array}{c} \hat{s_{i}}=g_{-1}d_{i,i-1}+g_{0}d_{i+1,i}+g_{-1}s_{i}+\left(g_{0}+g_{1}\right)s_{i+1} \end{array}$$

#### 4.3.4 Gaussian Filter in 3D Image Stacks

Let's consider the application of a similar 1D Gaussian filter to a 3D image stack, where each point $s_{i}$ in a 1D signal is analogous to an image $I_{i}$ in the image stack. Here, $d_{i,j}$ represents the deformation field $\varphi_{i,j}$ that registers slice $I_{i}$ to slice $I_{j}$. Therefore, the Gaussian filter operation for slice $I_{i}$ can be expressed as:

(9) $$\begin{array}{c} \hat{I_{i}}=g_{-1}\varphi_{i,i-1}+g_{0}\varphi_{i+1,i}+g_{-1}I_{i}+\left(g_{0}+g_{1}\right)I_{i+1} \end{array}$$

where we assume the radius of the Gaussian kernel to be 1, $I_{i}$ represents the i‐th slice, $\varphi_{i,j}$ represents the deformation field from slice $I_{i}$ to slice $I_{j}$.

Intuitively, however, this formula is not directly computable, since deformation fields and image intensities cannot be added together. Therefore, we reorganize the expression as:

(10) $$\begin{array}{c} \hat{I_{i}}=\left(g_{-1}I_{i}+g_{-1}\varphi_{i,i-1}\right)+\left(\left(g_{0}+g_{1}\right)I_{i+1}+g_{0}\varphi_{i+1,i}\right) \end{array}$$

To make this formulation mathematically meaningful, we replace the addition between the image and deformation field with the composition operation ∘, which warps the image according to the deformation field. This leads to:

(11) $$\begin{array}{c} \hat{I_{i}}=g_{-1}I_{i}\circ g_{-1}\varphi_{i,i-1}+\left(g_{0}+g_{1}\right)I_{i+1}\circ g_{0}\varphi_{i+1,i} \end{array}$$

According to Equation(11), it can be observed that $\hat{I_{i}}$ incorporates weighted contributions from both the deformed $I_{i}$ and the deformed $I_{i+1}$. This unfortunately introduces image information from $I_{i+1}$, which is not desirable. To retain the true information only from $I_{i}$, we made adjustment to Equation(11). Specifically, we deform $I_{i}$ using a weighted deformation field calculated among multiple adjacent slices, rather than directly using information from adjacent slices. The deformation fields for $I_{i}$ with adjacent slices are calculated based on the registered adjacent slices. Therefore, the calculation for the deformed $\hat{I_{i}}$ is as follows:

(12) $$\begin{array}{c} \left\{\begin{array}{cc} & I_{i}^{tmp}=I_{i}\circ g_{-1}\varphi_{i,i-1},\\ & I_{i+1}^{tmp}=I_{i+1}\circ g_{0}\varphi_{i+1,i},\\ & {\bar{g}}_{0}^{tmp}=\frac{\left(g_{-1}+g_{0}\right)}{\left(g_{-1}+g_{0}\right)+\left(g_{0}+g_{1}\right)},\\ & {\bar{g}}_{1}^{tmp}=\frac{\left(g_{0}+g_{1}\right)}{\left(g_{-1}+g_{0}\right)+\left(g_{0}+g_{1}\right)},\\ & \hat{I_{i}}=I_{i}\circ \frac{\varphi_{i,i-1}+{\bar{g_{0}}}^{tmp}\varphi_{i,i}^{tmp}+{\bar{g_{1}}}^{tmp}\varphi_{i,i+1}^{tmp}}{2} \end{array}\right. \end{array}$$

where we denote the deformed $\left\{I_{i},I_{i+1}\right\}$ as intermediate images ${\left\{I_{i+k}^{tmp}\right\}}_{k=0}^{1}$, and their corresponding normalized weights as ${\left\{{\bar{g}}_{k}^{tmp}\right\}}_{k=0}^{1}$. Then, we register $I_{i}$ to ${\left\{I_{i+k}^{tmp}\right\}}_{k=0}^{1}$ to obtain ${\left\{\varphi_{i,i+k}^{tmp}\right\}}_{k=0}^{1}$. After being weighted by ${\left\{{\bar{g}}_{k}^{tmp}\right\}}_{k=0}^{1}$, ${\left\{\varphi_{i,i+k}^{tmp}\right\}}_{k=0}^{1}$ are used for final registration to obtain $\hat{I_{i}}$.

We sequentially apply the Gaussian filter operation to the entire image stacks. Through doing that, we can eliminate minor nonlinear distortions without erroneously removing natural deformations. This ensures z‐axial continuity of the image stack and restores the correct 3D structure of the biological specimen.

### 4.4 Multi‐Scale Difference Filtering Evaluation

To address the issue of the absence of ground truth in real datasets, we developed a multi‐scale difference filtering evaluation strategy. Specifically, given two adjacent slices $I_{i}$ and $I_{i+1}$, we first compute a differential image

(13) $$\begin{array}{c} D=I_{i+1}-I_{i} \end{array}$$

and then process it using Laplacian filtering. Due to pixel‐level nonlinear distortions between poorly registered adjacent images, small noise appears in the differential image D. We employ the Laplacian operator $\nabla^{2}$ to make the noise more pronounced, thereby we get the Laplacian‐filtered image $\nabla^{2}D$. We assess the results from two perspectives: noise reduction and preservation of biological structural information.

For noise, we measure the image entropy H of the Laplacian‐filtered image $\nabla^{2}D$:

(14) $$\begin{array}{c} H=-\sum_{i=0}^{255}p\left(\nabla^{2}D_{i}\right)\log_{2}\left(p\left(\nabla^{2}D_{i}\right)\right) \end{array}$$

where $p\left(\nabla^{2}D_{i}\right)=\frac{n_{i}}{N}$, $n_{i}$ is the number of occurrences of the i‐th pixel, and N is the total number of pixels.

We also use the signal‐to‐noise ratio (SNR) to measure the amount of noise in $\nabla^{2}D$. Specifically, we apply bilateral filtering [45] to $\nabla^{2}D$ to obtain the filtered image $I_{f}$ and the noise image $I_{n}$, and then we calculate the ratio of the variances of $I_{f}$ and $I_{n}$ as the signal‐to‐noise ratio:

(15) $$\begin{array}{c} \left\{\begin{array}{cc} & I_{f}=BilateralFilter\left(\nabla^{2}D\right),\\ & I_{n}=\nabla^{2}D-I_{f},\\ & SNR=\frac{Var(I_{f})}{Var(I_{n})} \end{array}\right. \end{array}$$

For the preservation of biological structural information, since the registration reduces the nonlinear errors between adjacent slices, it reduces the noise in the difference image, and makes the biological structure smoother. Therefore, the differential images between adjacent slices with good registration often have clearer biological structures and show higher contrast at different scales. Hence, we established a Difference of Gaussian(DoG) pyramid and calculated the contrast across multiple scales to gauge the richness of the retained textures. Specifically, we first construct a 4‐layer Difference of Gaussian pyramid ${\left\{DoG_{i}\right\}}_{i=0}^{3}$ for $\nabla^{2}D$:

(16) $$\begin{array}{c} \left\{\begin{array}{cc} & G_{i}\left(x,y\right)=\frac{1}{2\pi \sigma_{i}^{2}}e^{-\frac{x^{2}+y^{2}}{2\sigma_{i}^{2}}},\;i=0,1,2,3,4,\\ & DoG_{i}=\nabla^{2}D*G_{i}-\nabla^{2}D*G_{i+1},\;i=0,1,2,3 \end{array}\right. \end{array}$$

where $G_{i}\left(x,y\right)$ represents the Gaussian kernel at the i‐th layer, and $DoG_{i}$ represents the Difference of Gaussian image at the i‐th layer.

Then, we use the gray level co‐occurrence matrix (GLCM) [46] to calculate the contrast $Con_{i}$ for that layer on the Difference of Gaussian image $DoG_{i}$:

(17) $$\begin{array}{c} \left\{\begin{array}{cc} & GLCM_{i}=graycomatrix\left(DoG_{i}\right),\;i=0,1,2,3,\\ & Con_{i}=\sum_{k,j=0}^{255}{\left(k-j\right)}^{2}GLCM_{i}\left(k,j\right),\;i=0,1,2,3 \end{array}\right. \end{array}$$

where $GLCM_{i}$ is the gray level co‐occurrence matrix corresponding to the Difference of Gaussian image at the i‐th layer, and $Con_{i}$ is the corresponding contrast.

## Author Contributions

This work is a collaborative study carried out by Zhenbang Zhang (Shandong University) during his visiting period at Beijing Institute of Technology. R.H. F.Z. and Z.Z. concepted the project and methods. Z.Z. performed the majority of the research, including code development, implementation, extensive experimentation, data analysis, and manuscript drafting. H.L. assisted with experimental validation and contributed to manuscript writing and revision. Z.Y., D.S., and Z.X. provided technical support and contributed to result interpretation. X.G., F.Z., and R.H. jointly supervised the project and guided the overall research direction. All authors reviewed and approved the final manuscript.

## Funding

This research was supported by the National Natural Science Foundation of China Projects Grant [W2511070], the Dubai Future Foundation (Award No. 2024CANAD‐MES‐061), the National Key Research and Development Program of China [2021YFF0704300], the Natural Science Foundation of Shandong Province [ZR2023YQ057], the Fundamental Research Funds for the Central Universities, the King Abdullah University of Science and Technology (KAUST) Office of Research Administration (ORA) under Award No REI/1/5289‐01‐01, REI/1/5992‐01‐01, URF/1/6713‐01‐01, FCC/1/5932‐12‐11, URF/1/6599‐01‐02, FCC/1/5940‐20‐03, Center of Excellence for Smart Health (KCSH), under award number 5932, and Center of Excellence on Generative AI, under award number 5940.

## Conflicts of Interest

The authors declare no conflicts of interest.

## Code Availability

All inference and training code for vEMRec is available at https://github.com/zhangzhenbang2021/vEMRec.git.

## Supporting information


**Supporting File 1**: advs74367‐sup‐0001‐SuppMat.pdf.

**Supporting File 2**: advs74367‐sup‐0002‐SuppMat.pdf.

## Data Availability

The synthetic datasets used by vEMRec can be obtained from https://openorganelle.janelia.org/. The liver dataset is available at https://openorganelle.janelia.org/datasets/jrc_mus‐liver , the skin dataset can be found at https://openorganelle.janelia.org/datasets/jrc_mus‐skin‐1 , and the kidney dataset is available at https://openorganelle.janelia.org/datasets/jrc_mus‐kidney. The liver‐3 dataset can be accessed at https://openorganelle.janelia.org/datasets/jrc_mus‐liver‐3 , the pancreas dataset is located at https://openorganelle.janelia.org/datasets/jrc_mus‐pancreas‐4 , and the heart dataset can be found at https://openorganelle.janelia.org/datasets/jrc_mus‐heart‐1. For the large real dataset FAFB used by vEMRec, visit https://temca2data.org/. The C. elegans dataset is available at https://neurodata.io/data/bumbarger13/ , and the mouse cortex dataset can be found at https://neurodata.io/data/kasthuri15/. The SBF SEM of Human term placental villi dataset is available at https://www.ebi.ac.uk/empiar/EMPIAR‐10967/ .

## References

[1] M. Terasaki , T. Shemesh , N. Kasthuri , et al., “Stacked Endoplasmic Reticulum Sheets Are Connected by Helicoidal Membrane Motifs,” Cell no. 2 (2013): 285–296. 10.1016/j.cell.2013.06.031 PMC3767119 23870120

[2] A. Müller , D. Schmidt , C. S. Xu , et al., “3D FIB‐SEM Reconstruction of Microtubule–Organelle Interaction in Whole Primary Mouse β Cells,” Journal of Cell Biology 220, no. 2 (2021): e202010039. 33326005 10.1083/jcb.202010039 PMC7748794

[3] C. S. Xu , S. Pang , G. Shtengel , et al., “An Open‐Access Volume Electron Microscopy Atlas of Whole Cells and Tissues,” Nature 599, no. 7883 (2021): 147–151. 34616045 10.1038/s41586-021-03992-4 PMC9004664

[4] J. L. Morgan and J. W. Lichtman , “An Individual Interneuron Participates in Many Kinds of Inhibition and Innervates Much of the Mouse Visual Thalamus,” Neuron 106, no. 3 (2020): 468–481. 32142646 10.1016/j.neuron.2020.02.001 PMC7295017

[5] V. Baena and M. Terasaki , “Three‐Dimensional Organization of Transzonal Projections and Other Cytoplasmic Extensions in the Mouse Ovarian Follicle,” Scientific Reports 9, no. 1 (2019): 1262. 30718581 10.1038/s41598-018-37766-2 PMC6362238

[6] M. Nagai , S. Saitoh , T. Takaki , et al., “Glomerular Cellular Interactions Following Disruption of the Glomerular Basement Membrane in IgA Nephropathy: Ultrastructural Analyses by 3‐Dimensional Serial Block‐Face Scanning Electron Microscopy,” Kidney Medicine 2, no. 2 (2020): 222–225. 32734243 10.1016/j.xkme.2019.11.003 PMC7380390

[7] B. D. de Senneville , F. Z. Khoubai , M. Bevilacqua , et al., “Deciphering Tumour Tissue Organization by 3D Electron Microscopy and Machine Learning,” Communications Biology 4, no. 1 (2021): 1390. 34903822 10.1038/s42003-021-02919-z PMC8668903

[8] B. E. Johnson , A. L. Creason , J. M. Stommel , et al., “An Omic and Multidimensional Spatial Atlas from Serial Biopsies of an Evolving Metastatic Breast Cancer,” Cell Reports Medicine 3, no. 2 (2022): 100525. 35243422 10.1016/j.xcrm.2022.100525 PMC8861971

[9] N. Weidner , R. J. Cote , S. Suster , and L. M. Weiss , Modern Surgical Pathology E‐Book (Elsevier Health Sciences, 2009).

[10] A. Shoemark , M. Boon , C. Brochhausen , Z. Bukowy‐Bieryllo , et al., “International Consensus Guideline for Reporting Transmission Electron Microscopy Results in the Diagnosis of Primary Ciliary Dyskinesia (BEAT PCD TEM Criteria),” European Respiratory Journal 55, no. 4 (2020): 1900725. 32060067 10.1183/13993003.00725-2019

[11] A. Shoemark , T. Burgoyne , R. Kwan , et al., “Primary Ciliary Dyskinesia with Normal Ultrastructure: Three‐Dimensional Tomography Detects Absence of DNAH11,” European Respiratory Journal 51, no. 2 (2018): 1701809. 29467202 10.1183/13993003.01809-2017

[12] T. Takaki , N. Ohno , S. Saitoh , M. Nagai , and K. Joh , “Podocyte Penetration of the Glomerular Basement Membrane to Contact on the Mesangial Cell at the Lesion of Mesangial Interposition in Lupus Nephritis: A Three‐Dimensional Analysis by Serial Block‐Face Scanning Electron Microscopy,” Clinical and Experimental Nephrology 23 (2019): 773–781. 30734164 10.1007/s10157-019-01701-0

[13] Z. Zheng , J. S. Lauritzen , E. Perlman , et al., “A Complete Electron Microscopy Volume of the Brain of Adult Drosophila Melanogaster,” Cell 174, no. 3 (2018): 730–743. 30033368 10.1016/j.cell.2018.06.019 PMC6063995

[14] W. Yin , D. Brittain , J. Borseth , et al., “A Petascale Automated Imaging Pipeline for Mapping Neuronal Circuits with High‐Throughput Transmission Electron Microscopy,” Nature Communications 11, no. 1 (2020): 4949. 10.1038/s41467-020-18659-3 PMC7532165 33009388

[15] A. Wilson and M. Babadi , “SynapseCLR: Uncovering Features of Synapses in Primary Visual Cortex Through Contrastive Representation Learning,” Patterns 4, no. 4 (2023): 100693. 37123442 10.1016/j.patter.2023.100693 PMC10140600

[16] A. Shapson‐Coe , M. Januszewski , D. R. Berger , et al., “A Connectomic Study of a Petascale Fragment of Human Cerebral Cortex,” BioRxiv (2021): 2021–05.

[17] The MICrONS Consortium , "Functional Connectomics Spanning Multiple Areas of Mouse Visual Cortex," Nature 640 435–447 (2025), 10.1038/s41586-025-08790-w. 40205214 PMC11981939

[18] S. Dorkenwald , C. M. Schneider‐Mizell , D. Brittain , et al., “CAVE: Connectome Annotation Versioning Engine,” Nature Methods 22 (2025): 1–9. 40205066 10.1038/s41592-024-02426-z PMC12074985

[19] C. R. Gamlin , C. M. Schneider‐Mizell , M. Mallory , et al., “Connectomics of Predicted SST Transcriptomic Types in Mouse Visual Cortex,” Nature 640, no. 8058 (2025): 497–505. 40205210 10.1038/s41586-025-08805-6 PMC11981948

[20] B. Celii , S. Papadopoulos , Z. Ding , et al., “NEURD Offers Automated Proofreading and Feature Extraction for Connectomics,” Nature 640, no. 8058 (2025): 487–496. 40205208 10.1038/s41586-025-08660-5 PMC11981913

[21] A. J. Kievits , R. Lane , E. C. Carroll , and J. P. Hoogenboom , “How Innovations in Methodology Offer New Prospects for Volume Electron Microscopy,” Journal of Microscopy 287, no. 3 (2022): 114–137. 35810393 10.1111/jmi.13134 PMC9546337

[22] C. J. Peddie , C. Genoud , A. Kreshuk , et al., “Volume Electron Microscopy,” Nature Reviews Methods Primers 2, no. 1 (2022): 51. 10.1038/s43586-022-00131-9 PMC7614724 37409324

[23] S. Saalfeld , A. Cardona , V. Hartenstein , and P. Tomančák , “As‐Rigid‐As‐Possible Mosaicking and Serial Section Registration of Large SSTEM Datasets,” Bioinformatics 26, no. 12 (2010): i57–i63. 20529937 10.1093/bioinformatics/btq219 PMC2881403

[24] S. Saalfeld , R. Fetter , A. Cardona , and P. Tomancak , “Elastic Volume Reconstruction from Series of Ultra‐Thin Microscopy Sections,” Nature Methods 9, no. 7 (2012): 717–720. 22688414 10.1038/nmeth.2072

[25] T. Xin , Y. Lv , H. Chen , et al., “A Novel Registration Method for Long‐Serial Section Images of EM with a Serial Split Technique Based on Unsupervised Optical Flow Network,” Bioinformatics 39, no. 8 (2023): btad436. 37462605 10.1093/bioinformatics/btad436 PMC10403427

[26] S. Popovych , T. Macrina , N. Kemnitz , et al., “Petascale Pipeline for Precise Alignment of Images from Serial Section Electron Microscopy,” Nature Communications 15, no. 1 (2024): 289. 10.1038/s41467-023-44354-0 PMC10767115 38177169

[27] L. Heinrich , D. Bennett , D. Ackerman , et al., “Whole‐Cell Organelle Segmentation in Volume Electron Microscopy,” Nature 599, no. 7883 (2021): 141–146. 34616042 10.1038/s41586-021-03977-3

[28] D. DeTone , T. Malisiewicz , and A. Rabinovich , “SuperPoint: Self‐Supervised Interest Point Detection and Description,” in Proceedings of the IEEE Conference on Computer Vision and Pattern Recognition Workshops (2018): 224–236.

[29] J. Ma , J. Zhao , J. Jiang , H. Zhou , and X. Guo , “Locality Preserving Matching,” International Journal of Computer Vision 127 (2019): 512–531.

[30] M. D. Guay , Z. A. Emam , A. B. Anderson , et al., “Dense Cellular Segmentation for EM Using 2D–3D Neural Network Ensembles,” Scientific Reports 11, no. 1 (2021): 2561. 33510185 10.1038/s41598-021-81590-0 PMC7844272

[31] N. Kasthuri , K. J. Hayworth , D. R. Berger , et al., “Saturated Reconstruction of a Volume of Neocortex,” Cell 162, no. 3 (2015): 648–661. 26232230 10.1016/j.cell.2015.06.054

[32] F. Xu , Y. Shen , L. Ding , et al., “High‐Throughput Mapping of a Whole Rhesus Monkey Brain at Micrometer Resolution,” Nature Biotechnology 39, no. 12 (2021): 1521–1528. 10.1038/s41587-021-00986-5 34312500

[33] D. J. Bumbarger , M. Riebesell , C. Rödelsperger , and R. J. Sommer , “System‐Wide Rewiring Underlies Behavioral Differences in Predatory and Bacterial‐Feeding Nematodes,” Cell 152, no. 1 (2013): 109–119. 23332749 10.1016/j.cell.2012.12.013

[34] S.‐Y. Takemura , A. Bharioke , Z. Lu , et al., “A Visual Motion Detection Circuit Suggested by Drosophila Connectomics,” Nature 500, no. 7461 (2013): 175–181. 23925240 10.1038/nature12450 PMC3799980

[35] R. M. Lewis , H. Baskaran , J. Green , et al., “3D Visualization of Trans‐Syncytial Nanopores Provides a Pathway for Paracellular Diffusion Across the Human Placental Syncytiotrophoblast,” Iscience 25, no. 12 (2022): 105453. 36387021 10.1016/j.isci.2022.105453 PMC9663330

[36] S. Gaffling , V. Daum , S. Steidl , A. Maier , H. Köstler , and J. Hornegger , “A Gauss‐Seidel Iteration Scheme for Reference‐Free 3‐D Histological Image Reconstruction,” IEEE Transactions on Medical Imaging 34, no. 2 (2014): 514–530. 25312918 10.1109/TMI.2014.2361784 PMC4418037

[37] G. Mahalingam , R. Torres , D. Kapner , et al., “A Scalable and Modular Automated Pipeline for Stitching of Large Electron Microscopy Datasets,” Elife 11 (2022): e76534. 35880860 10.7554/eLife.76534 PMC9427110

[38] C. Lu , K. Chen , H. Qiu , et al., “Diffusion‐Based Deep Learning Method for Augmenting Ultrastructural Imaging and Volume Electron Microscopy,” Nature Communications 15, no. 1 (2024): 4677. 10.1038/s41467-024-49125-z PMC11144272 38824146

[39] J. He , Y. Zhang , W. Sun , G. Yang , and F. Sun , “IsoVEM: Isotropic Reconstruction for Volume Electron Microscopy Based on Transformer,” BioRxiv (2023): 2023–11.

[40] I. Belevich , M. Joensuu , D. Kumar , H. Vihinen , and E. Jokitalo , “Microscopy Image Browser: A Platform for Segmentation and Analysis of Multidimensional Datasets,” PLoS Biology 14, no. 1 (2016): e1002340. 26727152 10.1371/journal.pbio.1002340 PMC4699692

[41] S. Suganyadevi , V. Seethalakshmi , and K. Balasamy , “A Review on Deep Learning in Medical Image Analysis,” International Journal of Multimedia Information Retrieval 11, no. 1 (2022): 19–38. 34513553 10.1007/s13735-021-00218-1 PMC8417661

[42] M. Pu , Y. Huang , Q. Guan , and H. Ling , “RINDNet: Edge Detection for Discontinuity in Reflectance, Illumination, Normal and Depth,” in Proceedings of the IEEE/CVF International Conference on Computer Vision (2021): 6879–6888.

[43] H. Abdi , “Singular Value Decomposition (SVD) and Generalized Singular Value Decomposition,” Encyclopedia of Measurement and Statistics 907, no. 912 (2007): 44.

[44] Z. Zhang , H. Li , Z. Xu , et al., A Gaussian filter-based 3D registration method for series section electron microscopy[C]//Proceedings of the AAAI Conference on Artificial Intelligence 39, no. 1 (2025): 1156–1164.

[45] S. Paris , P. Kornprobst , J. Tumblin , et al., Bilateral filtering: Theory and applications[J]. Foundations and trends in computer graphics and vision 4, no. 1 (2009): 1–73.

[46] V. B. Sebastian , A. Unnikrishnan , and K. Balakrishnan , Gray level co-occurrence matrices: generalisation and some new features[J]. arXiv preprint arXiv:1205.4831, (2012).

