# Supplementary material for: vEMRec: High‐Resolution Volume Electron Microscopy Reconstruction Based on Structure‐Preserving and High‐Fidelity 3D Alignment
Source: Adv Sci (Weinh). 2026 Feb 20;13(23):e19098. doi: 10.1002/advs.202519098 (PMC13104135; doi:10.1002/advs.202519098)
Supplement: Supplementary file 1 — Supporting File 1: advs74367‐sup‐0001‐SuppMat.pdf. [file ADVS-13-e19098-s002.pdf]

# Supplementary Materials for "vEMRec: High-Resolution Volume Electron Microscopy Reconstruction Based on Structure-Preserving and High-Fidelity 3D Alignment"

## S1 Data preparation

### S1.1 Details of datastes

We selected six publicly available datasets from the OpenOrganelle platform [16] for our simulations. These datasets consist of electron microscope images of various mouse tissues, including heart, kidney, liver, skin, and pancreas. The availability of these high-resolution images as ground truth enables a comprehensive evaluation of our method's applicability and robustness across different biological tissues. The images were downsampled and cropped to a size of $1184 \times 1184$ pixels. For real-world data, , we selected six datasets, including the FAFB (Full Adult Fly Brain) dataset [17] with image sizes of $8192 \times 8192$ pixels, the Caenorhabditis elegans cell dataset [2] with image sizes of $21504 \times 26624$ pixels, the mouse cortical dataset [6] with image sizes of $24576 \times 20480$ pixels, and the female fruit fly brain neural dataset [13] with image sizes of $8192 \times 8192$ pixels. Moreover, we selected two datasets of human term placental villi, with each image sized at $3000 \times 3000$ pixels. Detailed information on all datasets used in our experiments is provided in Supplementary Table S1 and Table S2. These datasets include both neuronal and organellar data, and the differences in their slice-level ultrastructural patterns highlight this diversity. Representative examples of these distinct structural characteristics are shown in Figure S1. The simulation detailed can be found in Section S1.2.

Table S1: Details of the data used in the experiment

| Datasets | image shape | slices numbers | url |
|---|---|---|---|
| jrc_mus-heart-1-s4 | 1184×1184 | 1000 | https://openorganelle.janelia.org/datasets/jrc_mus-heart-1 |
| jrc_mus-kidney-s4 | 1184×1184 | 1000 | https://openorganelle.janelia.org/datasets/jrc_mus-kidney |
| jrc_mus-liver-3-s4 | 1184×1184 | 1127 | https://openorganelle.janelia.org/datasets/jrc_mus-liver-3 |
| jrc_mus-liver-s4 | 1184×1184 | 558 | https://openorganelle.janelia.org/datasets/jrc_mus-liver |
| jrc_mus-pancreas-4-s4 | 1184×1184 | 898 | https://openorganelle.janelia.org/datasets/jrc_mus-pancreas-4 |
| jrc_mus-skin-1-s4 | 1184×1184 | 1231 | https://openorganelle.janelia.org/datasets/jrc_mus-skin-1 |
| FAFB | 8192×8192 | 76100 | https://temca2data.org/ |
| mouse cortex | 24576×20480 | 2762 | https://neurodata.io/data/bumbarger13/ |
| C.elegans | 21504×26624 | 1850 | https://neurodata.io/data/kasthuri15/ |
| FlyFemBrain | 8192x8192 | 700 | https://www.ebi.ac.uk/empiar/EMPIAR-10967/ |
| Human term placental villi(Stack 12) | 3000×3000 | 366 | https://www.ebi.ac.uk/empiar/EMPIAR-10967/ |
| Human term placental villi(Stack 13) | 3000×3000 | 300 | https://www.ebi.ac.uk/empiar/EMPIAR-10967/ |
| Human term placental villi(Stack 39) | 3000×3000 | 357 | https://www.ebi.ac.uk/empiar/EMPIAR-10967/ |

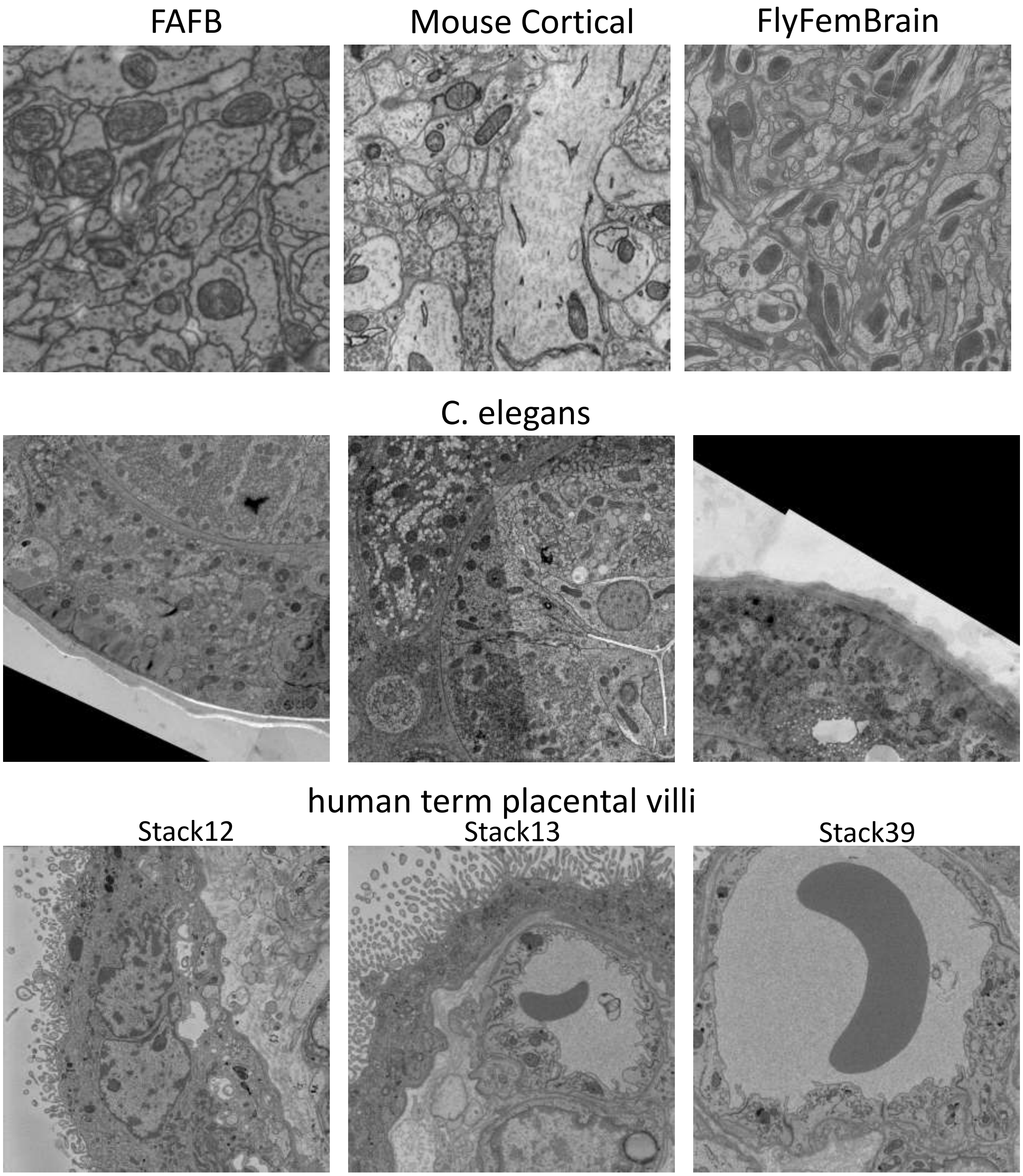


Figure S1: Representative examples of neuronal and non-neuronal vEM datasets used in this study, illustrating the structural differences across diverse biological samples.

Table S2: Summary of datasets and corresponding volume electron microscopy (vEM) modalities used in this study.

| Dataset | Organism / Tissue | Imaging Modality | Reference |
|---|---|---|---|
| FAFB (Full Adult Fly Brain) | Drosophila melanogaster brain | ssTEM (TEMCA system) | [17] |
| C. elegans cell dataset | Caenorhabditis elegans | TEM | [2] |
| Mouse cortical dataset | Mouse neocortex | ATUM-SEM | [6] |
| Female fruit fly brain neural dataset | Drosophila melanogaster brain | TEM | [13] |
| Human term placental villi dataset | Human placenta (term villi) | SBF-SEM | [7] |
| OpenOrganelle simulated dataset | Six different mouse cell tissues | FIB-SEM | [4] |

## S1.2 Dataset simulation

**Dataset simulation for rigid alignment.** To generate datasets for the rigid alignment experiments, we applied random rigid translations and rotations to the six mouse tissue datasets from the OpenOrganelle platform. The random rigid transformation is represented as follows:

$$\begin{pmatrix} \mathbf{I}' \\ 1 \end{pmatrix} = \begin{pmatrix} \cos(\theta) & -\sin(\theta) & t_x \\ \sin(\theta) & \cos(\theta) & t_y \\ 0 & 0 & 1 \end{pmatrix} \begin{pmatrix} \mathbf{I} \\ 1 \end{pmatrix} \tag{1}$$

where $(t_x, t_y)$ indicates the random displacement, and $\theta$ represents the random rotation angle. We simulated the data using three different strategies: In Strategy 1, random translations were applied to the original image stack, with translation parameters $(t_x, t_y)$ randomly selected within the range $[0, 100]$. In Strategy 2, random rotations were applied to the original image stack to simulate sample motion, with translation parameters $(t_x, t_y)$ randomly selected within the range $[0, 100]$ and rotation angles $\theta$ randomly selected within $[-35°, 35°]$. In Strategy 3, combined random rigid transformations (both translation and rotation) were applied to the original image stack to simulate more complex rigid deformations, with translation parameters $(t_x, t_y)$ randomly selected within the range $[0, 100]$ and rotation angles $\theta$ randomly selected within $[-35°, 35°]$.

In the noise robustness experiment for rigid alignment, Gaussian noise of varying intensity was added to the image stack, as illustrated in Figure S2. The noise intensity was controlled by the parameter $\sigma$, with values set at $\sigma = 1.0, 1.5, 2.0, 2.5, 3.0, 3.5$. To more clearly illustrate how increasing Gaussian noise levels affect image quality, the

images were further examined in the Fourier domain (Figure S3). As more noise was injected, the high-frequency components increased noticeably. For each $\sigma$, the two-dimensional power spectral density (PSD) $P(u, v) = |F(u, v)|^2$ was computed, followed by radial averaging to obtain $P(r)$, which was then normalized by the low-frequency mean. The relative PSD was plotted as a function of the normalized frequency radius $r$ using $\mathrm{PSD}_{\mathrm{dB}}(r) = 10 \log_{10}\left(P(r)/P_{\text{low-freq mean}}\right)$. As the Gaussian noise level increased, the high-frequency components (for $r > 0.3$) rose markedly and the overall PSD slope flattened, from the natural-image $1/f^{\alpha}$ profile toward a flat white-noise spectrum. This frequency-domain analysis provides quantitative evidence that stronger noise injects additional high-frequency energy, thereby degrading structural coherence.

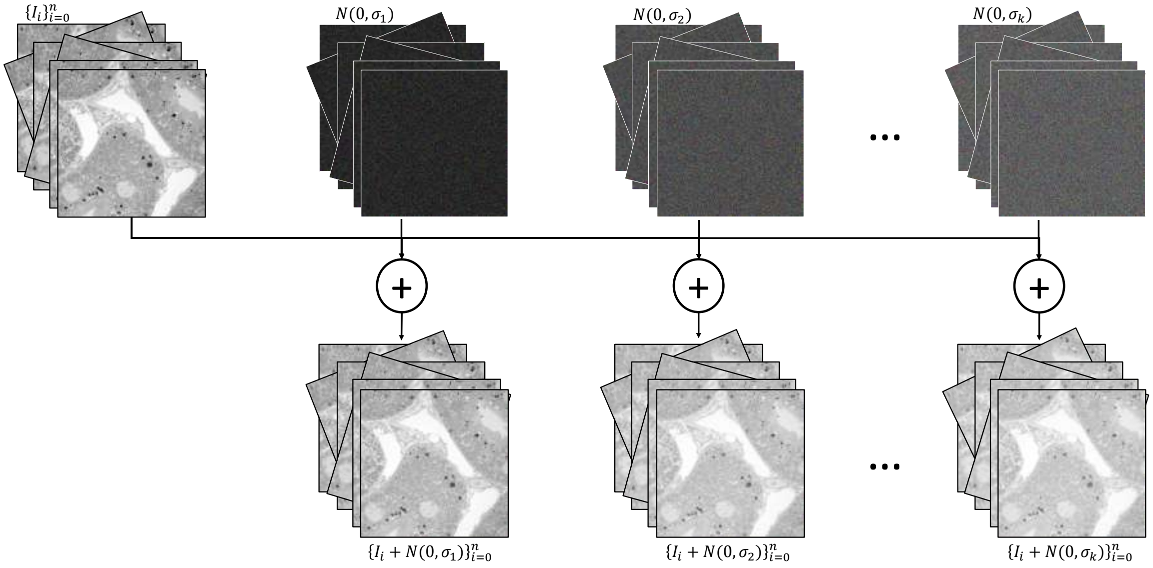


Figure S2: In the noise robustness test of the rigid alignment experiment, different levels of Gaussian noise were added to the entire image stack by adjusting the parameters of the Gaussian noise.

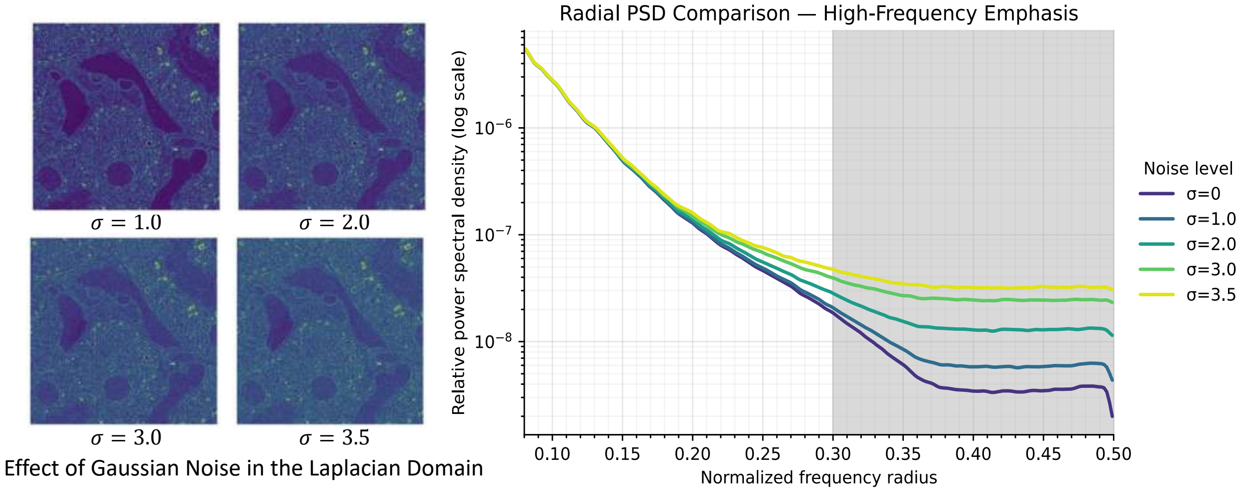


Figure S3: Image degradation under increasing Gaussian noise levels $\sigma$. **Left:** Laplacian-based high-frequency residual energy maps, where stronger responses indicate higher noise levels. **Right:** Relative power spectral density (PSD) plotted against the normalized frequency radius $r$, showing that increasing noise flattens the PSD slope from the natural-image $1/f^{\alpha}$ profile toward a white-noise spectrum.

**Dataset simulation for elastic registration.** To generate datasets for the elastic registration experiments, we applied random elastic deformations[12] to the six mouse tissue datasets from the OpenOrganelle platform by introducing simulated nonlinear distortions to the image stack, as depicted in Figure S4. This was achieved by adding a Gaussian-smoothed random displacement field to the images. Specifically, we firstly generate a random deformation field $D_{\text{rand}}$ which indicates the pixel displacement matrix and then utilize the Gaussian filter to smooth this matrix, which was then applied to create the deformed images. The deformation formula is expressed as follows:

$$\begin{cases} \varphi_i = & \alpha \cdot \text{size}(I_i) \cdot \text{Gauss}\left(D_{\text{rand}}, \sigma \cdot \text{size}(I_i)\right), \\ \hat{I}_i = & \varphi_i \circ I_i, \end{cases} \tag{2}$$

where $\varphi_i$ represents the random deformation field of image $I_i$, $\circ$ denotes the deformation applied to the image, $D_{\text{rand}}$ is the generated random displacement field, $Gauss(\cdot)$ is a 2D Gaussian filter operator, $\alpha$ controls the displacement magnitude, and $\sigma$ controls the smoothing extent. In our experiments, we fixed $\sigma = 0.08$ and varied $\alpha$ as $1.0, 1.5, 2.0, 2.5, 3.0$.

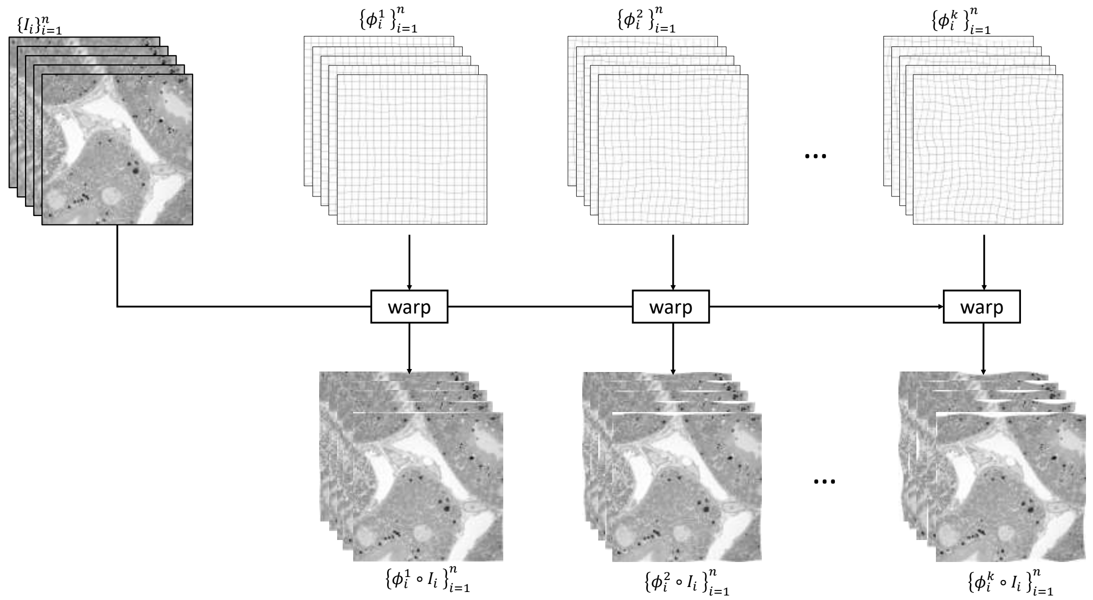


Figure S4: In the robustness test of elastic deformation in the elastic registration experiment, different levels of elastic deformation were applied to the entire image stack by adjusting the parameters of the elastic deformation.

# S2 Supplementary demonstrations of vEMRec performance

## S2.1 More analysis of 3D rigid alignment module

To comprehensively evaluate the performance of our rigid alignment method, we conducted a comparison with two widely used 3D rigid alignment tools: StackReg and TrakEM2. To simulate random mechanical displacements and rotations, we applied random rigid translations and rotations to different datasets. Specifically, for each dataset, we performed three simulation strategies: In Strategy 1, random translations were applied to the original image stack. In Strategy 2, random rotations were introduced to simulate rigid motion. In Strategy 3, both random translations and rotations were applied to simulate complex rigid deformations. The performance of each alignment algorithm was evaluated by comparing the normalized NCC, SSIM, and MI between the aligned image stacks and the ground truth, providing a comprehensive measure of alignment accuracy. For clarity, the mapping between evaluation tasks and the datasets used in each experiment is summarized in Table S11.

The complete quantitative results for all datasets in the rigid alignment stage are provided in the Supplementary Materials (Table S4), which compares the alignment accuracy of vEMRec, StackReg, and TrakEM2 across three scenarios involving different degrees of rigid rotation and translation, evaluated on three representative datasets.

In the simplest case (Scenario 1, involving only translation), StackReg achieved the highest accuracy, outperforming both vEMRec and TrakEM2. This result can be attributed to StackReg's gray-level template matching, which is particularly effective for simple translations and aligns well with intensity-based optimization. However, such translation-only cases are rare in real-world vEM acquisition pipelines, where slice misalignments are typically accompanied by rotations, nonlinear distortions, and cumulative drift. For this reason, our experimental design emphasizes more complex rigid transformations that better reflect practical scenarios. vEMRec is specifically designed to handle large and complex rigid misalignments, including substantial rotations and translations, and demonstrates clear advantages in these settings. In Scenario 2 (random rotations), vEMRec achieved the highest SSIM on most datasets, 0.78 on the mouse heart and 0.83 on the mouse liver, followed by TrakEM2, which obtained 0.76 and 0.81, respectively. This advantage arises because both vEMRec and TrakEM2 employ local feature–based, scale- and rotation-invariant algorithms, demonstrating strong robustness to rotational misalignment. On the mouse kidney dataset, TrakEM2 slightly outperformed vEMRec, with both achieving approximately 0.86 SSIM. In contrast, StackReg performed poorly, with

SSIM scores of 0.74 (heart), 0.81 (kidney), and 0.75 (liver), as large rotations disrupt its template matching and cause frequent misalignment failures. In the most complex case (Scenario 3, combining rotation and translation), vEMRec consistently outperformed all methods across every dataset and metric, confirming its accuracy and robustness under complex transformations.

Additional rigid alignment experiments in Figure 2 of the main text, primarily referring to the error accumulation, slice interval, and Gaussian noise level analyses shown in Figure 2d, are supported by complete quantitative results presented in Figures S5–S10 of the Supplementary Materials. These supplementary figures provide a detailed comparison of vEMRec, StackReg, and TrakEM2 under different experimental conditions.

**(a) Error Accumulation (Figures S5–S6).** All methods exhibit a gradual decline in registration accuracy as the number of slices increases, indicating that rigid alignment alone cannot fully eliminate cumulative errors and that subsequent elastic registration is essential. Nevertheless, vEMRec demonstrates overall superior performance, showing clear advantages on the mouse kidney dataset in Figure S5 and on the mouse heart and liver datasets in Figure S6, where it more effectively mitigates error accumulation. For the mouse liver and pancreas datasets in Figure S5 and the mouse skin dataset in Figure S6, vEMRec and TrakEM2 perform comparably, both significantly outperforming StackReg.

**(b) Robustness to High Noise (Figures S7–S8).** Under high-noise conditions, as shown in Figures S7–S8, vEMRec exhibits stronger noise resistance, clearly outperforming TrakEM2 on the mouse kidney dataset in Figure S7 and on the mouse liver, skin, and heart datasets in Figure S8. For the mouse liver and pancreas datasets in Figure S7, the two methods perform similarly, with TrakEM2 slightly higher in a few metrics.

**(c) Robustness to Slice Deformation (Figures S9–S10).** When the slice interval increases, indicating larger inter-slice deformation, vEMRec maintains stronger robustness to deformation. As illustrated in Figures S9–S10, vEMRec outperforms TrakEM2 on the mouse liver dataset in Figure S9 and on the mouse heart, liver, and skin datasets in Figure S10. For the mouse kidney and pancreas datasets in Figure S9, some vEMRec metrics are marginally lower than those of TrakEM2.

Overall, across all six datasets, vEMRec achieves higher or comparable alignment accuracy relative to TrakEM2 and StackReg. The extensive experiments summarized in Figures S5–S10 demonstrate that vEMRec provides superior performance in mitigating error accumulation, resisting high noise, and maintaining robustness against inter-slice

deformation, thereby confirming its reliability and generalization capability across diverse vEM imaging conditions.

### S2.2 More analysis of 3D elastic registration module

To demonstrate the robustness of vEMRec across varied cellular scenarios and complex conditions, we assessed its performance on six simulated datasets representing mouse liver, pancreas, skin, heart, and kidney tissues, evaluating both 3D rigid alignment and elastic registration. Using ground truth (GT) data, we calculated the Normalized Cross-Correlation (NCC), Mutual Information (MI), and Structural Similarity Index (SSIM) between the registered image series and GT, providing quantitative metrics for accuracy and reliability. Dice coefficient and Hausdorff Distance between the segmented results and the ground truth labels are calculated to evaluate the segmentaion precision.

For elastic registration, we compared our method, vEMRec, with two algorithms: SEAMLeSS and TrakEM2. Figures S11 and S12 highlight the performance of each elastic registration algorithm in mitigating error accumulation, while Figures S13 and S14 show accuracy in scenarios with complex nonlinear distortions. Our data reveal that vEMRec consistently outperforms other methods across all datasets, achieving accuracy improvements of 10% to 20% and surpassing 20% for SSIM, indicating robust handling of complex nonlinear distortions across different tissue types. To emphasize the impact of our method on downstream tasks, such as 3D segmentation, Figures S15 and S16 compare 3D segmentation precision (Dice score and Hausdorff Distance) with registered image stacks across various methods. Our method's results closely align with ground truth, accurately recovering 3D structures vital for precise segmentation. Additionally, Figures S17, S18, S19, and S20 provide 3D visualizations of the registration results and subsequent segmentation. Compared to other methods, our approach yields visualizations that most closely approximate the ground truth, demonstrating its effectiveness in restoring accurate 3D structures of biological samples.

To evaluate the effectiveness of the Gaussian filtering strategy in our core algorithm, we report the registration accuracy with and without this strategy in Table S5. To further demonstrate its generality and adaptability, we replaced the original optical flow estimation network with another representative model. Specifically, we selected LKUnet [5] as the registration module within the Gaussian filtering framework and retrained it following our training protocol. As shown in Table S5, without the Gaussian filtering strategy, the network functions merely as a basic registration module, leading to a notable drop in accuracy. However, after incorporating the Gaussian filtering strategy, the registration performance is significantly improved. These results validate both the effectiveness of the core strategy and its strong

compatibility and generalizability when integrated with advanced network architectures.

### S2.3 Visualization results of the 3D elastic registration

Figures S21 and S22 display side views of the raw datasets, SEAMLeSS-registered datasets, and vEMRec-registered datasets for three real datasets: mouse cortex, FAFB, and C. elegans. Although ground truth is unavailable for these real datasets, we can found from the y-z side views that vEMRec clearly restores axial structural connectivity, with no visible breakpoints or jagged edges.

### S2.4 Validation of the proposed multi-scale difference filtering evaluation strategy

To tackle the challenge of evaluating registration performance on real datasets without ground truth, we developed a multi-scale difference filtering evaluation strategy. This approach balances noise reduction and biological structure preservation, providing a comprehensive assessment of 3D registration performance. To validate our method, we tested it on six simulated datasets with ground truth as a reference. Figures S24 and S25 present evaluation results across different conditions: noisy data, registered outputs from SEAMLeSS, results from our method, vEMRec, and the ground truth. The figures illustrate that, in terms of noise reduction, the ground truth achieves the lowest entropy and highest signal-to-noise ratio (SNR), followed by vEMRec, SEAMLeSS, and finally, the noisy data. For preserving biological structure information, the ground truth again shows the highest contrast across all scales, with vEMRec closely trailing. SEAMLeSS and noisy data perform similarly in this regard, aligning with quantitative evaluation results from other metrics (MI, NCC, and SSIM) in our previous experiments. Overall, these results confirm that our evaluation strategy operates as intended, validating the accuracy and robustness of the multi-scale difference filtering approach.

### S2.5 Analysis of trakEM2 parameter settings and computational efficiency of vEMRec

As shown in Figure 4(a) of the main text, TrakEM2 achieves the shortest runtime but substantially lower registration accuracy. In the main text, TrakEM2 was executed with its default parameter configuration. As described in the Section S4, "Elastic registration baseline implementation details"), the `scale` parameter was set to 0.25, corresponding to registration performed at one-quarter of the original image resolution. This setting was adopted as a trade-off to reduce runtime, as TrakEM2 is a conventional optimization-based algorithm that does not leverage GPU acceleration,

in contrast to vEMRec. While this configuration leads to faster runtime, it considerably compromises alignment precision.

To further analyze this trade-off, we additionally evaluated TrakEM2 with `scale` values of 0.5 and 1.0. As summarized in Tables S7 and S8, increasing the scale factor improves accuracy but results in a dramatic increase in computation time, exceeding that of vEMRec, while still failing to achieve comparable registration accuracy. These results demonstrate that vEMRec consistently attains superior alignment performance across all resolutions while maintaining lower computational cost, thereby confirming its enhanced computational efficiency.

### S2.6 Ablation study on the necessity of the rigid alignment stage

The rigid alignment stage in vEMRec is designed to remove large-scale global misalignments, primarily inter-slice translations and rotations, thereby providing a well-initialized input for the subsequent elastic registration. Without this stage, the elastic module would need to simultaneously correct both global and local distortions, which significantly increases registration difficulty and may lead to convergence failures. To quantitatively assess the necessity of the rigid alignment stage, we conducted ablation experiments comparing performance with and without it under different levels of simulated distortion. The results are summarized in Table S9 and Table S10.

As shown in Table S9, for challenging cases where slices were perturbed by large global transformations (approximately 35° rotation and 100-pixel translation), the default configuration used in the subsection *"Comprehensive validation of the rigid alignment and elastic registration modules in vEMRec"*, removing the rigid alignment stage caused the elastic registration to fail entirely, producing severely distorted or misaligned reconstructions. In contrast, when the rigid alignment step was included, vEMRec successfully recovered accurate structural correspondence and achieved high-fidelity 3D continuity. As presented in Table S10, we further evaluated cases with milder perturbations (e.g., 5° rotation and 10-pixel translation). In these settings, the elastic registration module alone could still operate but yielded reduced alignment accuracy, confirming that rigid alignment remains beneficial even under small global shifts. These results collectively demonstrate that the rigid alignment stage is indispensable for achieving robust and precise alignment, particularly in datasets with substantial initial displacements.

For real-world datasets, the data already exhibit approximate coarse alignment but still contain noticeable non-linear distortions caused by sectioning and imaging artifacts. In such cases, we apply only the elastic registration

stage to refine alignment, enhance axial continuity, and restore consistent 3D ultrastructural morphology. Overall, these experiments confirm that the rigid alignment module effectively corrects large global misalignments, while the elastic registration stage focuses on fine-scale nonlinear distortions, jointly enabling precise and structure-preserving volumetric reconstruction.

### S2.7 Comparison with additional baseline methods

In the main text, we selected TrakEM2 [11] and SEAMLeSS [8] as our primary baselines because (1) both methods have peer-reviewed publications that clearly describe their algorithmic principles, and (2) they have been widely adopted in large-scale EM reconstruction pipelines [1, 17]. These characteristics make them well-established representatives of classical optimization-based and modern deep-learning-based elastic registration frameworks, respectively.

Of course, additional high-quality 3D alignment toolkits exist, such as SOFIMA(https://github.com/google-research/sofima) and FEABAS(https://github.com/YuelongWu/feabas), both of which support elastic alignment. In this subsection, we treat these toolkits as relevant supplementary baselines and examine them from two complementary perspectives: a comparison of their underlying algorithmic principles with those of vEMRec, and a direct comparison of their alignment performance on real-world datasets.

**Comparison of methodological principles.** First, at the methodological level, we analyzed the publicly available source code of SOFIMA and FEABAS to understand their algorithmic designs. SOFIMA sequentially estimates flow fields between adjacent slice pairs, computing a displacement field using masked cross-correlation for each pair. This follows the same core idea as sequential alignment methods such as SEAMLeSS. Sequential slice-to-slice alignment tends to remove not only nonlinear distortions but also natural inter-slice morphological variations, suppressing low-frequency biological changes and leading to cumulative alignment errors. This stands in contrast to the frequency-decoupling strategy in vEMRec, which preserves natural low-frequency deformations while removing high-frequency distortions, thereby avoiding such accumulation.

FEABAS performs elastic alignment of ssEM data by constructing a deformable finite-element mesh for each slice and solving a global minimum-energy problem across the full stack. High-resolution matching points extracted from the images serve as constraints, and the final deformation field balances an elastic smoothness term with a matching fidelity term. As a classical non–deep-learning optimization method, FEABAS is conceptually similar to TrakEM2.

It requires extensive parameter tuning, such as mesh resolution, iteration count, and smoothing hyperparameters, and can suffer from matching errors and convergence instability on challenging datasets.

**Comparison of alignment results on real-world data.** Second, at the empirical level, we incorporated SOFIMA and FEABAS into our benchmarking on real-world datasets and compared their performance directly with that of vEMRec. As shown in Figures S36 and S37, we provide side-view visualizations for SOFIMA, FEABAS, and vEMRec on the mouse cortical dataset and the Caenorhabditis elegans cell dataset. From these visualizations, SOFIMA produces smoother and sharper cell boundaries than the original data, indicating that it successfully removes nonlinear distortions. However, the aligned volumes also exhibit substantial structural alterations, including boundary voids and misalignment of cells. These artifacts are consistent with the limitations of sequential alignment: in addition to removing distortions, such methods often suppress natural biological variation and distort genuine low-frequency structural patterns, leading to cumulative alignment errors and biologically implausible morphology.

By contrast, FEABAS often produces alignment results that remain visually similar to the original data. According to the FEABAS documentation, the method relies heavily on reliable matching points between adjacent slices to drive deformation through a finite-element optimization framework. When image quality is limited or structural correspondences are ambiguous, insufficient matching points can be detected, leading to weak estimated deformation fields. In such cases, the alignment result differs only marginally from the original stack unless extensive and carefully tuned hyperparameter adjustments are performed. This behavior is consistent with official documentation regarding the sensitivity of FEABAS to hyperparameter choices and matching quality. Importantly, visual similarity to the original data should not be interpreted as successful correction of nonlinear distortions; rather, it often indicates that the method is unable to apply sufficiently strong corrective deformation under challenging conditions.

In comparison, vEMRec, built upon the proposed frequency-decoupling paradigm, successfully removes high-frequency nonlinear distortions while preserving low-frequency natural deformations that reflect true biological structure. It achieves robust and anatomically faithful 3D alignment without complex parameter adjustments and avoids the structural artifacts observed in sequential alignment methods. These additional experiments provide a comprehensive evaluation across distinct algorithmic paradigms and further demonstrate the advantages of vEMRec in accuracy, robustness, and structural preservation.

### S2.8 Discussion on multi-GPU parallelization

vEMRec is inherently designed to be parallel over slices, spatial tiles, and batches. Both the rigid alignment stage and the elastic registration stage operate on independent image units without cross-batch dependencies, making the pipeline naturally amenable to data-parallel execution across multiple GPUs.

In Table 1 of the main text, we report single-GPU benchmarks to provide controlled and reproducible measurements of algorithmic efficiency. Importantly, our single-GPU multi-batch experiments already demonstrate strong throughput scaling as batch size increases, indicating that the computation exhibits stable memory-bound and compute-bound behavior without hidden serialization bottlenecks. This behavior suggests that the core operations of vEMRec do not impose algorithmic constraints that would limit scalability.

In practical multi-GPU deployments, vEMRec can be extended by distributing the image stack across devices. For example, when aligning a 3D volume consisting of 2000 slices, the workload can be partitioned across four GPUs by processing slices 1–500, 501–1000, 1001–1500, and 1501–2000 in parallel. Within each GPU, the same multi-batch processing strategy can be applied. Under this data-parallel execution model, near-linear throughput scaling is expected as the number of GPUs increases.

For clarity, we provide a comparison of the per-slice processing time under multi-GPU configurations for data with a resolution of 4096×4096. As shown in Table S3, following our stack-partitioning strategy, the alignment throughput exhibits near-linear scaling as the number of GPUs increases. The small deviations from ideal linear speedup can be attributed to system-specific factors, such as hardware configuration and I/O constraints.

Table S3: Per-slice alignment runtime scaling of vEMRec under ideal multi-GPU data-parallel execution, evaluated on 4096×4096 resolution data.

| Number of GPUs | Relative Throughput | Runtime (s/slice) | Speedup |
|---|---|---|---|
| 1 GPU | 1× | 32.44 | ~ 1.0× |
| 2 GPUs | 2× | 18.37 | ~ 2.0× |
| 4 GPUs | 4× | 11.69 | ~ 4.0× |

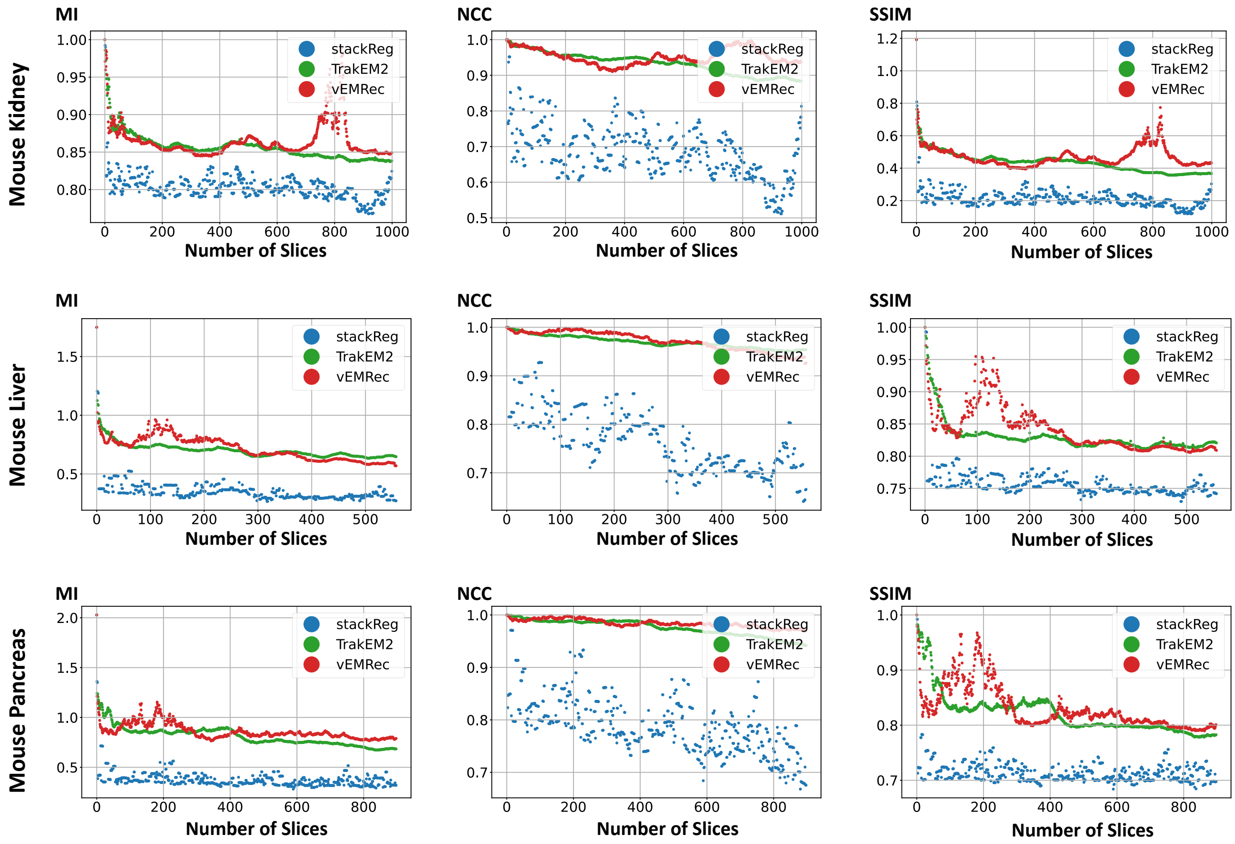


Figure S5: The variation in alignment accuracy of StackReg, TrakEM2, and vEMRec (rigid) on mouse kidney, mouse liver and mouse pancreas datasets as the number of slices increases.

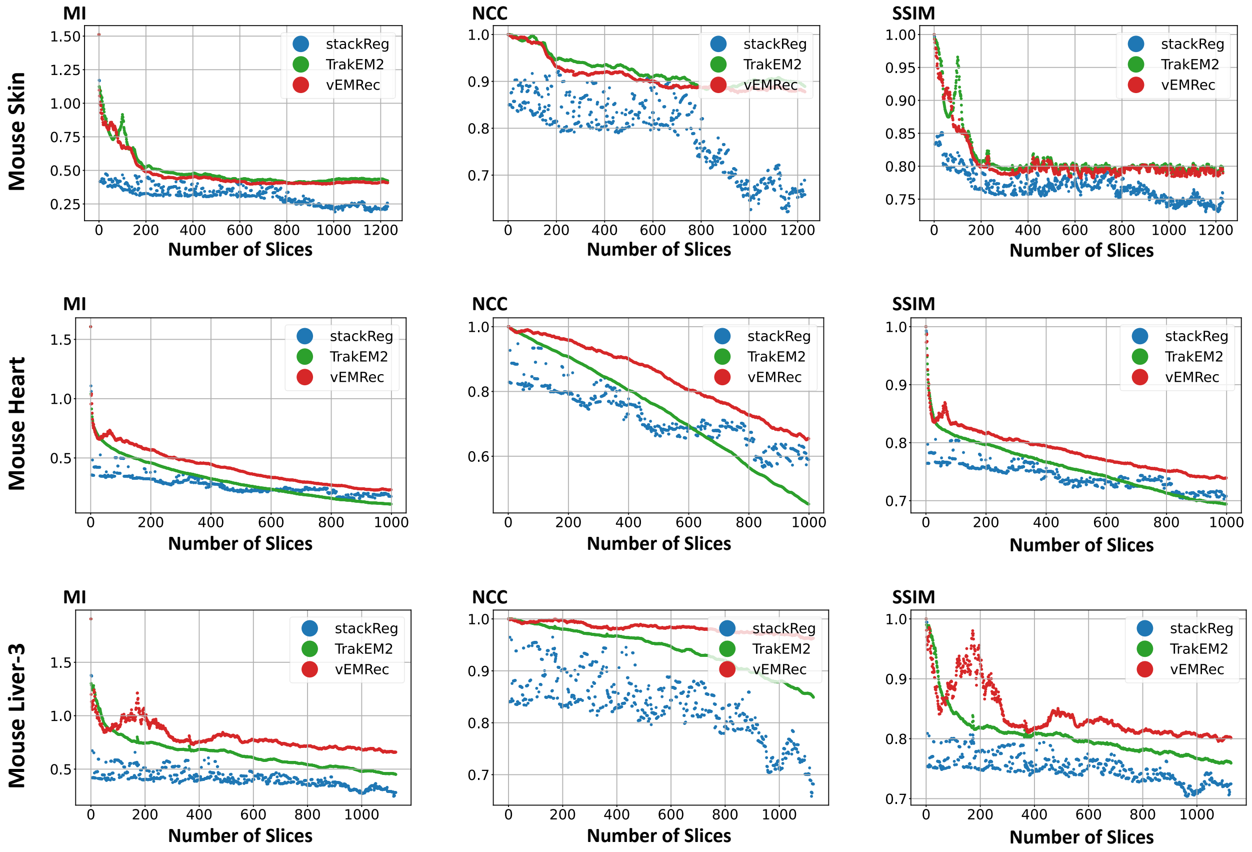


Figure S6: The variation in alignment accuracy of StackReg, TrakEM2, and vEMRec (rigid) on mouse skin, mouse heart and mouse liver-3 datasets as the number of slices increases.

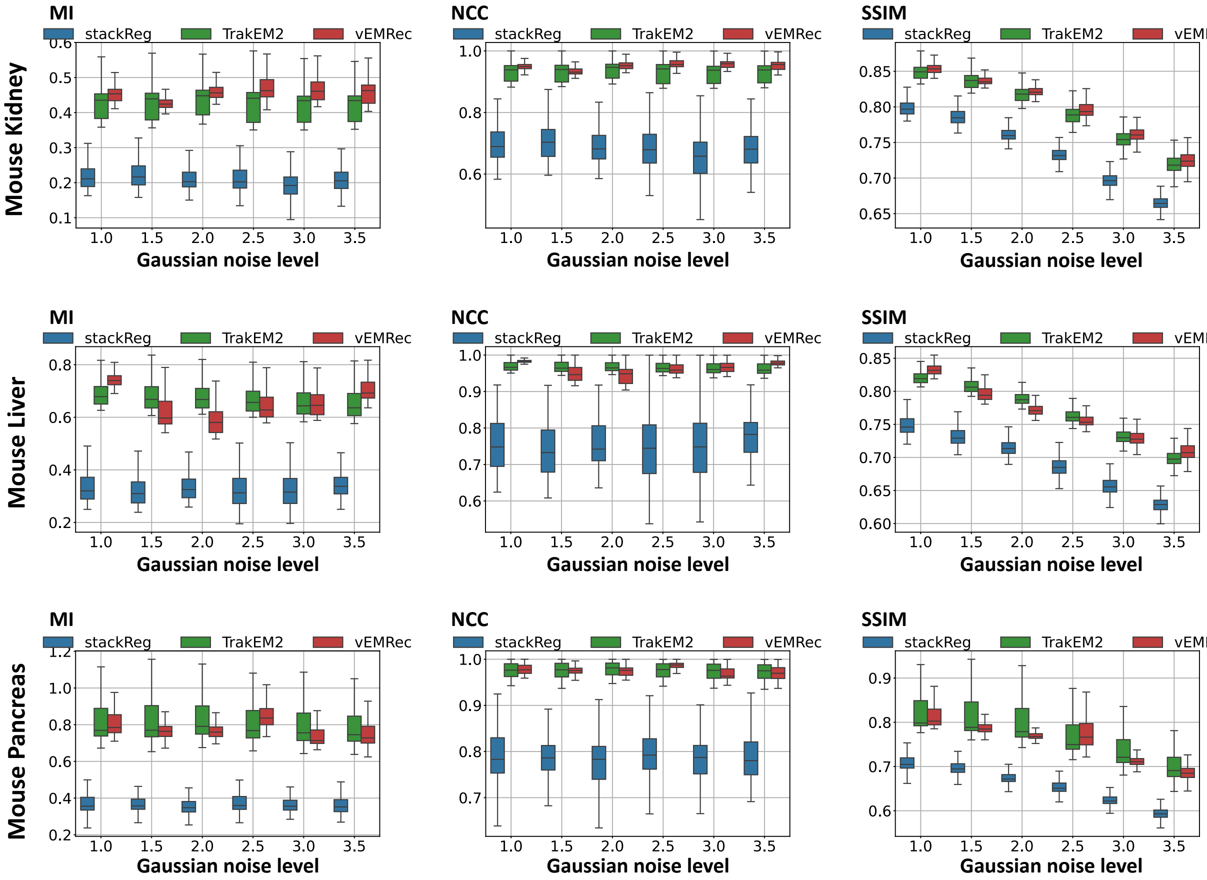


Figure S7: The comparison of alignment accuracy under different levels of Gaussian noise on mouse kidney, mouse liver and mouse pancreas datasets using StackReg, TrakEM2, and vEMRec (rigid).

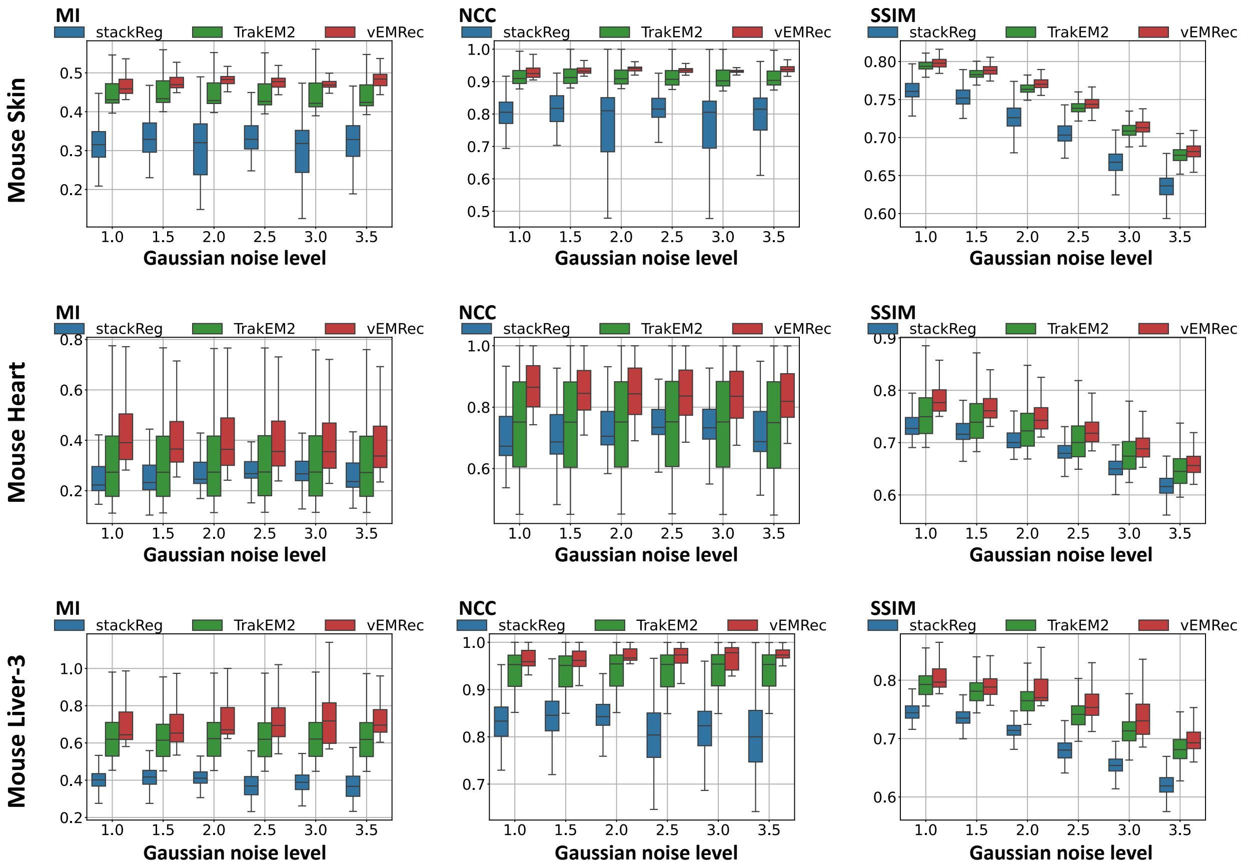


Figure S8: The comparison of alignment accuracy under different levels of Gaussian noise on mouse skin, mouse heart and mouse liver-3 datasets using StackReg, TrakEM2, and vEMRec (rigid).

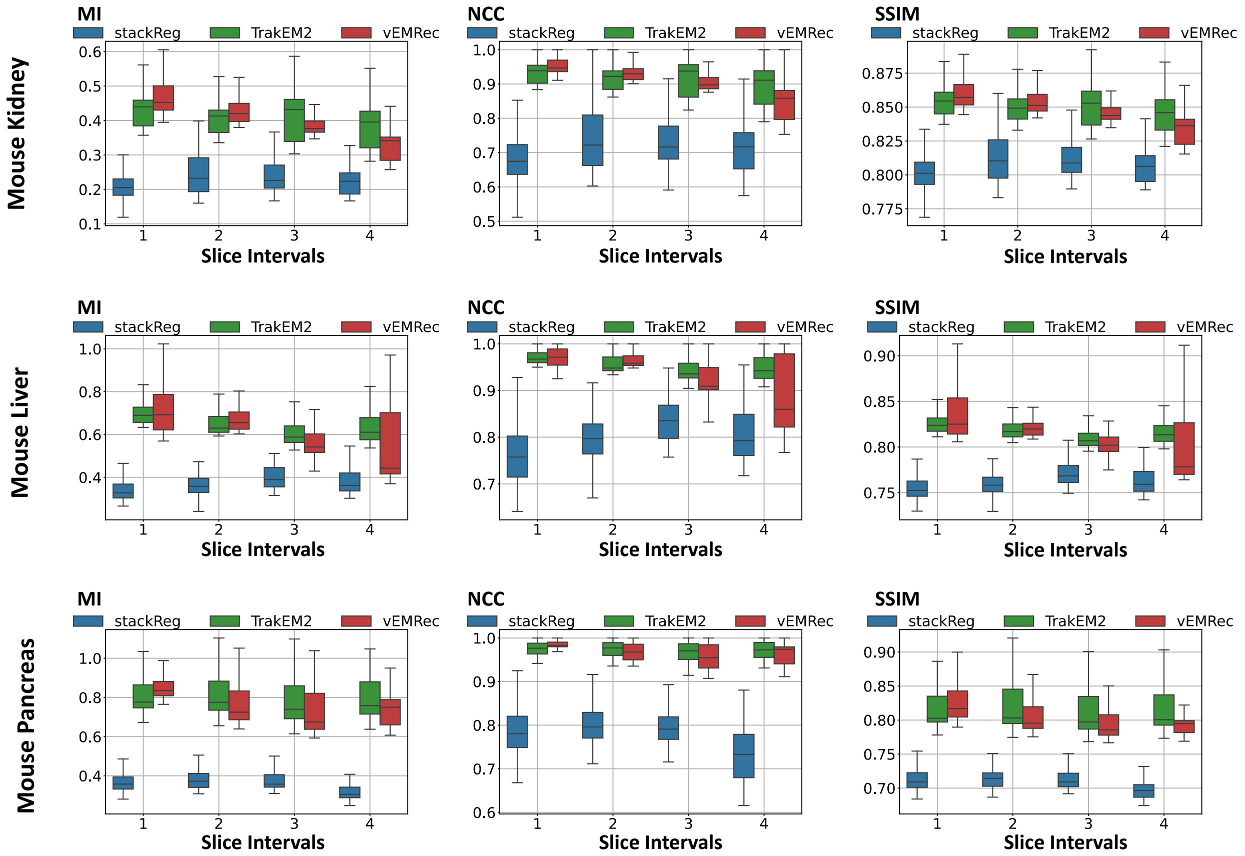


Figure S9: Comparison of alignment accuracy between StackReg, TrakEM2, and vEMRec (rigid) under different degrees of slice deformation (slice intervals) on mouse kidney, mouse liver and mouse pancreas datasets.

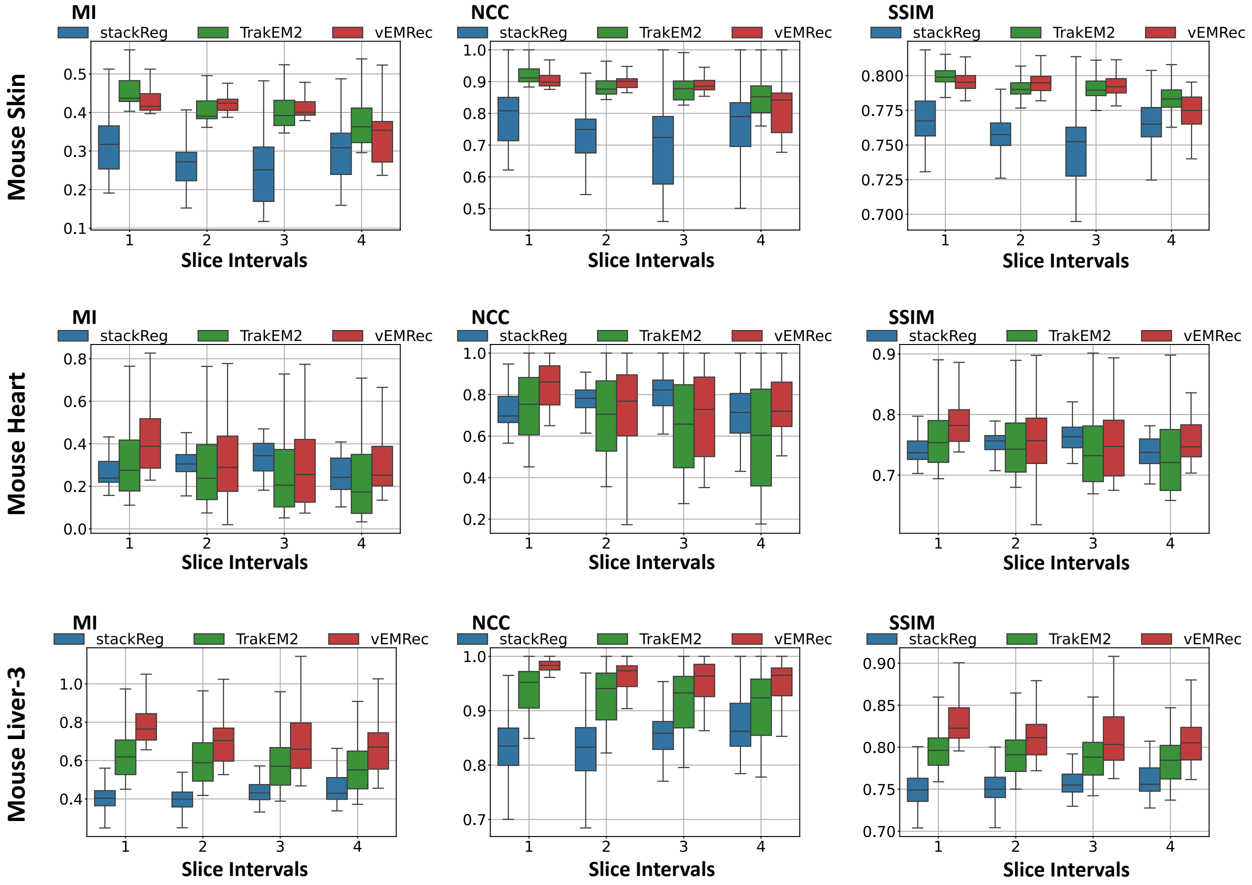


Figure S10: Comparison of alignment accuracy between StackReg, TrakEM2, and vEMRec (rigid) under different degrees of slice deformation (slice intervals) on mouse skin, mouse heart and mouse liver-3 datasets.

Table S4: Quantitative Comparison of 3D Rigid Alignment Results with Existing Methods.

| | | Experiment 1 | | | Experiment 2 | | | Experiment 3 | | |
|---|---|---|---|---|---|---|---|---|---|---|
| | | stackReg | TrakEM2 | vEMRec | stackReg | TrakEM2 | vEMRec | stackReg | TrakEM2 | vEMRec |
| jrc_mus-heart-1-s4 | NCC(GT) | **0.92** ± **0.05** | 0.75 ± 0.16 | 0.80 ± 0.13 | 0.71 ± 0.11 | 0.74 ± 0.16 | **0.82** ± **0.11** | 0.72 ± 0.08 | 0.74 ± 0.16 | **0.84** ± **0.11** |
| | SSIM(GT) | **0.80** ± **0.03** | 0.76 ± 0.04 | 0.77 ± 0.04 | 0.74 ± 0.03 | 0.76 ± 0.05 | **0.78** ± **0.03** | 0.74 ± 0.03 | 0.76 ± 0.05 | **0.79** ± **0.04** |
| | MI(GT) | **0.50** ± **0.12** | 0.32 ± 0.17 | 0.37 ± 0.16 | 0.26 ± 0.10 | 0.31 ± 0.17 | **0.38** ± **0.14** | 0.27 ± 0.09 | 0.31 ± 0.17 | **0.42** ± **0.16** |
| jrc_mus-kidney-s4 | NCC(GT) | **0.96** ± **0.02** | 0.94 ± 0.03 | 0.95 ± 0.02 | 0.72 ± 0.08 | **0.94** ± **0.02** | 0.94 ± 0.03 | 0.68 ± 0.07 | 0.93 ± 0.03 | **0.95** ± **0.02** |
| | SSIM(GT) | **0.86** ± **0.02** | 0.86 ± 0.02 | 0.86 ± 0.01 | 0.81 ± 0.02 | **0.86** ± **0.02** | 0.86 ± 0.02 | 0.80 ± 0.02 | 0.86 ± 0.02 | **0.86** ± **0.02** |
| | MI(GT) | **0.48** ± **0.05** | 0.44 ± 0.06 | 0.45 ± 0.04 | 0.23 ± 0.07 | **0.45** ± **0.06** | 0.44 ± 0.06 | 0.21 ± 0.06 | 0.44 ± 0.06 | **0.47** ± **0.06** |
| jrc_mus-liver-3-s4 | NCC(GT) | **0.97** ± **0.02** | 0.94 ± 0.04 | 0.97 ± 0.02 | 0.85 ± 0.05 | 0.94 ± 0.04 | **0.98** ± **0.01** | 0.83 ± 0.06 | 0.94 ± 0.04 | **0.98** ± **0.01** |
| | SSIM(GT) | 0.82 ± 0.04 | 0.81 ± 0.04 | **0.82** ± **0.03** | 0.75 ± 0.03 | 0.81 ± 0.04 | **0.83** ± **0.04** | 0.75 ± 0.03 | 0.80 ± 0.04 | **0.84** ± **0.04** |
| | MI(GT) | **0.72** ± **0.14** | 0.66 ± 0.16 | 0.72 ± 0.11 | 0.43 ± 0.11 | 0.66 ± 0.16 | **0.76** ± **0.11** | 0.41 ± 0.10 | 0.65 ± 0.15 | **0.79** ± **0.11** |

Table S5: Ablation study on the Gaussian filter strategy.

| | jrc_mus_kidney | jrc_mus_skin | jrc_mus_liver-3 |
|---|---|---|---|
| w/o Gauss filter | 0.63 | 0.67 | 0.66 |
| w/ Gauss filter | 0.78 | 0.82 | 0.80 |
| LKUnet | 0.43 | 0.47 | 0.41 |
| LKUnet+Gauss filter | 0.75 | 0.80 | 0.77 |

Table S6: Quantitative Comparison of 3D Elastic Registration Results with Existing Methods

| | | jrc_mus-heart-1-s4 | jrc_mus-kidney-s4 | jrc_mus-liver-3-s4 | jrc_mus-liver-s4 | jrc_mus-pancreas-4-s4 | jrc_mus-skin-1-s4 |
|---|---|---|---|---|---|---|---|
| TrakEM2 | MI(GT) | 1.01 ± 0.03 | 1.24 ± 0.10 | 1.48 ± 0.07 | 1.47 ± 0.08 | 1.48 ± 0.06 | 1.08 ± 0.15 |
| | SSIM(GT) | 0.57 ± 0.02 | 0.61 ± 0.03 | 0.66 ± 0.03 | 0.61 ± 0.03 | 0.65 ± 0.03 | 0.64 ± 0.06 |
| | NCC(GT) | 0.98 ± 0.00 | 0.97 ± 0.00 | 0.98 ± 0.00 | 0.97 ± 0.00 | 0.97 ± 0.00 | 0.98 ± 0.01 |
| SEAMLeSS | MI(GT) | 1.00 ± 0.12 | 1.07 ± 0.14 | 1.35 ± 0.17 | 1.29 ± 0.19 | 1.18 ± 0.15 | 0.89 ± 0.18 |
| | SSIM(GT) | 0.58 ± 0.04 | 0.52 ± 0.04 | 0.61 ± 0.05 | 0.52 ± 0.06 | 0.50 ± 0.05 | 0.51 ± 0.07 |
| | NCC(GT) | 0.97 ± 0.01 | 0.96 ± 0.01 | 0.97 ± 0.01 | 0.95 ± 0.01 | 0.95 ± 0.01 | 0.96 ± 0.01 |
| vEMRec | MI(GT) | **1.43 ± 0.05** | **1.54 ± 0.11** | **1.83 ± 0.07** | **1.83 ± 0.08** | **1.76 ± 0.06** | **1.40 ± 0.16** |
| | SSIM(GT) | **0.83 ± 0.02** | **0.78 ± 0.03** | **0.82 ± 0.02** | **0.80 ± 0.02** | **0.80 ± 0.02** | **0.82 ± 0.03** |
| | NCC(GT) | **0.99 ± 0.00** | **0.99 ± 0.00** | **0.99 ± 0.00** | **0.99 ± 0.00** | **0.99 ± 0.00** | **0.99 ± 0.00** |

Table S7: Average processing time (s) of TrakEM2 under different `scale` settings and image resolutions.

| **Scale** | **512×512** | **1024×1024** | **2048×2048** | **4096×4096** | **8192×8192** |
|---|---|---|---|---|---|
| 0.25 | 2.52 | 3.65 | 4.22 | 5.57 | 12.04 |
| 0.5 | 8.64 | 34.6 | 88.4 | 254.3 | 553.6 |
| 1.0 | 24.5 | 78.7 | 183.1 | 414.4 | 1857.2 |

Table S8: Registration accuracy (SSIM) of TrakEM2 under different `scale` settings and image resolutions.

| **Scale** | **512×512** | **1024×1024** | **2048×2048** | **4096×4096** | **8192×8192** |
|---|---|---|---|---|---|
| 0.25 | 0.56 | 0.57 | 0.54 | 0.34 | 0.35 |
| 0.5 | 0.61 | 0.62 | 0.61 | 0.47 | 0.49 |
| 1.0 | 0.68 | 0.66 | 0.62 | 0.56 | 0.55 |

Table S9: Quantitative comparison under approximately 35° rotation and 100-pixel translation.

| | | jrc_mus-heart-1-s4 | jrc_mus-kidney-s4 | jrc_mus-liver-3-s4 | jrc_mus-liver-s4 | jrc_mus-pancreas-4-s4 | jrc_mus-skin-1-s4 |
|---|---|---|---|---|---|---|---|
| only elastic registration | NCC(GT) | 0.45 | 0.52 | 0.49 | 0.47 | 0.39 | 0.41 |
| | SSIM(GT) | 0.31 | 0.36 | 0.29 | 0.33 | 0.32 | 0.27 |
| | MI(GT) | 0.71 | 0.74 | 0.73 | 0.79 | 0.78 | 0.72 |
| vEMRec | NCC(GT) | 0.99 | 0.99 | 0.99 | 0.99 | 0.99 | 0.99 |
| | SSIM(GT) | 0.82 | 0.77 | 0.8 | 0.82 | 0.82 | 0.81 |
| | MI(GT) | 1.41 | 1.52 | 1.79 | 1.81 | 1.77 | 1.41 |

Table S10: Quantitative comparison under approximately 5° rotation and 10-pixel translation.

| | | jrc_mus-heart-1-s4 | jrc_mus-kidney-s4 | jrc_mus-liver-3-s4 | jrc_mus-liver-s4 | jrc_mus-pancreas-4-s4 | jrc_mus-skin-1-s4 |
|---|---|---|---|---|---|---|---|
| | NCC(GT) | 0.82 | 0.86 | 0.83 | 0.85 | 0.85 | 0.84 |
| only elastic registration | SSIM(GT) | 0.73 | 0.71 | 0.72 | 0.75 | 0.73 | 0.71 |
| | MI(GT) | 0.99 | 1.14 | 1.32 | 1.35 | 1.31 | 0.98 |
| | NCC(GT) | 0.99 | 0.99 | 0.99 | 0.99 | 0.99 | 0.99 |
| vEMRec | SSIM(GT) | 0.84 | 0.79 | 0.82 | 0.83 | 0.83 | 0.81 |
| | MI(GT) | 1.43 | 1.55 | 1.82 | 1.84 | 1.79 | 1.42 |

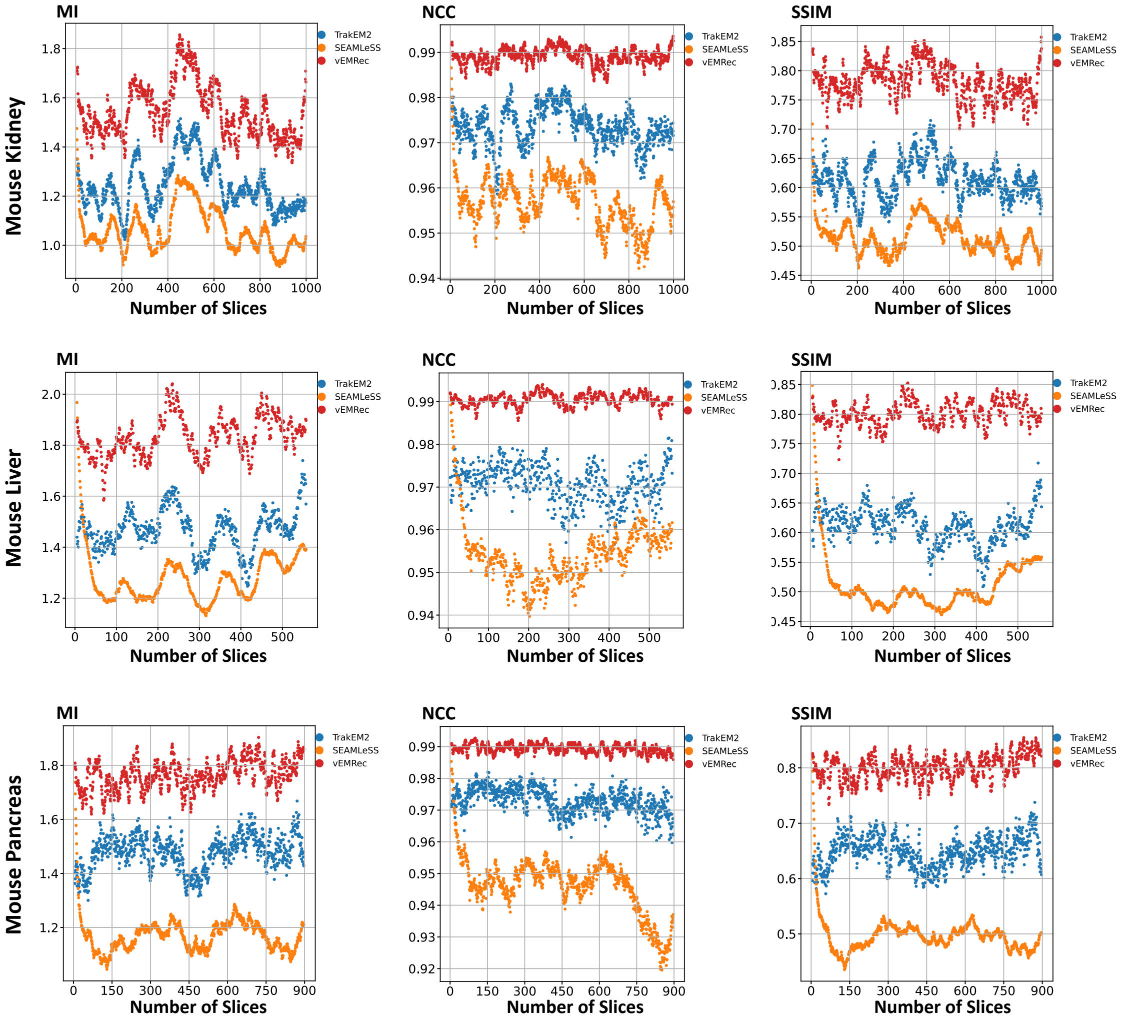


Figure S11: Comparison of MI(GT), NCC(GT), and SSIM(GT) metrics for various methods on the mouse kidney, mouse liver, and mouse pancreas datasets as the number of slices increases.(deformation levels $\alpha$=1.0)

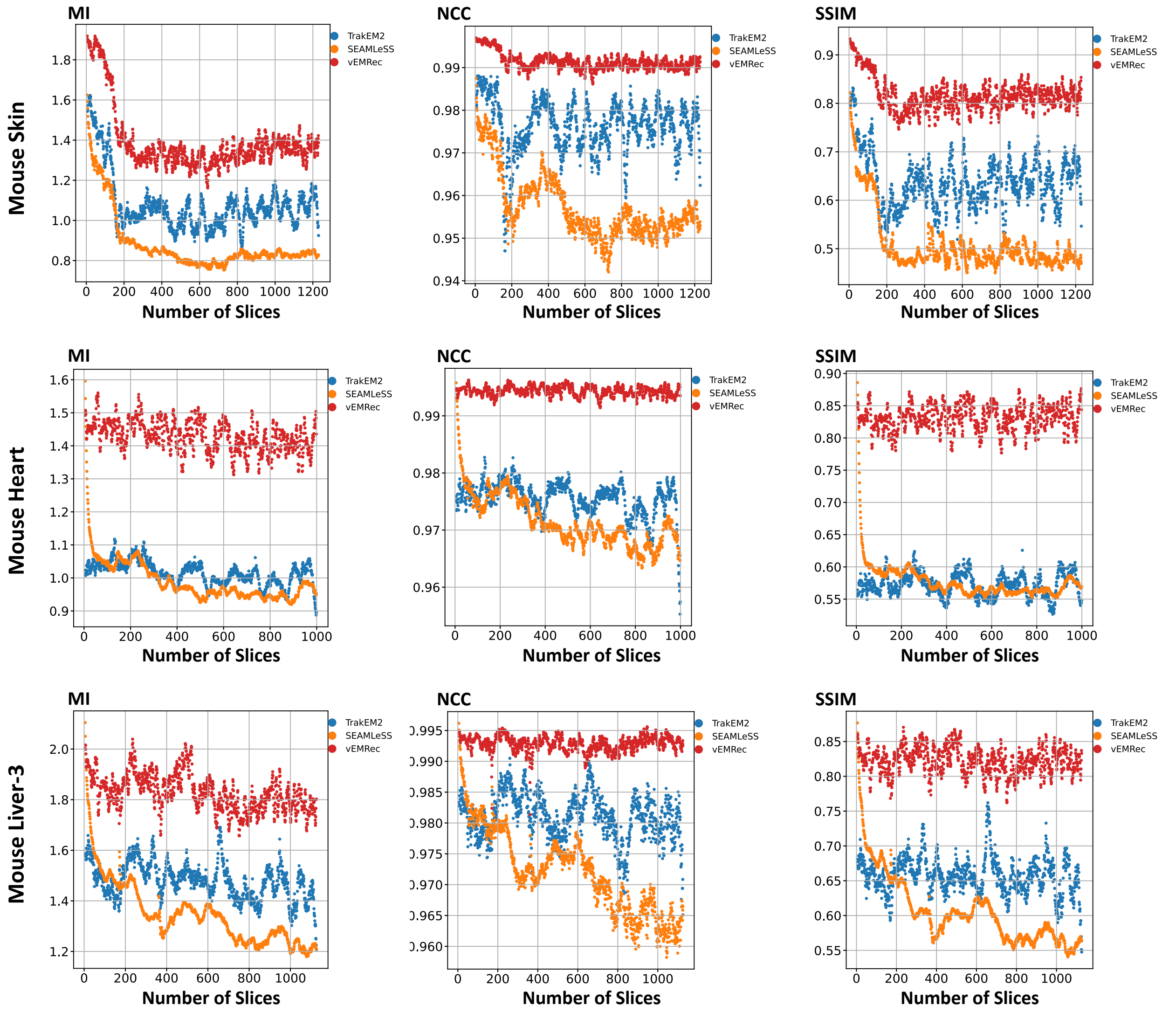


Figure S12: Comparison of MI(GT), NCC(GT), and SSIM(GT) metrics for various methods on the mouse skin, mouse liver-3, and mouse heart datasets as the number of slices increases.(deformation levels $\alpha$=1.0)

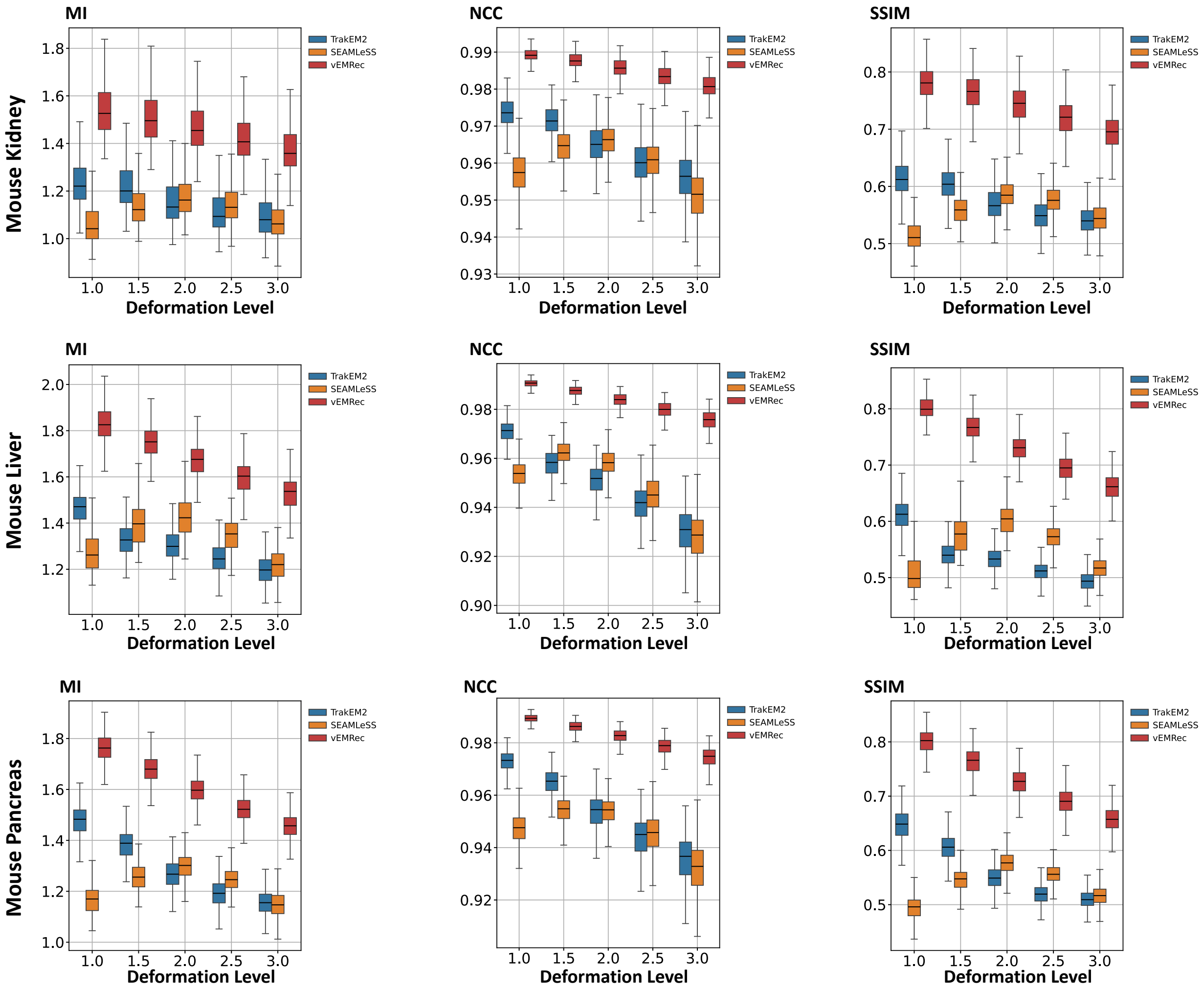


Figure S13: Comparison of MI(GT), NCC(GT), and SSIM(GT) for different methods on the mouse kidney, mouse liver, and mouse pancreas datasets under varying degrees of elastic deformation.

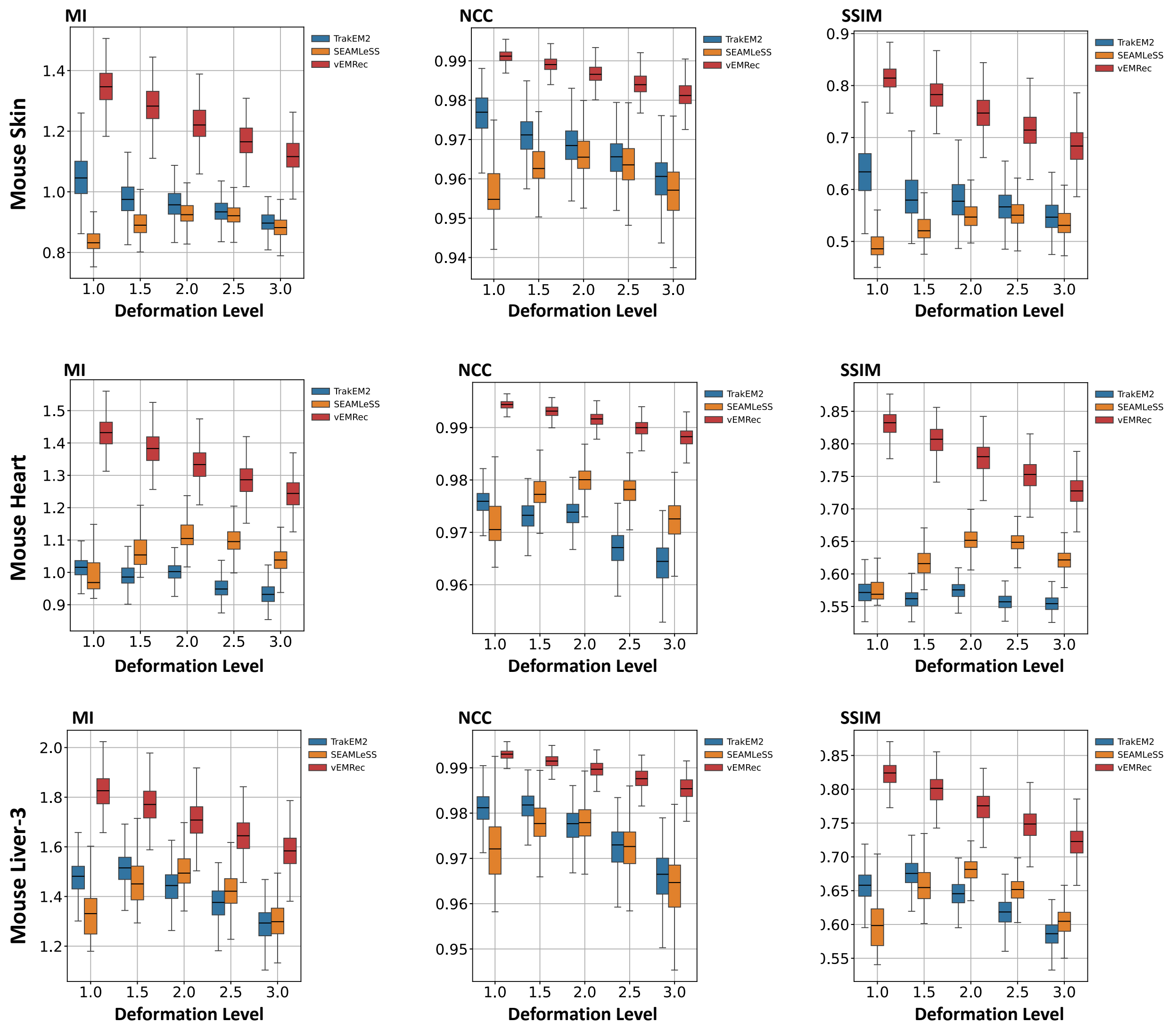


Figure S14: Comparison of MI(GT), NCC(GT), and SSIM(GT) for different methods on the mouse skin, mouse liver-3, and mouse heart datasets under varying degrees of elastic deformation.

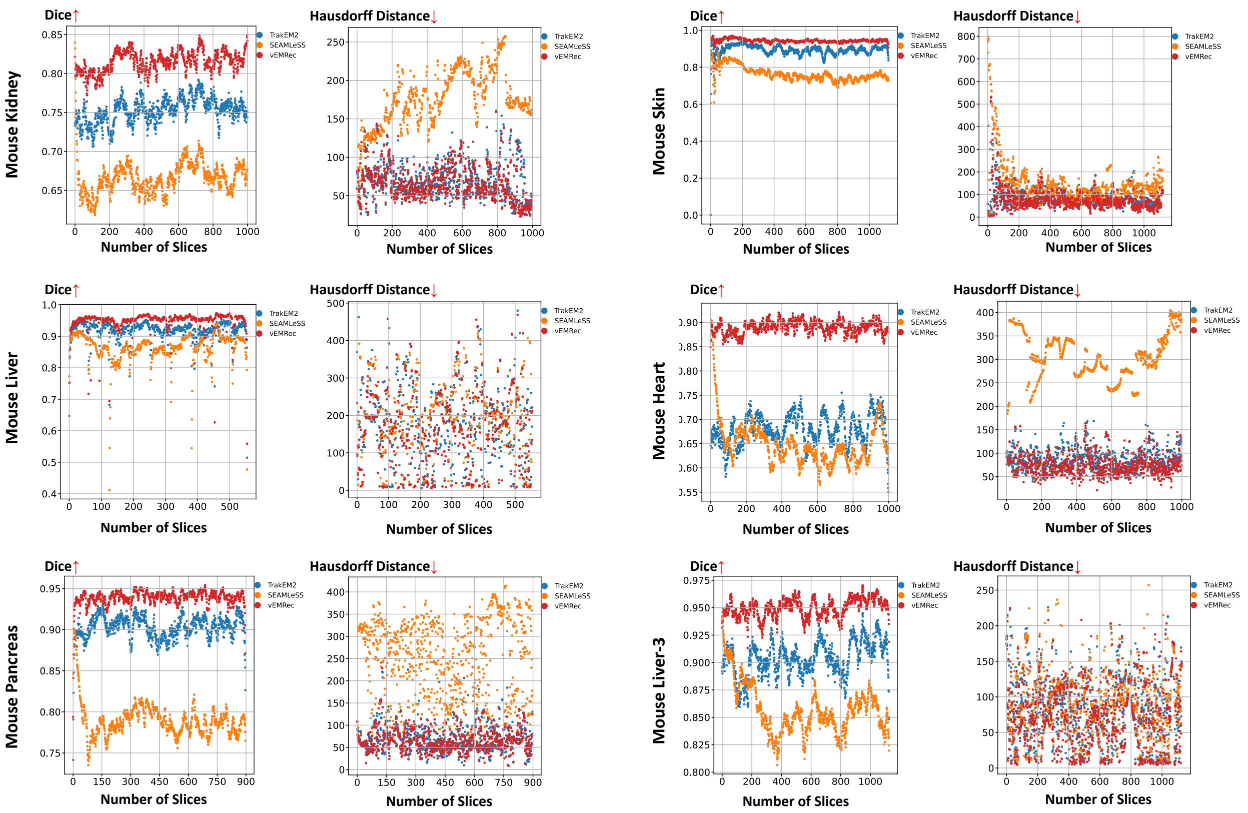


Figure S15: Comparison of Dice and Hausdorff Distance metrics for various methods across six datasets as the number of slices increases.

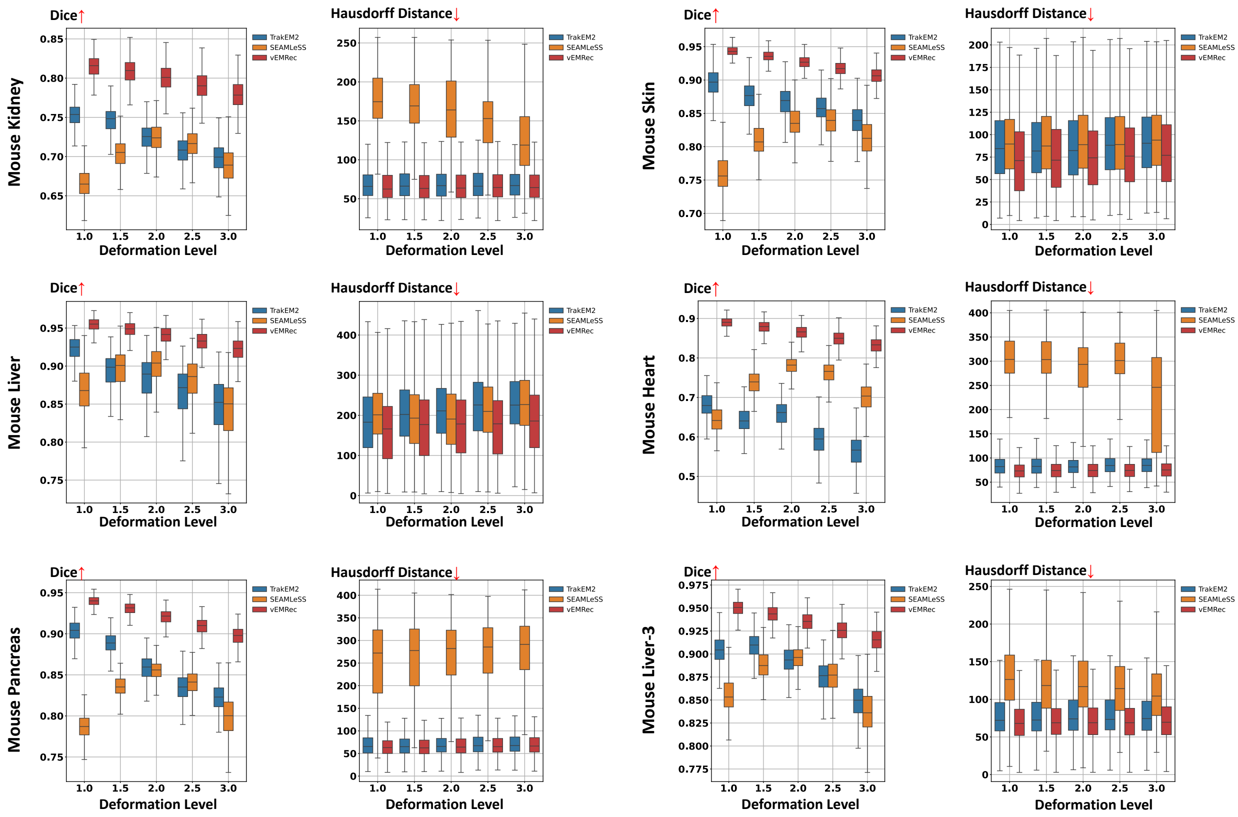


Figure S16: Comparison of Dice and Hausdorff Distance for different methods on the six datasets under varying degrees of elastic deformation.

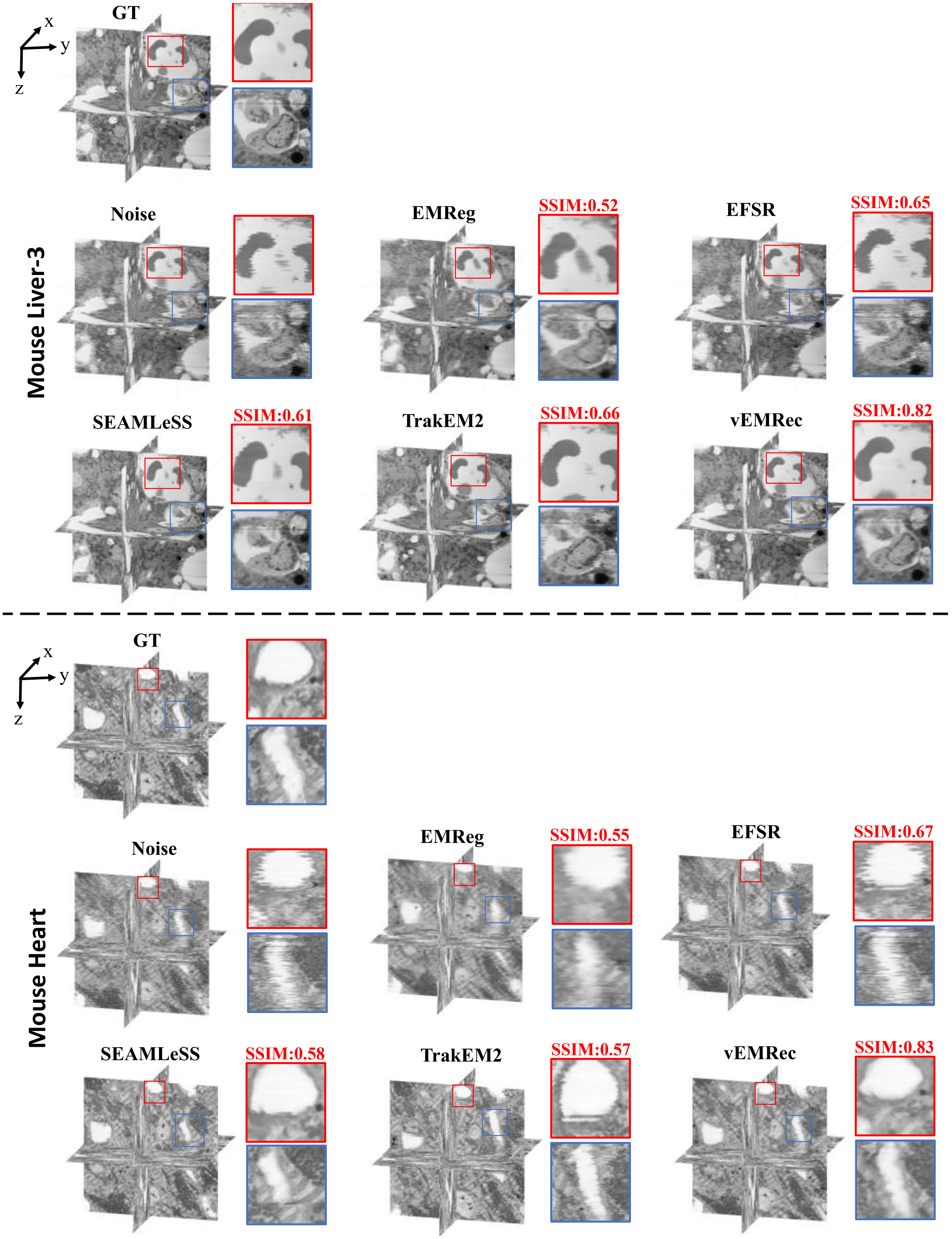


Figure S17: 3D visualization of the elastic registration results for different methods on the mouse liver-3 and mouse heart datasets.(deformation levels $\alpha$=1.0)

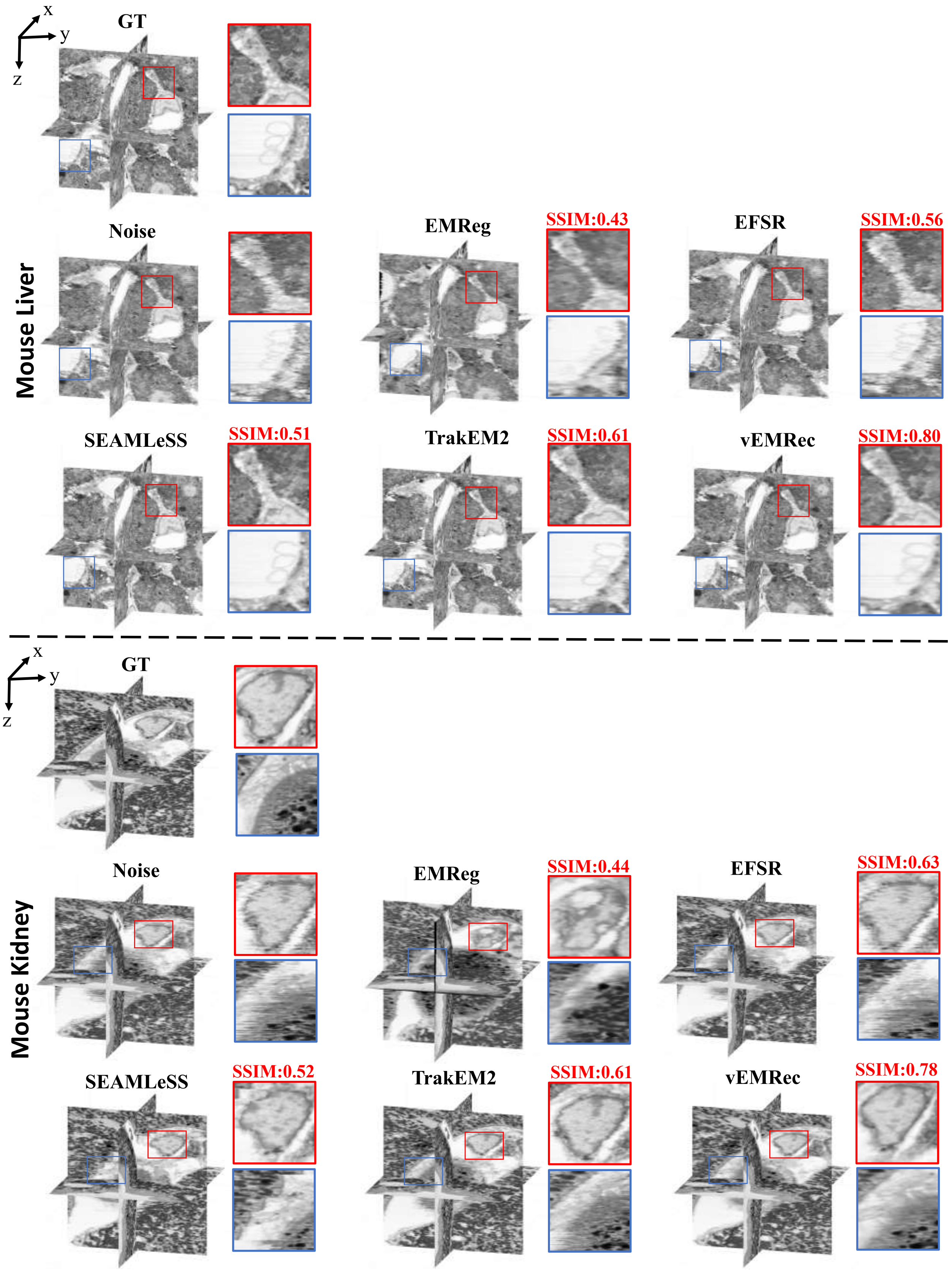


Figure S18: 3D visualization of the elastic registration results for different methods on the mouse liver and mouse kidney datasets.(deformation levels $\alpha$=1.0)

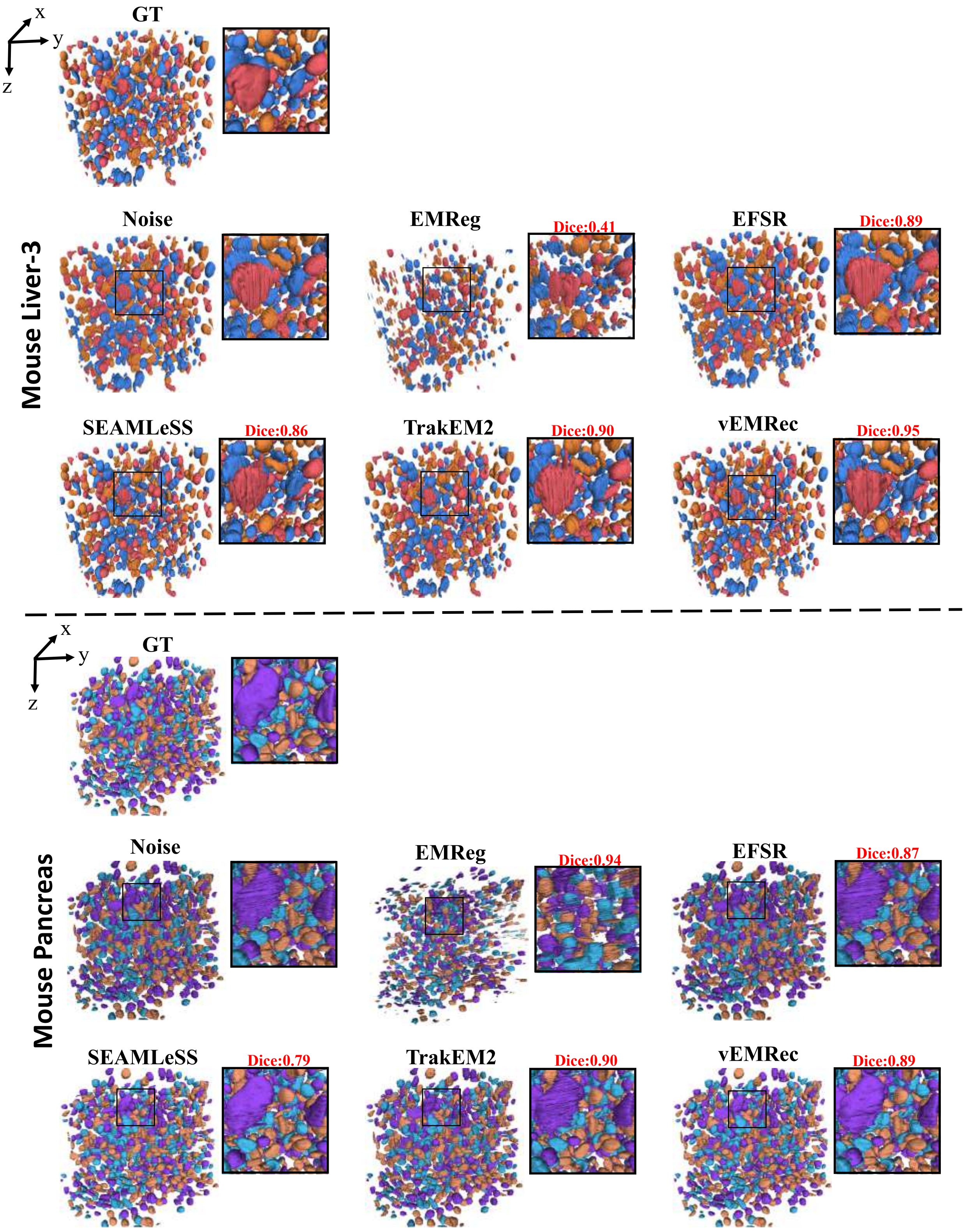


Figure S19: 3D segmentation visualization of elastic registration results for various methods on the mouse liver-3 and mouse pancreas datasets.

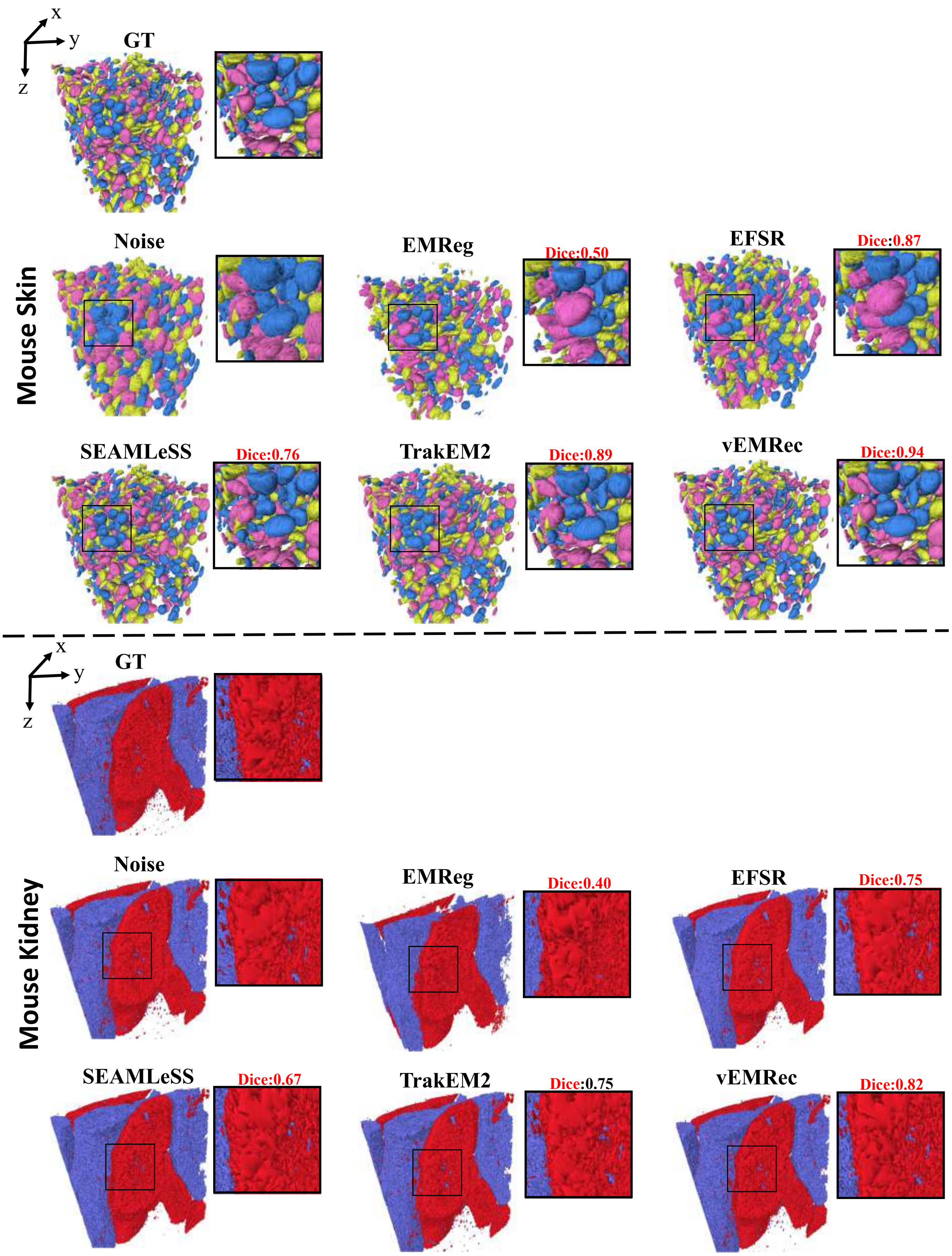


Figure S20: 3D segmentation visualization of elastic registration results for various methods on the mouse skin and mouse kidney datasets.

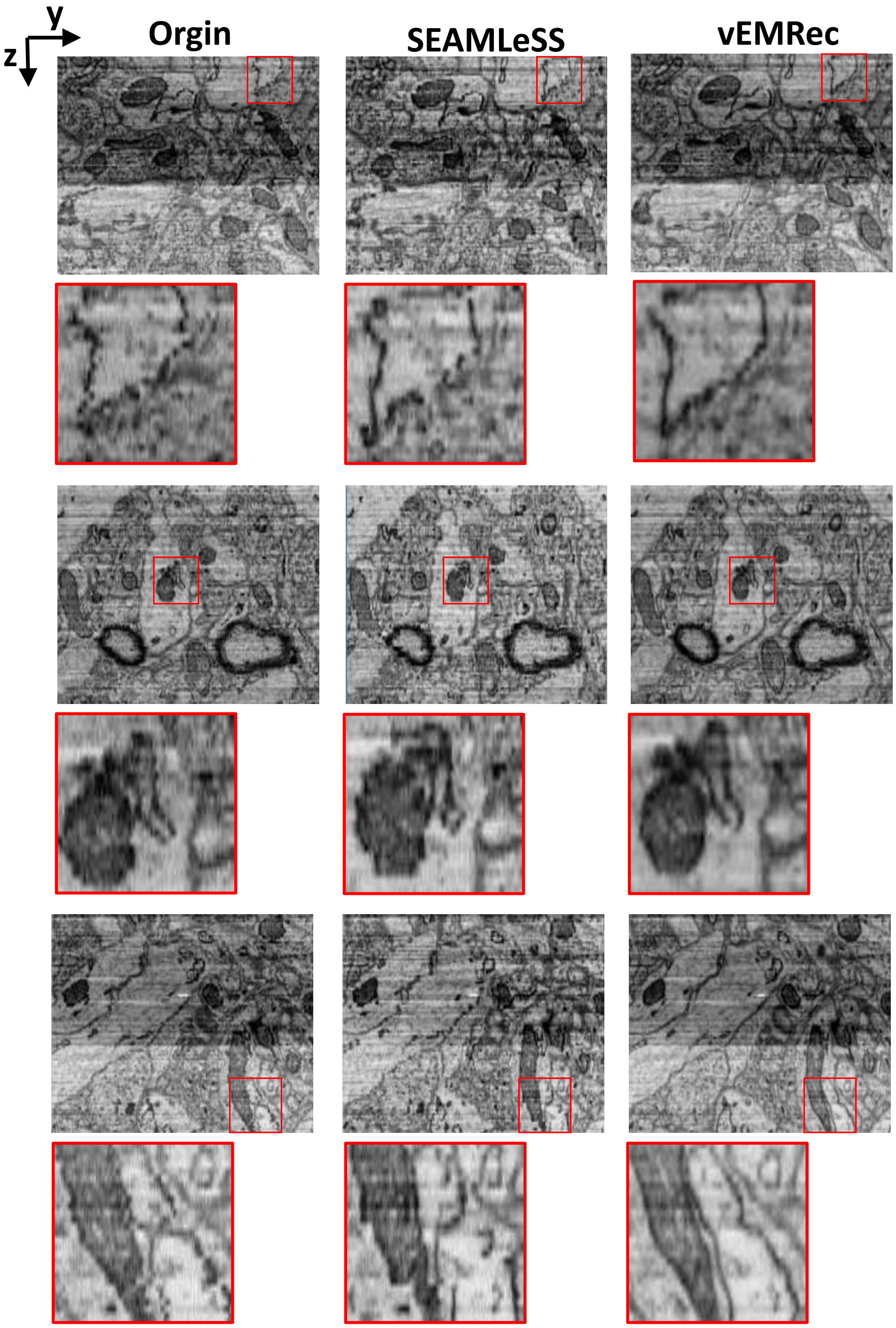


Figure S21: Side view of the original image data, SEAMLeSS registration result, and our registration result on FAFB dataset.

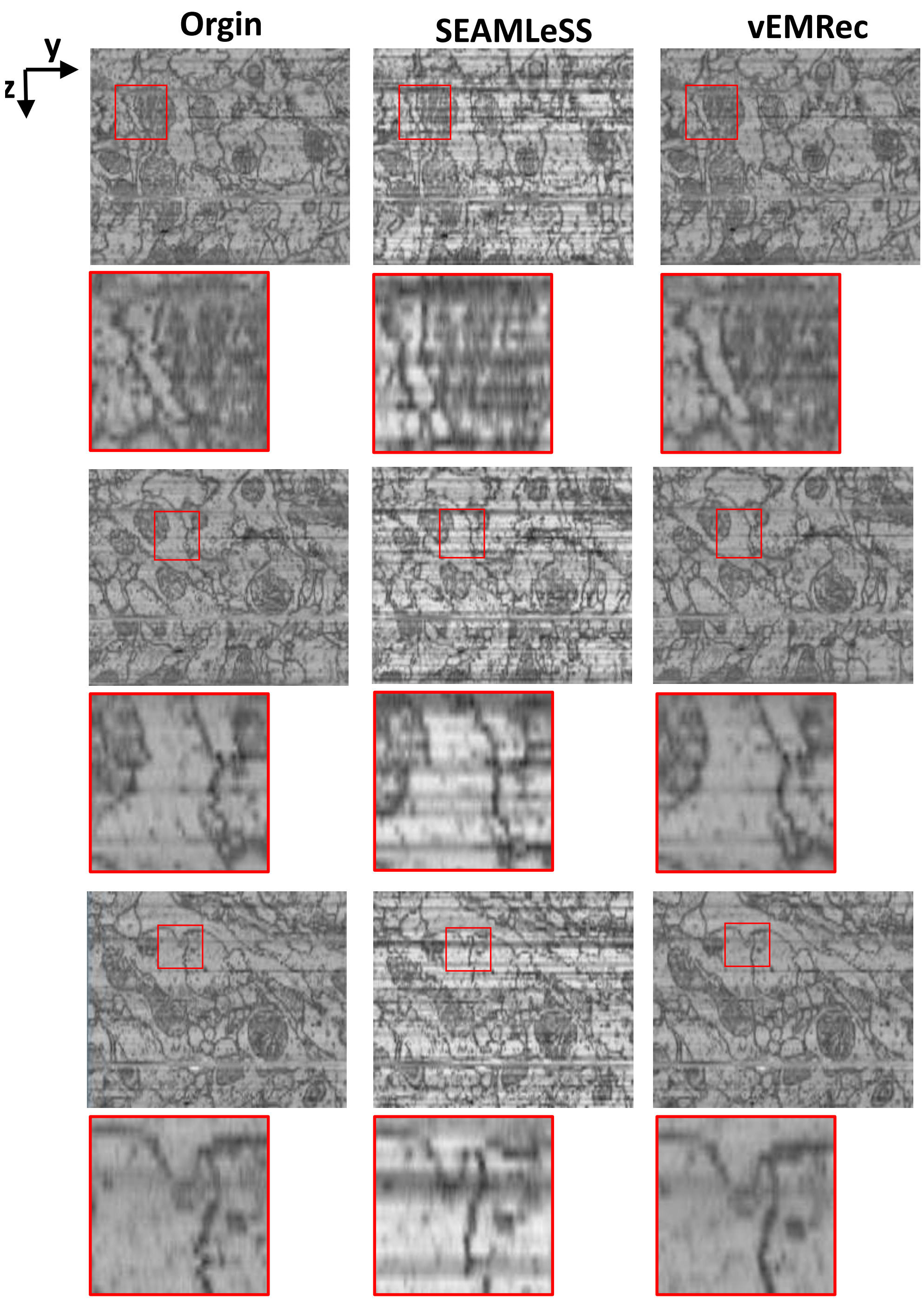


Figure S22: Side view of the original image data, SEAMLeSS registration result, and our registration result on mouse cortex dataset.

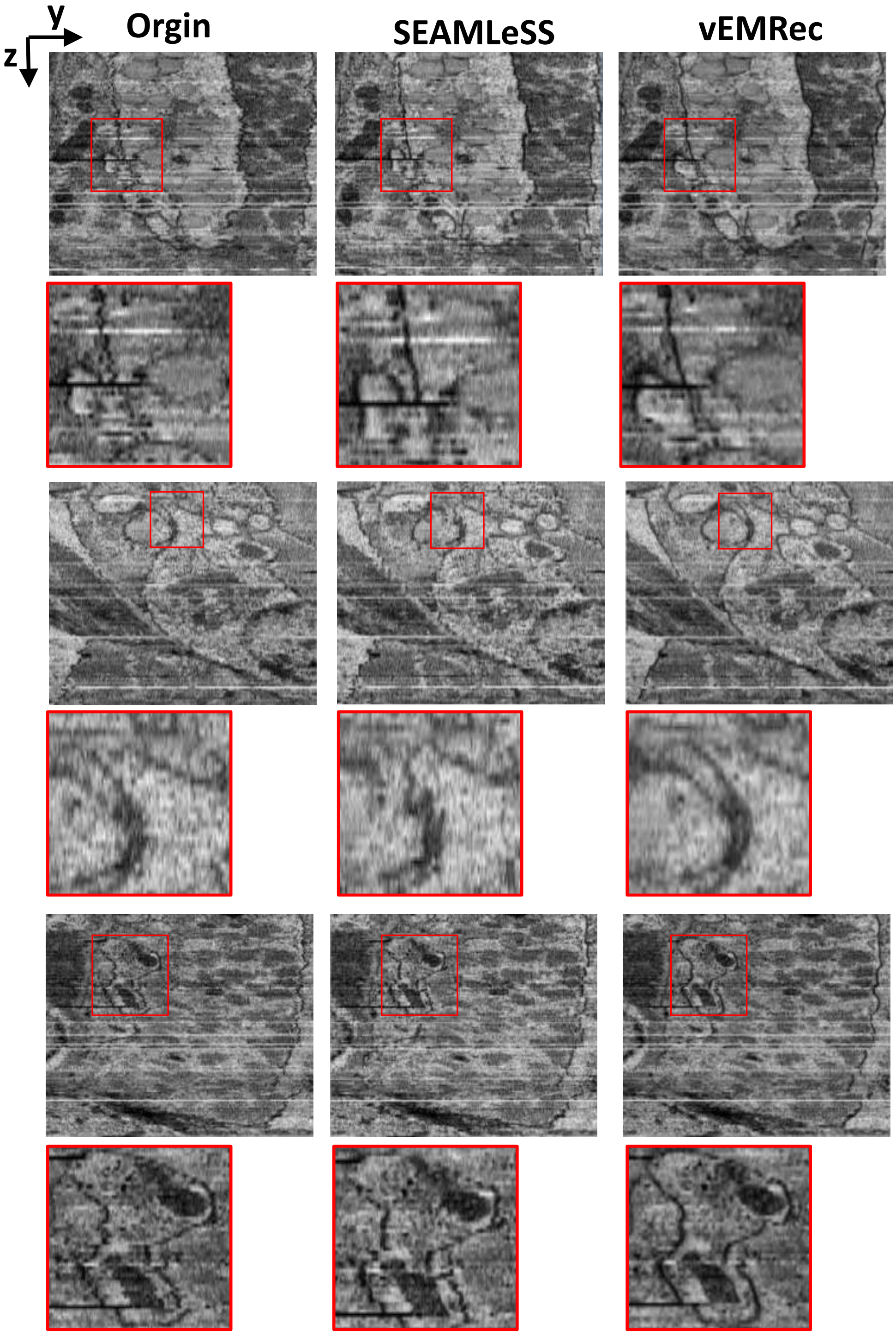


Figure S23: Side view of the original image data, SEAMLeSS registration result, and our registration result on C.elegans dataset.

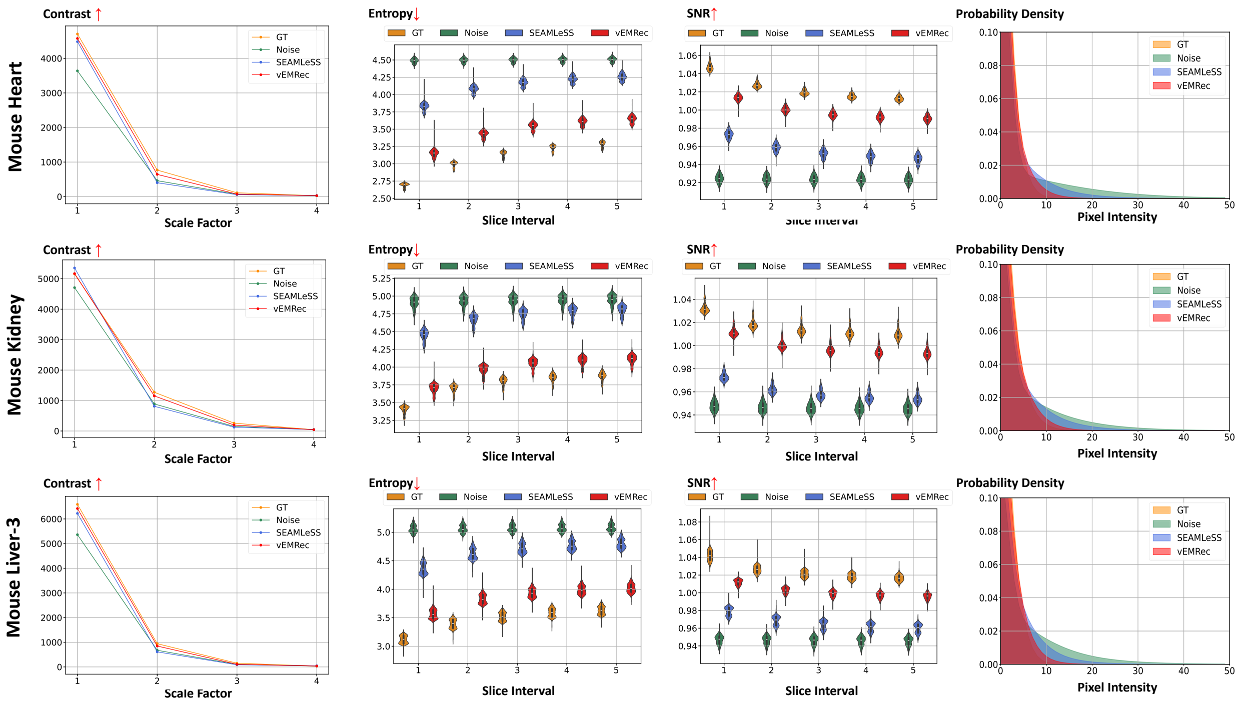


Figure S24: The results of the multi-scale difference filtering evaluation strategy on the simulated datasets of mouse heart, mouse kidney and mouse liver-3.

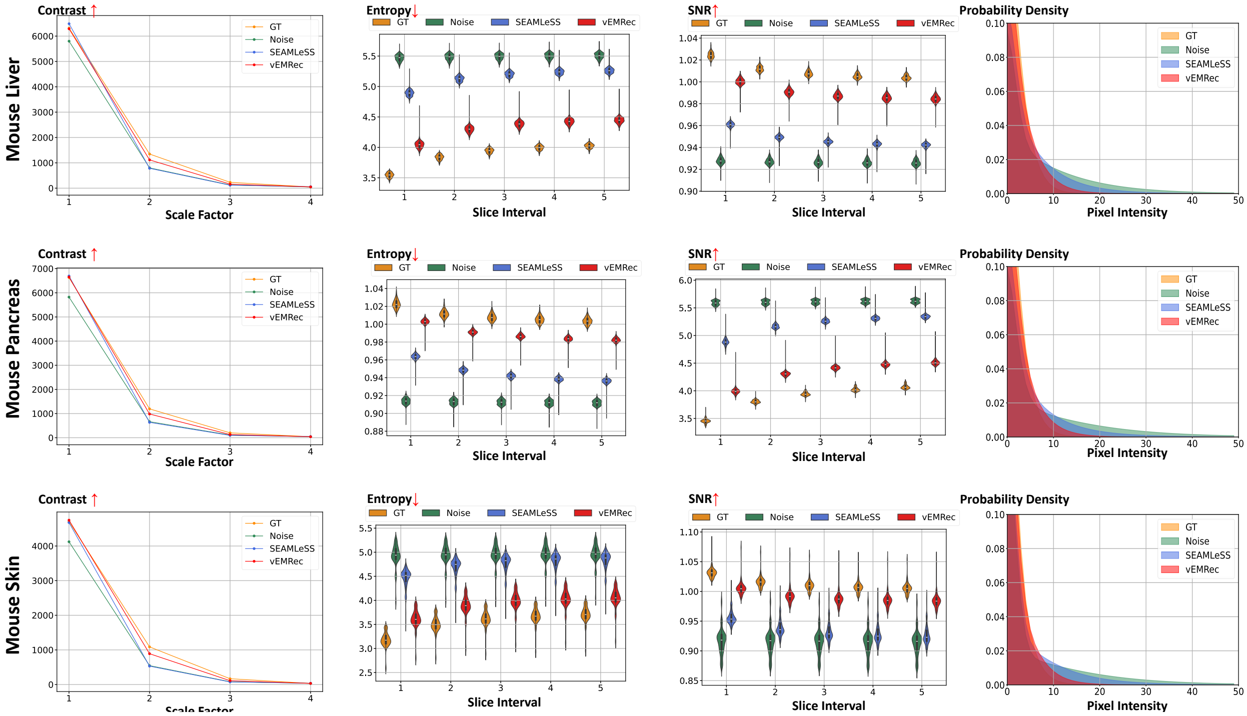


Figure S25: The results of the multi-scale difference filtering evaluation strategy on the simulated datasets of mouse liver, mouse pancreas and mouse skin.

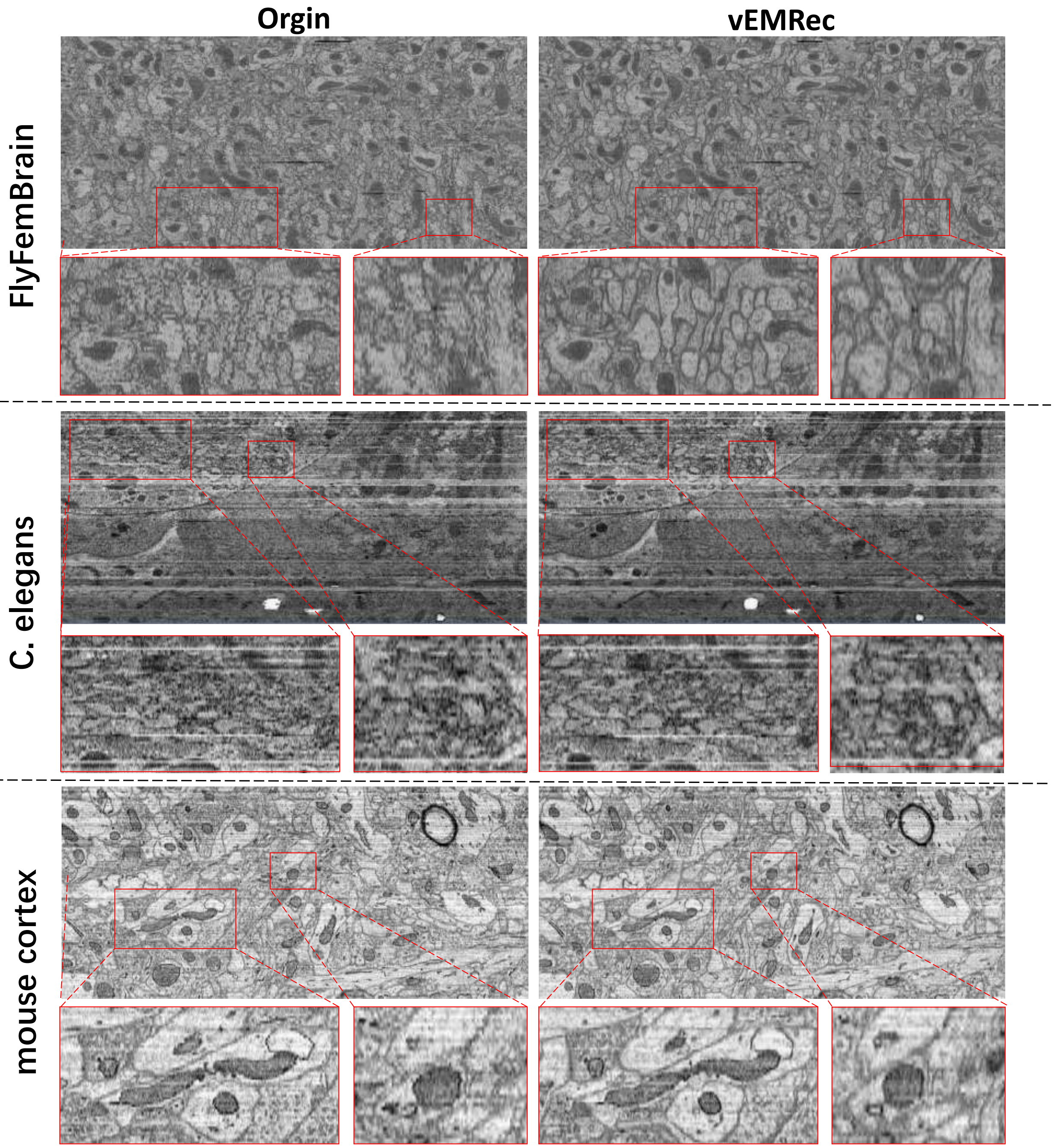


Figure S26: Side view of the isotropic reconstruction results on the FlyFemBrain, *C. elegans*, and mouse cortex datasets. In this experiment, we used only the simplest interpolation algorithm, yet the volumes reconstructed with vEMRec still exhibit clearly preserved ultrastructural features. These results demonstrate that the improved slice alignment produced by vEMRec leads to sharper, more coherent axial structures and reduces artifacts that would otherwise appear in isotropic reconstructions.

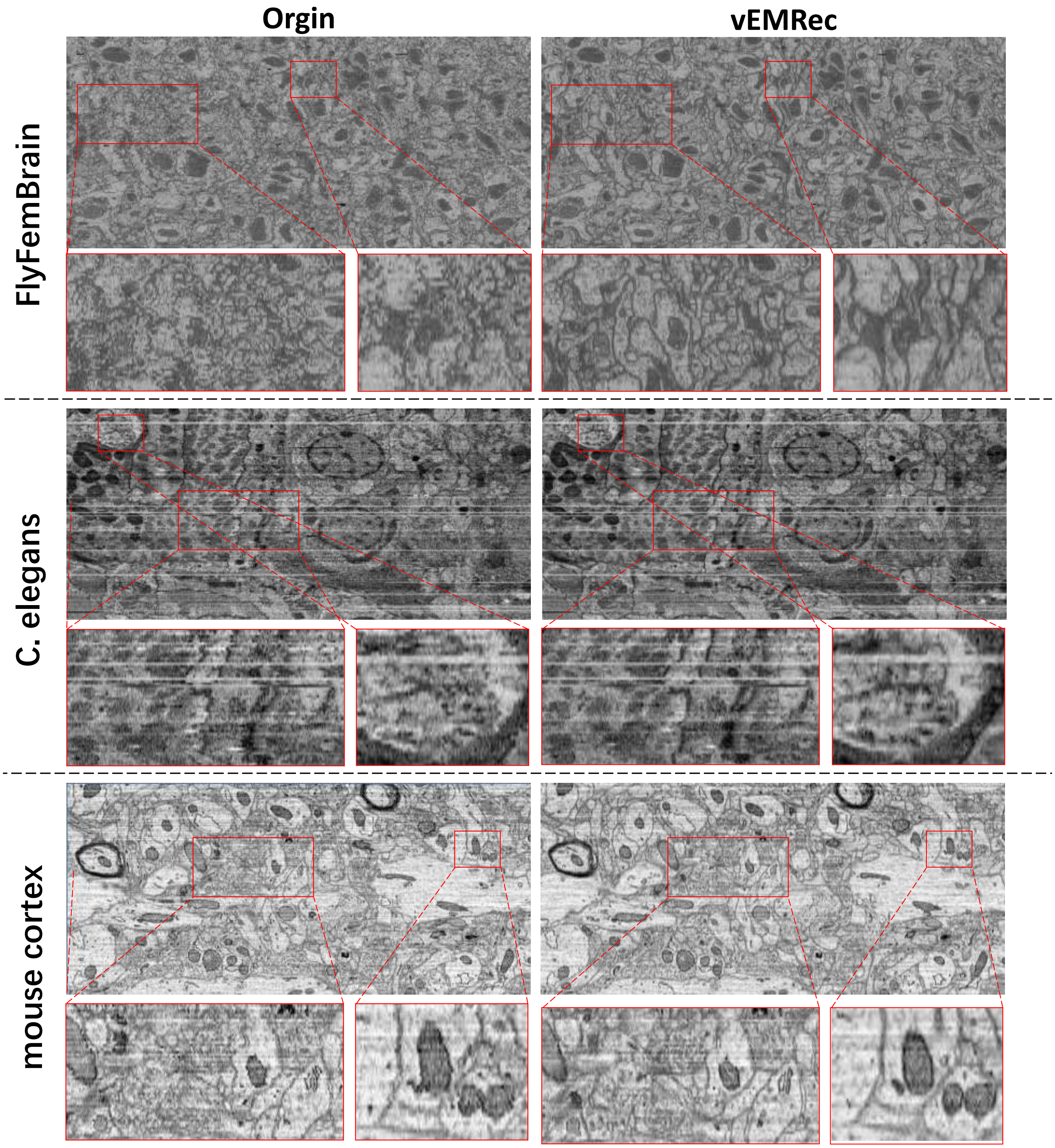


Figure S27: Side view of the isotropic reconstruction results on the FlyFemBrain, C.elegans, and mouse cortex dataset. In this case, we only used the simplest interpolation algorithm, yet the data reconstructed with vEMRec still exhibit clearly visible ultrastructural features.

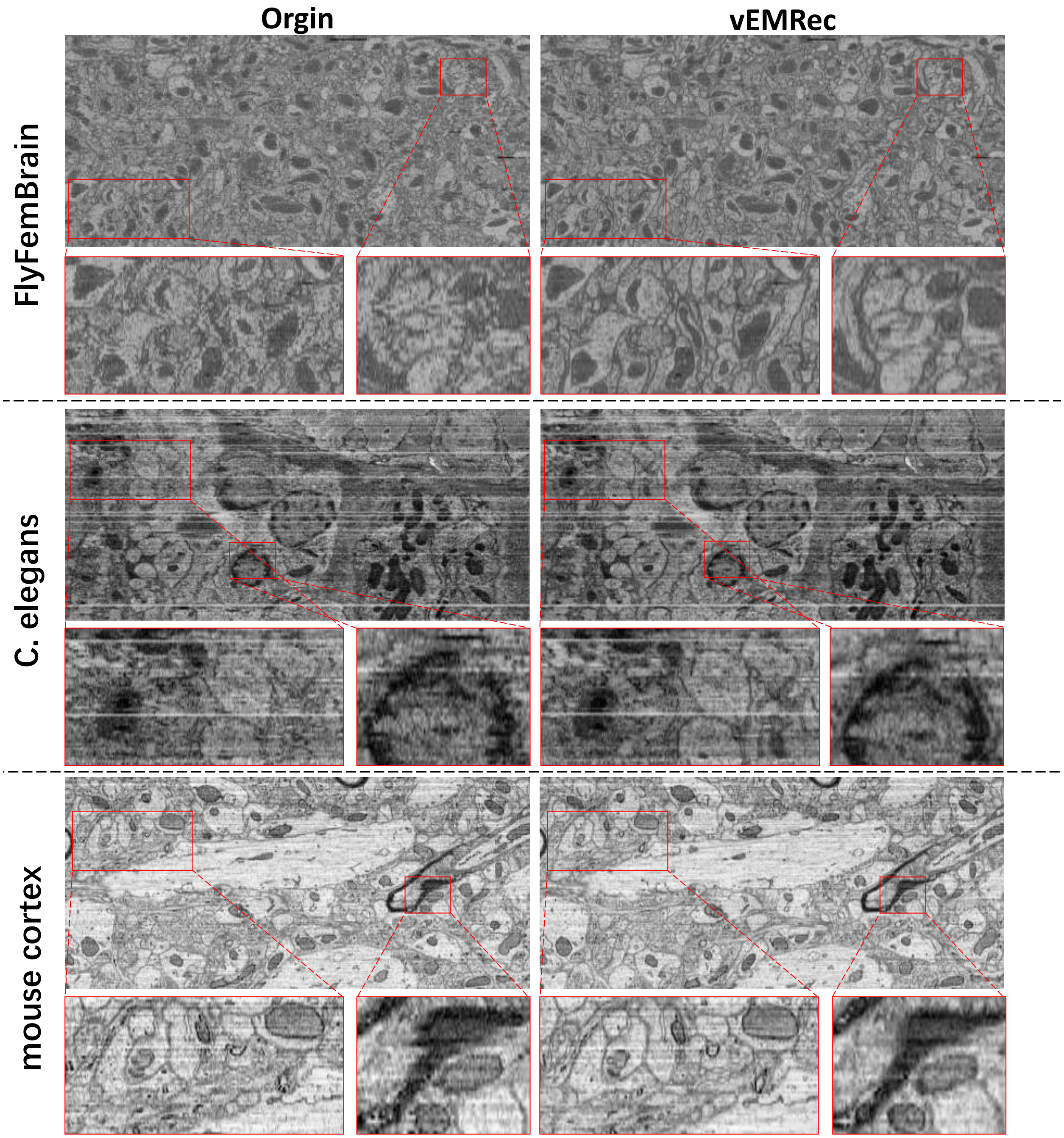


Figure S28: Side view of the isotropic reconstruction results on the FlyFemBrain, C.elegans, and mouse cortex dataset. In this case, we only used the simplest interpolation algorithm, yet the data reconstructed with vEMRec still exhibit clearly visible ultrastructural features.

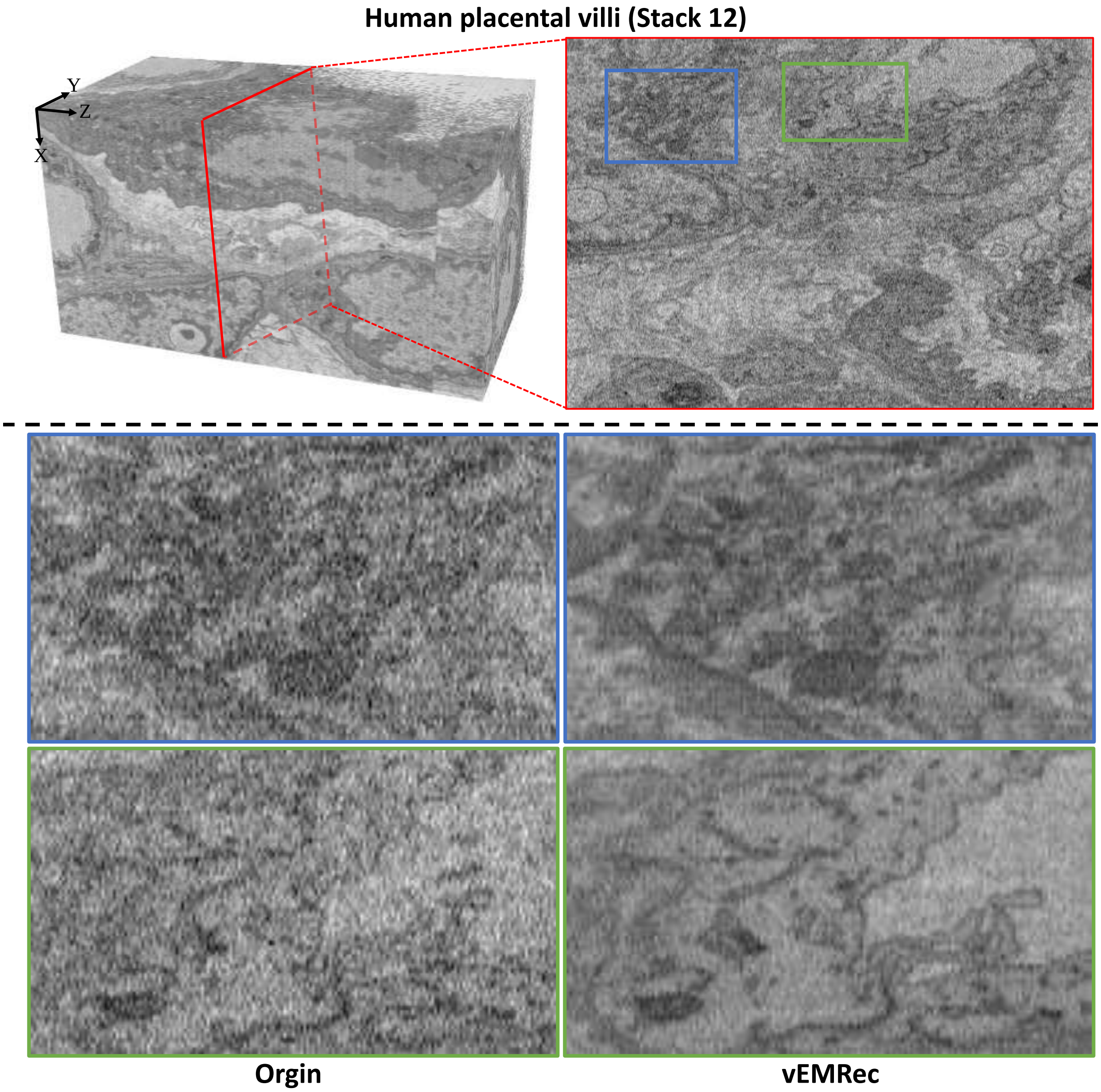


Figure S29: Side view of the isotropic reconstruction results on the Human term placental villi(Stack 12) dataset. In this case, we only used the simplest interpolation algorithm, yet the data reconstructed with vEMRec still exhibit clearly visible ultrastructural features.The Human term placental villi dataset is inherently low quality, with blurry and noisy images. After vEMRec alignment, cell boundaries become clearer and more continuous.

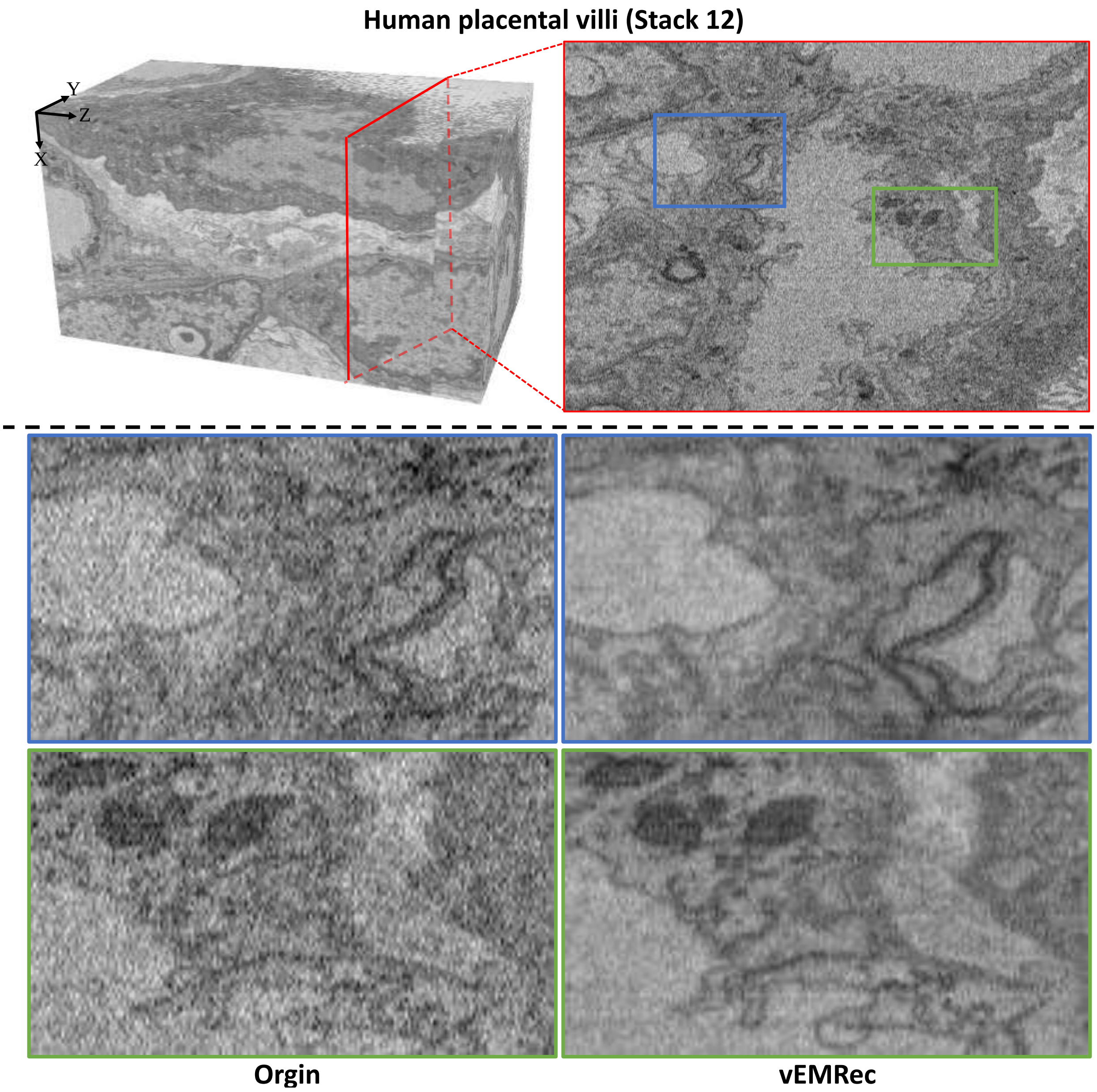


Figure S30: Side view of the isotropic reconstruction results on the Human term placental villi(Stack 12) dataset. In this case, we only used the simplest interpolation algorithm, yet the data reconstructed with vEMRec still exhibit clearly visible ultrastructural features. The Human term placental villi dataset is inherently low quality, with blurry and noisy images. After vEMRec alignment, cell boundaries become clearer and more continuous.

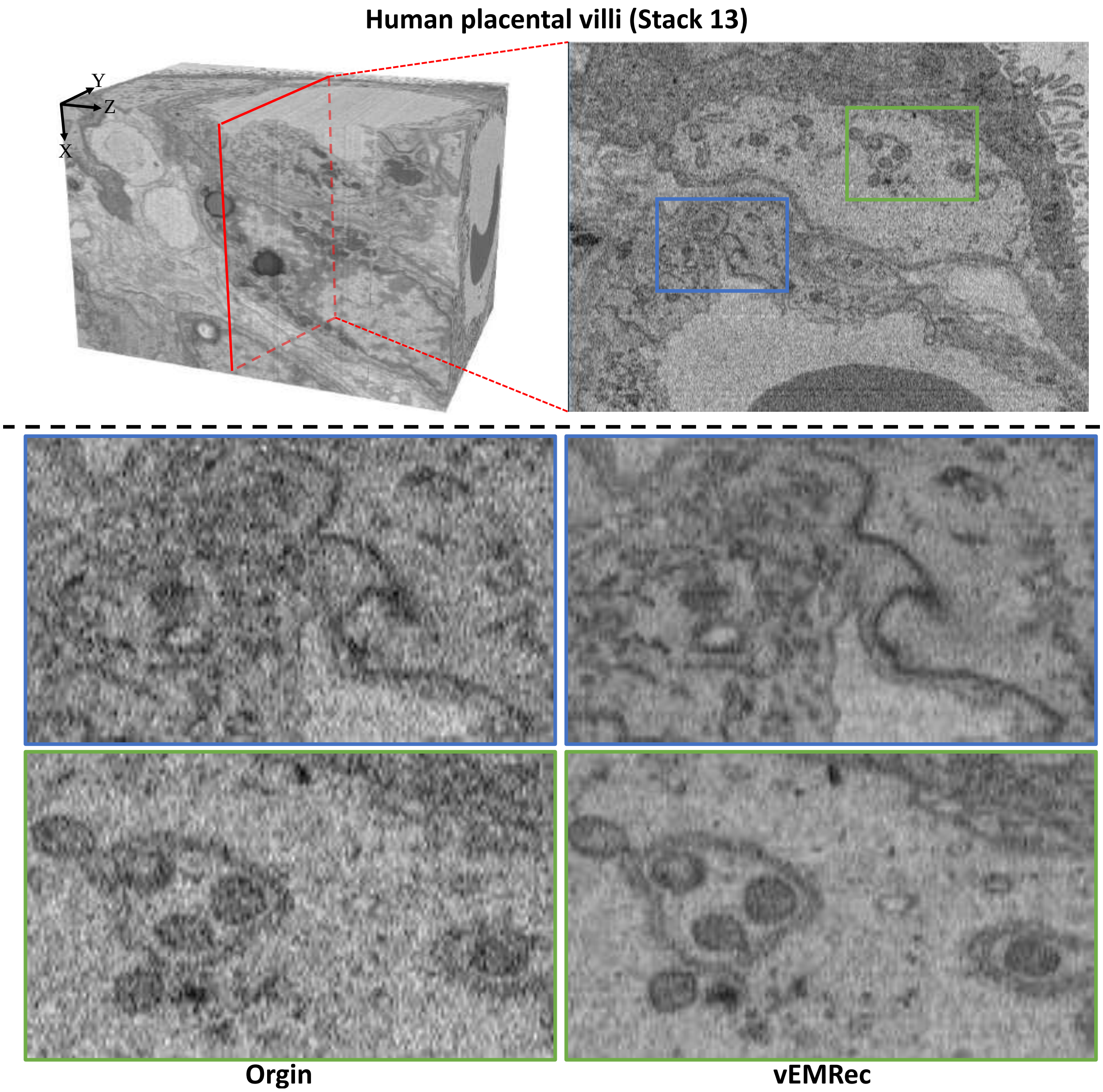


Figure S31: Side view of the isotropic reconstruction results on the Human term placental villi(Stack 13) dataset dataset. In this case, we only used the simplest interpolation algorithm, yet the data reconstructed with vEMRec still exhibit clearly visible ultrastructural features. The Human term placental villi dataset is inherently low quality, with blurry and noisy images. After vEMRec alignment, cell boundaries become clearer and more continuous.

**Human placental villi (Stack 13)**

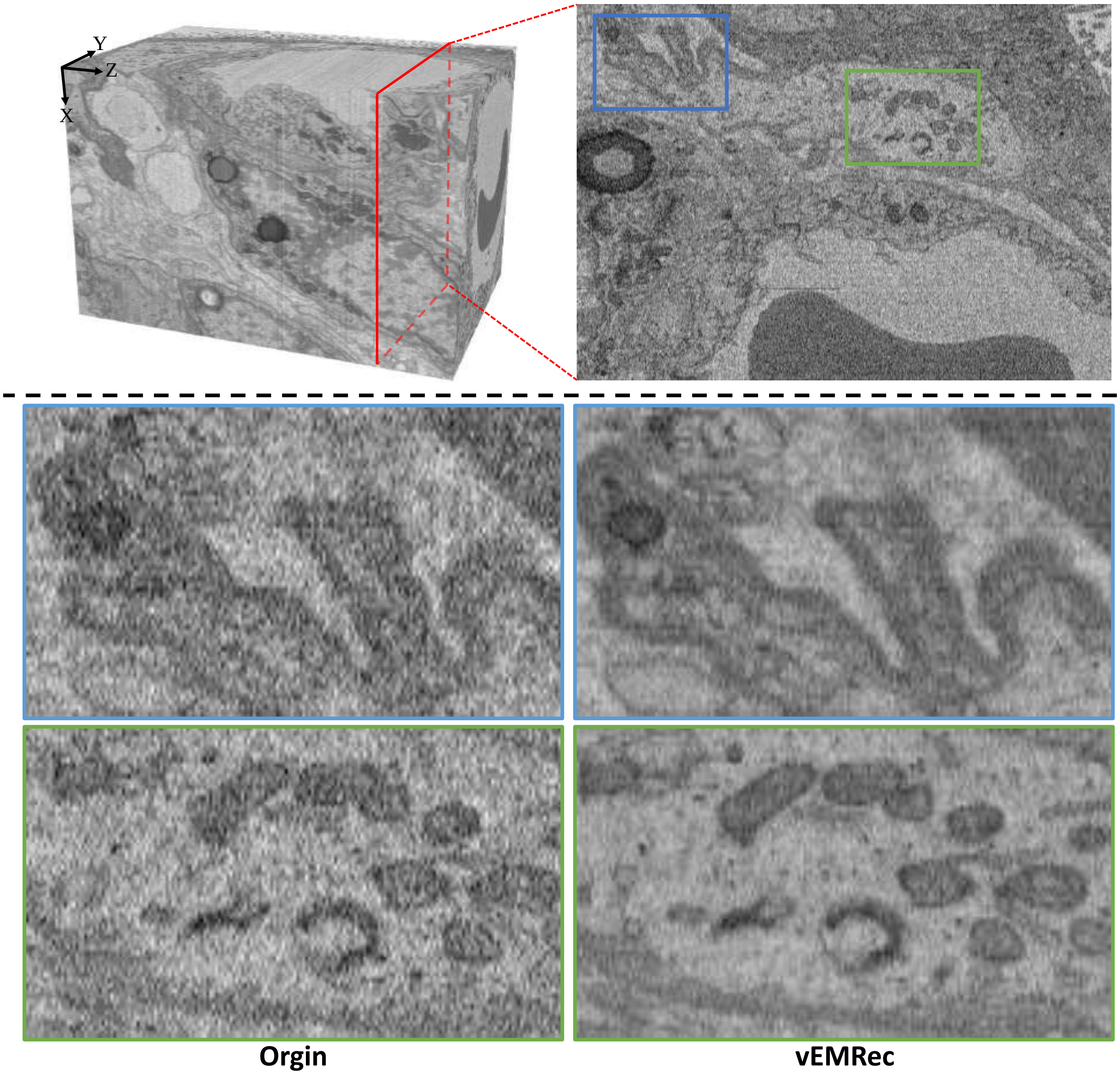


Figure S32: Side view of the isotropic reconstruction results on the Human term placental villi(Stack 13) dataset. In this case, we only used the simplest interpolation algorithm, yet the data reconstructed with vEMRec still exhibit clearly visible ultrastructural features. The Human term placental villi dataset is inherently low quality, with blurry and noisy images. After vEMRec alignment, cell boundaries become clearer and more continuous.

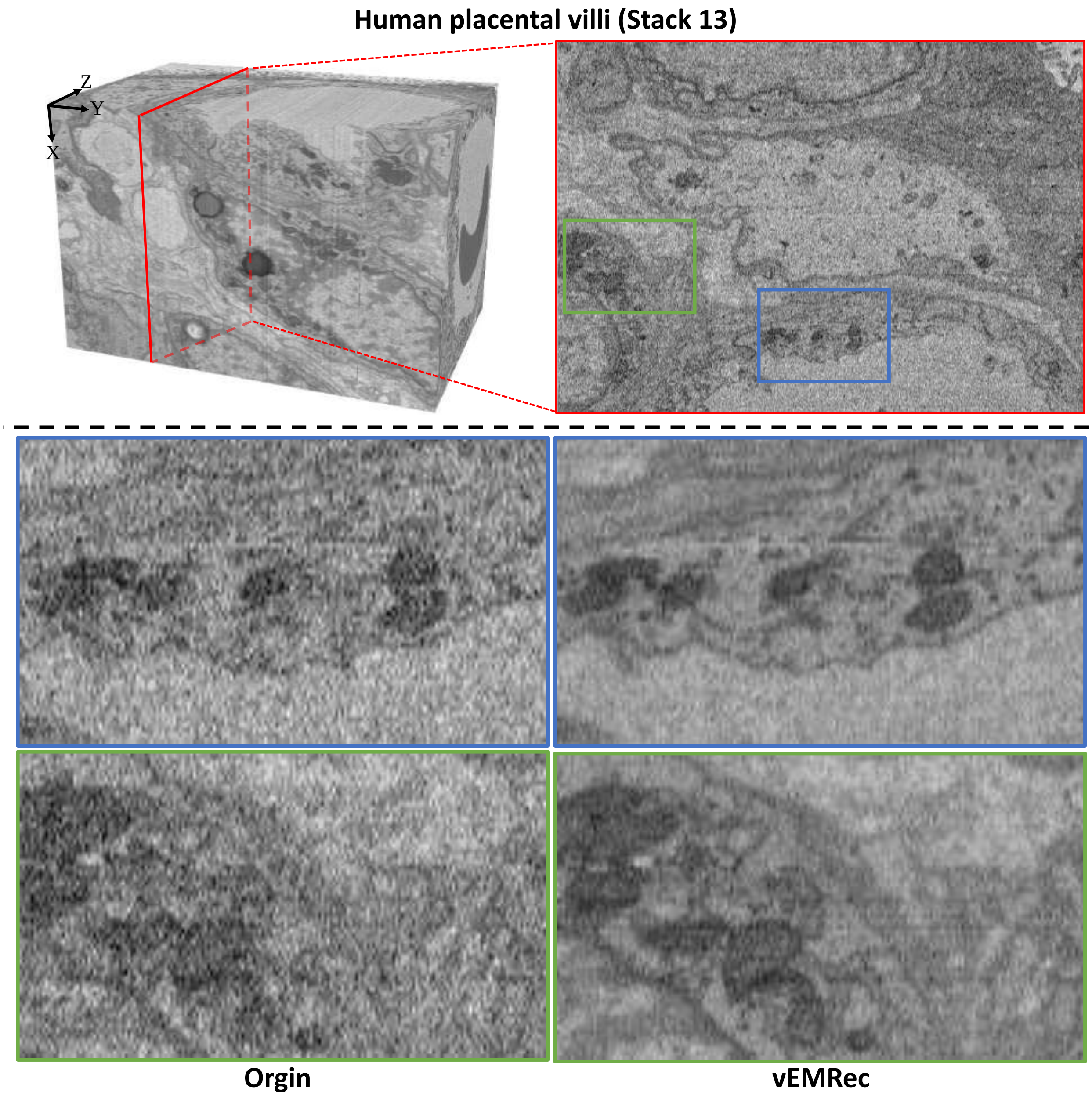


Figure S33: Side view of the isotropic reconstruction results on the Human term placental villi(Stack 13) dataset. In this case, we only used the simplest interpolation algorithm, yet the data reconstructed with vEMRec still exhibit clearly visible ultrastructural features. The Human term placental villi dataset is inherently low quality, with blurry and noisy images. After vEMRec alignment, cell boundaries become clearer and more continuous.

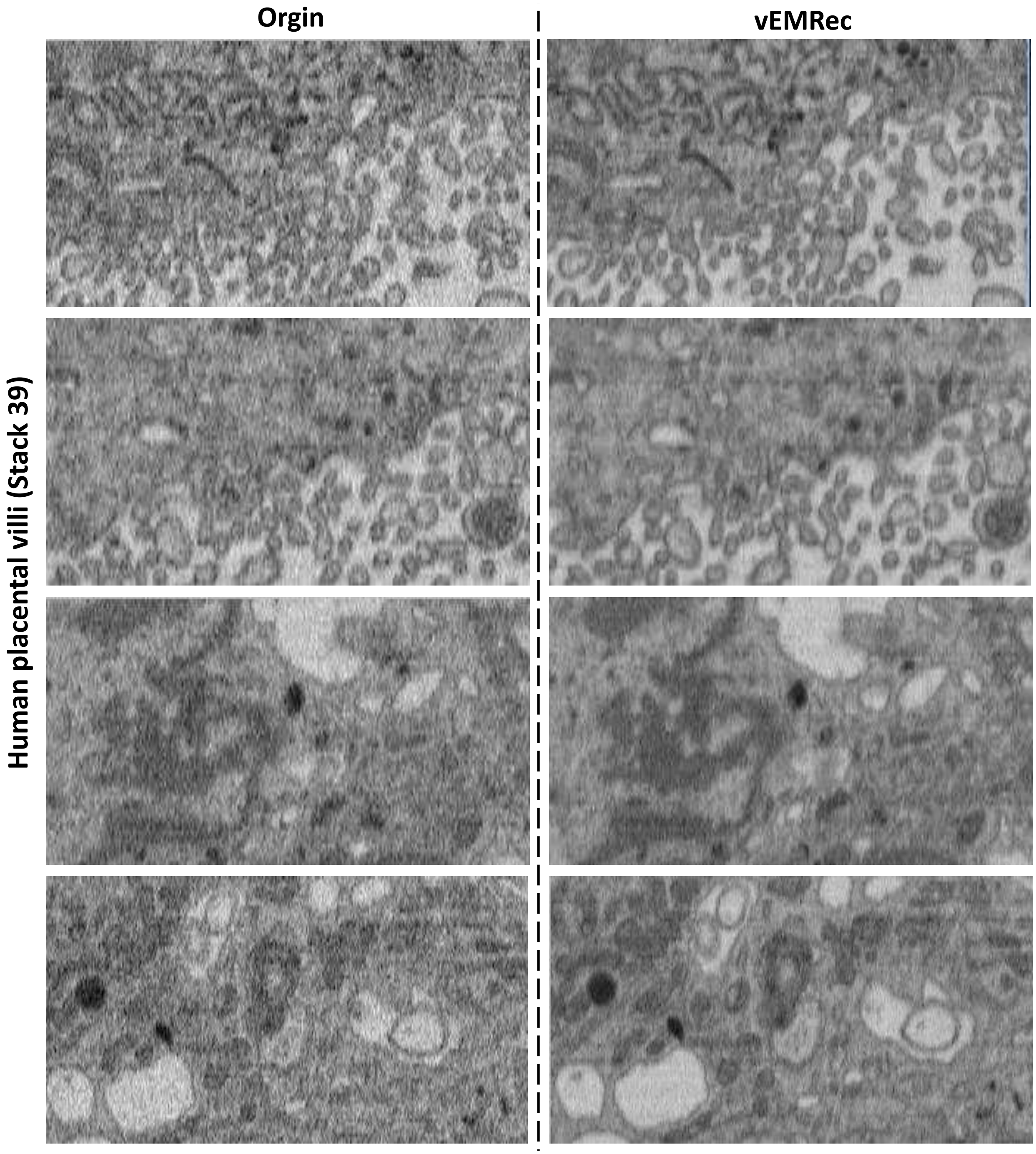


Figure S34: Enlarged side-view visualization of the Human term placental villi dataset (Stack 39), demonstrating consistent structural refinement achieved by vEMRec across different stacks.

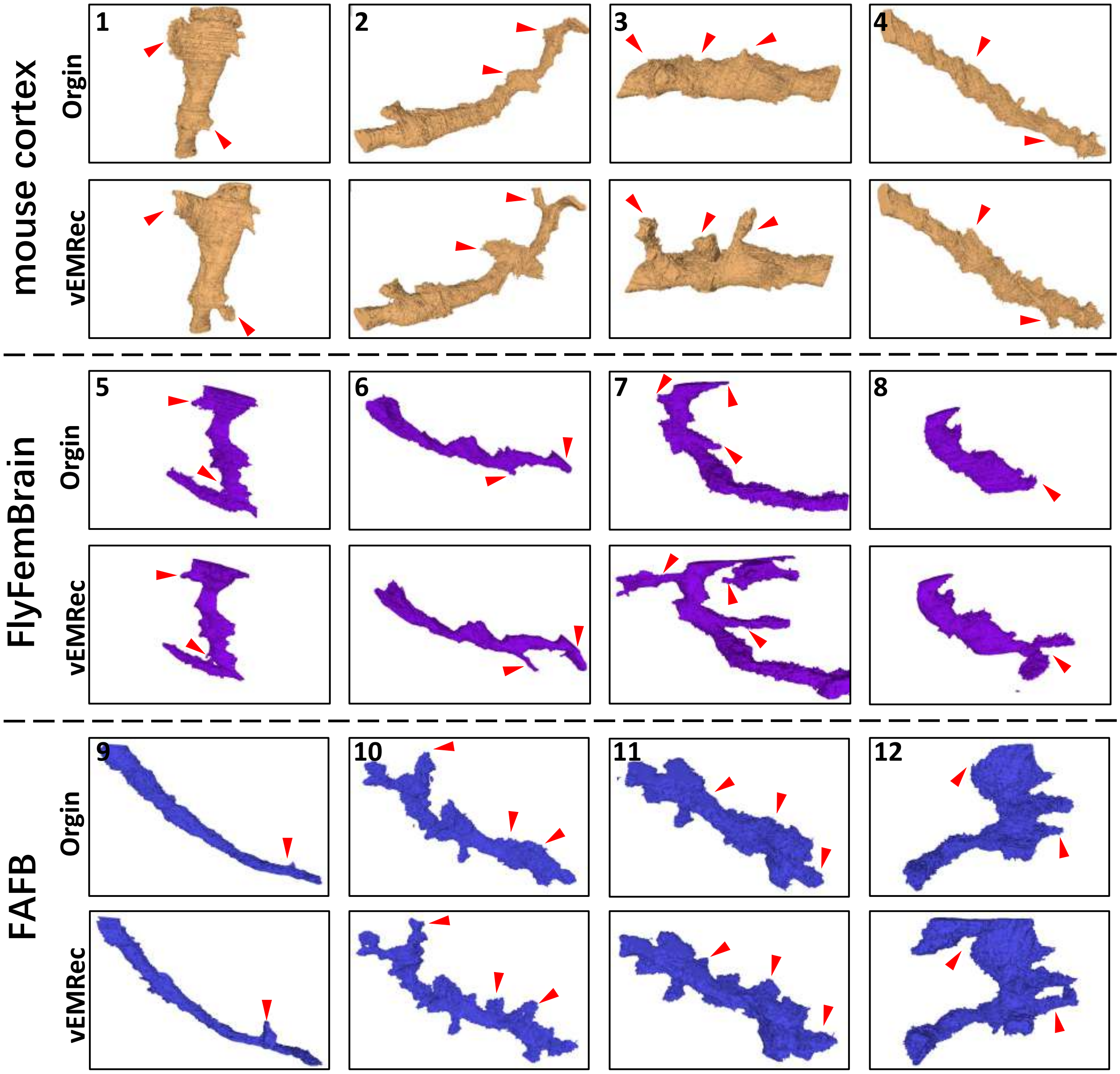


Figure S35: Neuron segmentation results on the FAFB, FlyFemBrain and mouse cortex datasets. Segmentation is performed on both raw and vEMRec-registered data. The vEMRec-aligned data yield more accurate branching structures, facilitating improved anatomical fidelity and potential scientific insights.

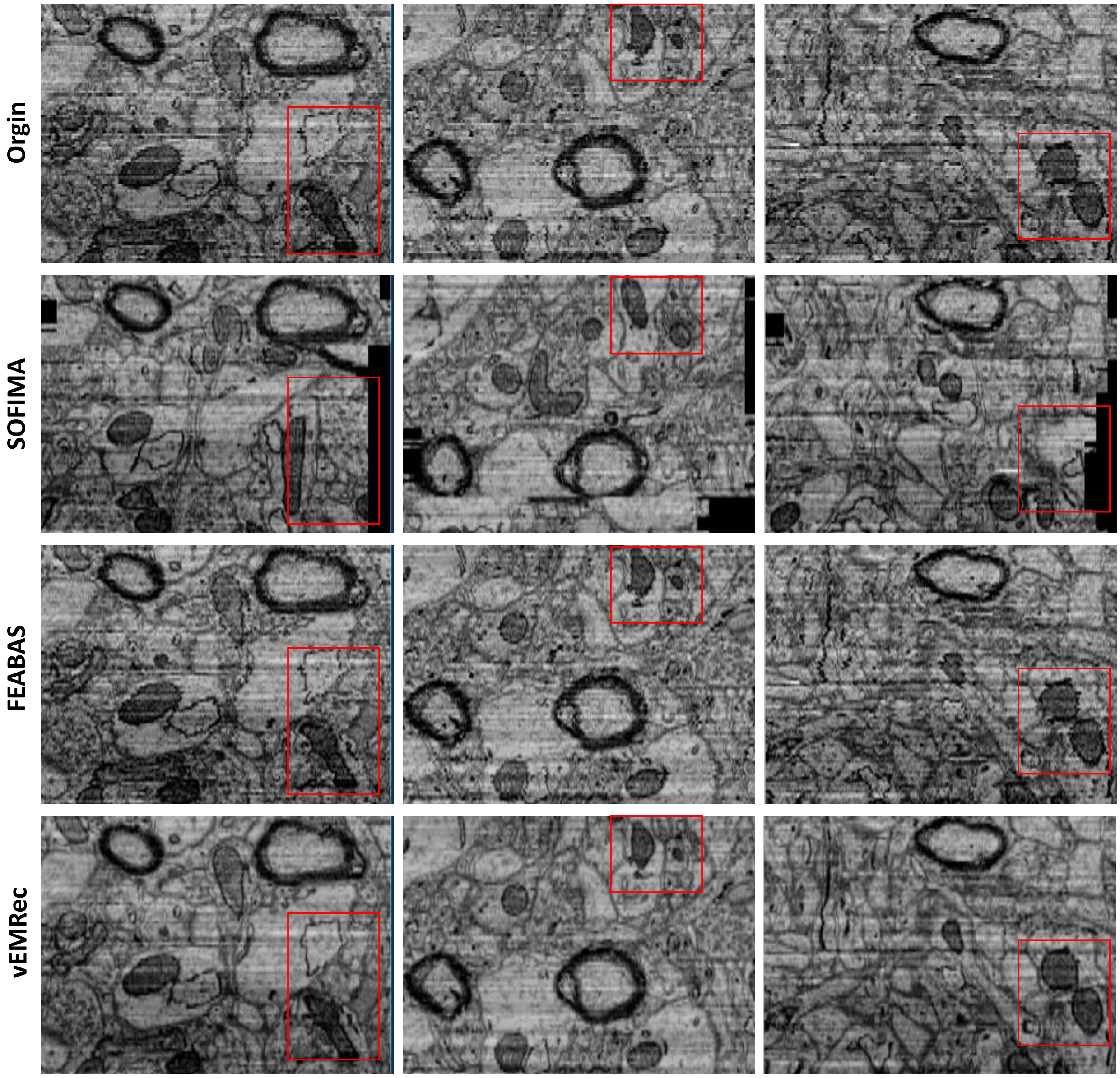


Figure S36: Side-view visualization on the mouse cortex dataset, showing the original data and the alignment results produced by SOFIMA, FEABAS, and vEMRec. Please zoom in for a clearer comparison.

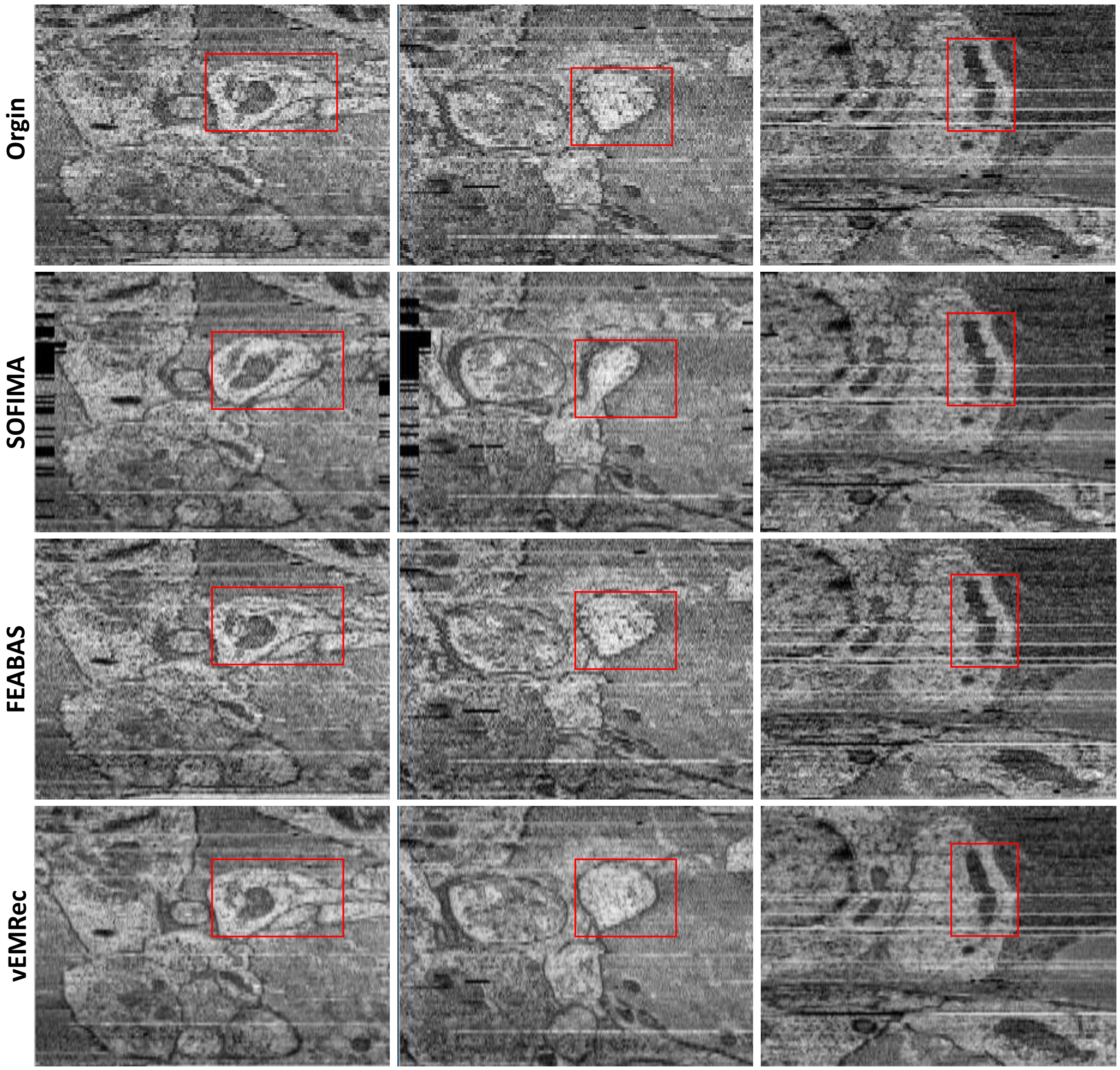


Figure S37: Side-view visualization on the Caenorhabditis elegans cell dataset, showing the original data and the alignment results produced by SOFIMA, FEABAS, and vEMRec. Please zoom in for a clearer comparison.

## S3 Neural Network Architectures

### S3.1 Optical flow estimation network

We use a cascaded optical flow estimation network to estimate the deformation field between slices using a coarse-to-fine strategy. As demonstrated in Figure S38, we break down the generation of the deformation field $\varphi_{m,f}$ into $T$ iterations. For each iteration, such as the $t$-th iteration, the input to the network is the fixed image $I_f$ and warped image $\hat{I}_{m,t-1}$ warped by the deformation field $\varphi_{m,t-1}$ from the previous iteration. To calculate the new deformation field, a cascaded architecture is adopted where a 2D convolution encoder is adopted to extract feature pyramids. Specifically, the encoder processes $\hat{I}_{m,f,t-1}$ to generate a feature pyramid $\{F_m^s\}_{s=0}^3$, and it simultaneously extracts both a feature pyramid $\{F_f^s\}_{s=0}^3$ and a contextual feature pyramid $\{C_f^s\}_{s=1}^3$ from $I_f$. The dimensions of $F_m^0$, $F_m^1$, $F_m^2$, and $F_m^3$ are $1/2$, $1/4$, $1/8$, and $1/16$ of $I_m$, respectively, with channel numbers of 8, 16, 32, and 64. The number of channels in each layer of $\{C_f^s\}_{s=1}^3$ and $\{F_f^s\}_{s=0}^3$ is kept consistent with that of $\{F_m^s\}_{s=0}^3$.

Given the feature pyramid $\{F_f^s\}_{s=1}^3$, $\{F_m^s\}_{s=1}^3$, and $\{C_f^s\}_{s=1}^3$, we divide them into three sets of features based on their scale, denoted as $\{F_f^s, F_m^s, C_f^s\}_{s=1}^3$. These features are then separately input into three individual recurrent generation modules (ConvGRU). Simultaneously, each ConvGRU takes the deformation field $\varphi_{m,f,t}^{s+1}$ as supplementary input, achieving a coarse-to-fine update of the deformation field $\varphi_{m,f,t}^s$.

In the final stage of the network, we input the deformation field $\varphi_{m,f,t}^1$ and $\{F_f^0, F_m^0\}$ into the Context Fusion Module, thereby introducing broader contextual information to enhance the accuracy of the deformation field. The Context Fusion Module is a simple U-net network and ultimately outputs the refined deformation field $\varphi_{m,f,t}$.

Our loss function is composed of two primary components: similarity loss $L_s$ and regularization loss $L_r$. The similarity loss, $L_s$, is formulated to compute the weighted similarity across $k$ adjacent slices. The regularization loss $L_r$ focuses on the deformation fields $\varphi_{m,f,t}$ obtained at each iteration, ensuring that the transformations are smooth and physically plausible. The final loss function is expressed as:

$$L_{total} = \sum_{i=-k, i\neq 0}^{k} w_i \|\hat{I}_m - I_{m+i}\|_2^2 + \lambda \sum_{t=1}^{T} \|\nabla \varphi_{m,f,t}\|_2^2, \tag{3}$$

where $k$ represents the number of neighboring slices and $w_i$ represents the weight between $\hat{I}_m$ and $I_{m+i}$, which is inversely proportional to the distance. T is the total number of iterations.

### S3.2 3D segmentation neural network

In the elastic registration experiment, we evaluate its usability in downstream tasks by performing 3D segmentation on the registration results. We apply state-of-the-art image segmentation model[15] to segment the registration results and the architecture of the segmentation network is shown in Figure S39. Our downstream experiment is designed to evaluate the impact of alignment quality on segmentation consistency rather than to benchmark the segmentation model itself. For this reason, we intentionally adopt a slice-wise 2D segmentation model. This design isolates the influence of alignment: if a simple 2D model already shows improved continuity and accuracy after alignment, such improvements can be attributed directly to the alignment method rather than to the implicit smoothing or volumetric regularization provided by a 3D network. Using a 3D segmentation model would obscure this effect, as its architecture can implicitly compensate for misalignment.

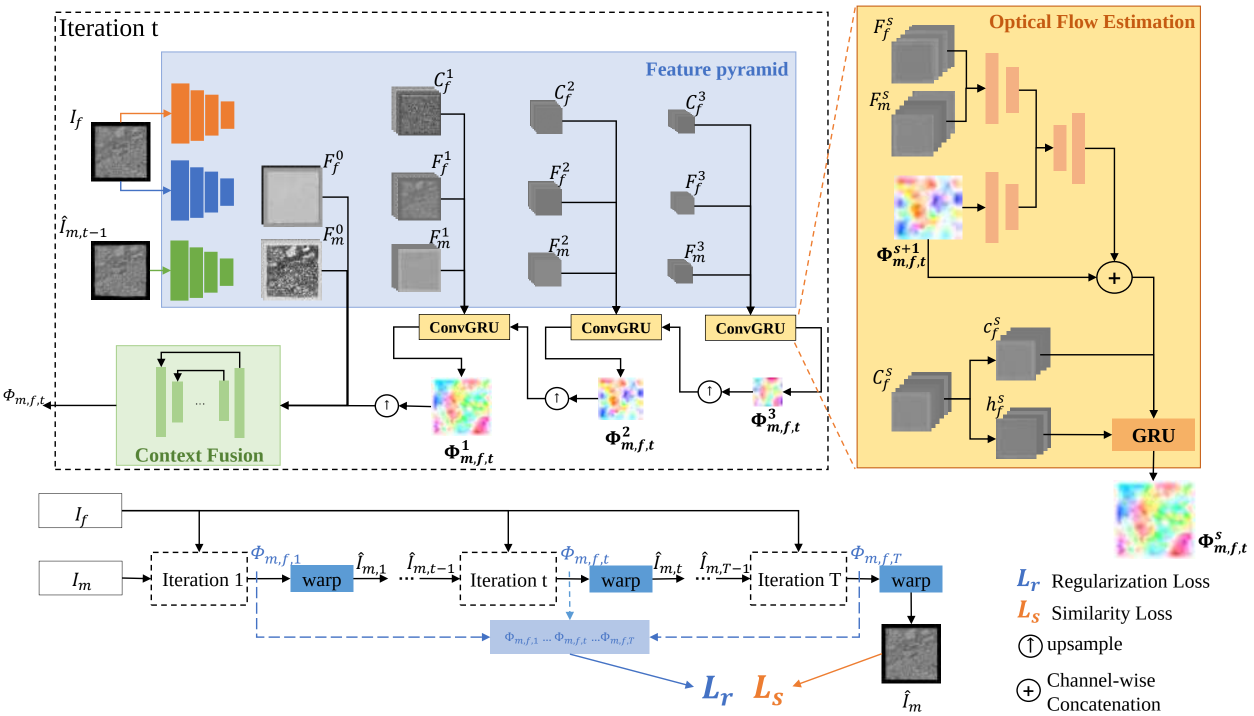


Figure S38: The network structure of the cascade optical flow estimation network.

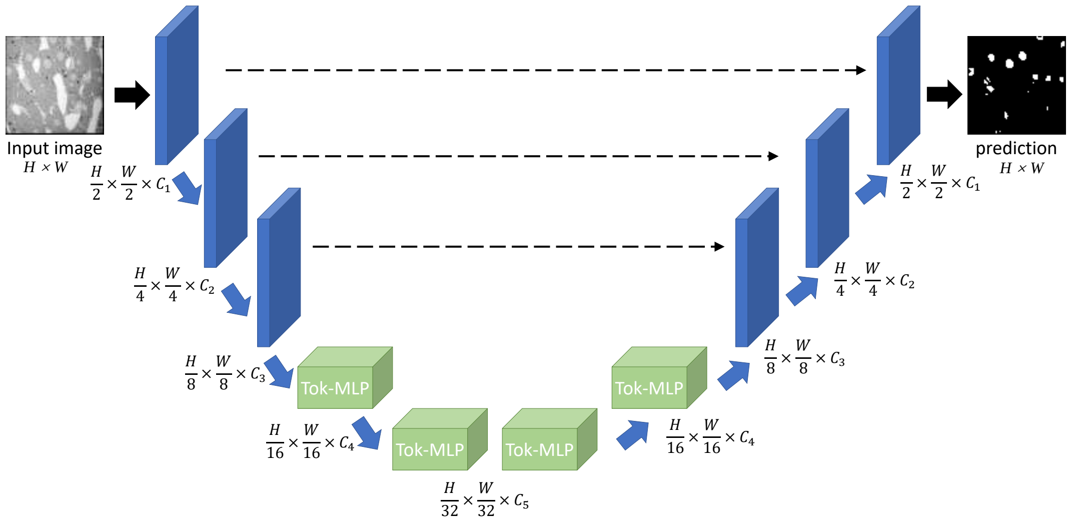


Figure S39: The network structure of the segmentation model used in 3D segmentation.

## S4 Experimental setup

**Rigid alignment baseline implementation details.** TrakEM2[10] software can be used as a plugin in FIJI (https://imagej.net/plugins/trakem2). Here, we set the default parameters as follows:

```
run("Linear Stack Alignment with SIFT",
"initial_gaussian_blur=1.60 steps_per_scale_octave=3 minimum_image_size=64
maximum_image_size=1024 feature_descriptor_size=4
feature_descriptor_orientation_bins=8 closest/next_closest_ratio=0.92
maximal_alignment_error=25 inlier_ratio=0.05
expected_transformation=Rigid interpolate");
```

StackReg [14] software can also be used as a plugin in FIJI (https://imagej.net/plugins/stackreg). Here, we perform alignment using its default parameters and select the transformation mode as "Rigid Body".

**Elastic registration baseline implementation details.** TrakEM2[11] software is available as a FIJI plugin (https://imagej.net/plugins/elastic-alignment-and-montage). We configured its hyperparameters as follows:

```
run("Elastic Stack Alignment",
"elastically=input_path resolution=128 scale=0.25
search_radius=50 block_radius=75 resolution=30 minimal_pmcc_r=0.6
maximal_curvature_ratio=10 maximal_second_best_r/best_r=0.9
use_local_smoothness_filter approximate_local_transformation=Rigid
local_region_sigma=150 maximal_local_displacement=20
maximal_local_displacement_0=3 series_is_aligned test_maximally=1
approximate_transformation=Rigid maximal_iterations=1000
maximal_plateauwidth=200 stiffness=0.10 maximal_stretch=2000
maximal_iterations_0=1000 maximal_plateauwidth_0=200");
```

SEAMLeSS[8] software is available on GitHub (https://github.com/seung-lab/corgie). Following the instructions in its documentation, we first applied the `corgie normalize` function to normalize the input 3D volume

blocks. Subsequently, we used the `corgie align-block` function to perform elastic registration on the normalized 3D volume blocks. The model used for registration can be found at gs://corgie_package/models/aligners/MICrONS_aligner_512_1024nm.

**vEMRec rigid alignment implementation details.** The rigid alignment module of vEMRec utilizes the feature point detection algorithm SuperPoint[3], which can be accessed on GitHub (https://github.com/rpautrat/SuperPoint). We directly employed the provided pre-trained model to extract feature points without performing fine-tuning on other datasets, with all hyperparameters set to their default values. For edge detection, we used the edge detection algorithm Rindnet[9] available on GitHub (https://github.com/MengyangPu/RINDNet). Similarly, we utilized the pre-trained model provided for edge detection without any fine-tuning.

**vEMRec elastic registration implementation details.** The elastic registration module of vEMRec employs a cascade optical flow estimation network to obtain the deformation fields between slices, and innovatively uses a Gaussian filter to integrate the resulting deformation fields. For the Gaussian filter parameters, we set the receptive field radius to $r = 1$, the maximum registration distance to $L = 1$, and the standard deviation of the Gaussian distribution to $\sigma = 3.0$. For the optical flow estimation network illustrated in Figure S38, we pre-trained the model using the Cremi dataset. The batch size was set to 4, with a learning rate of $5 \times 10^{-3}$, and the Adam optimizer was employed. The weight of the regularization term in the loss function, $\lambda$, was set to 18.0, and the model was trained for 100 epochs. Subsequently, we fine-tuned the model on the datasets provided by OpenOrganelle, again using a batch size of 4, a learning rate of $5 \times 10^{-3}$, and the Adam optimizer, with the same regularization weight $\lambda = 18.0$ for another 100 epochs.

To clarify the fairness of the comparison, it is important to outline the training and evaluation settings used for both methods. SEAMLeSS is pretrained on the MICrONS dataset, which is a neuronal dataset. We did not retrain SEAMLeSS on CREMI, as this would require retraining a third-party method beyond its intended usage and scope. Instead, we follow common practice in the field by using the officially released pretrained models for all methods and evaluating their cross-dataset generalization performance. Specifically, CREMI is a Drosophila brain neuronal dataset, and the CREMI-pretrained version of vEMRec is evaluated on multiple neuronal datasets, including FAFB (Drosophila brain) and mouse cortex, which represent diverse neuronal imaging conditions. Similarly, SEAMLeSS is pretrained on MICrONS, a mouse brain neuronal dataset, and is also evaluated on FAFB and mouse cortex in our benchmarks. In both cases, the pretrained models are applied to heterogeneous neuronal datasets without any

dataset-specific fine-tuning. Therefore, the comparison between vEMRec and SEAMLeSS reflects their cross-dataset generalization ability and is fair under this evaluation setting.

**3D segmentation network implementation details.** Regarding the segmentation network shown in Figure S39, we train a separate segmentation model for each test dataset. Specifically, for the training of each data type, we set the batch size to 4, the learning rate to $1 \times 10^{-4}$, and employed the Adam optimizer. All other hyperparameters were kept at their default settings, and the model was trained for a total of 500 epochs.

Table S11: Summary of experiments, datasets, and corresponding figures.

| Experiments | Datasets | Figures and Tables |
|---|---|---|
| 3D Rigid Alignment: Error Accumulation, High-Level Deformations, and Noise | Six mouse tissues including heart, kidney, liver, skin, and pancreas | Figure 2; Figures S5–S10; Table S4 |
| 3D Elastic Registration: Error Accumulation, High-Level Deformations, and Noise | Six mouse tissues including heart, kidney, liver, skin, and pancreas | Figure 2; Figures S11–S18; Table S6 |
| 3D Elastic Registration: Organellar 3D Segmentation | Six mouse tissues including heart, kidney, liver, skin, and pancreas | Figure 3; Figures S15, S16, S19, S20 |
| vEMRec on Real Datasets: Validation | FAFB (Full Adult Fly Brain, ssTEM/TEMCA); Caenorhabditis elegans cell; mouse cortical; female fruit fly brain neural; human term placental villi | Figure 5; Figures S21–S37 |
| vEMRec on Real Datasets: Downstream Isotropic Reconstruction Tasks | Female fruit fly brain neural; human term placental villi | Figure 6; Figures S26–S34 |
| vEMRec on Real Datasets: Downstream Neuron 3D Segmentation Tasks | Female fruit fly brain neural; mouse cortical | Figure 6; Figure S35 |

# References


[1] (2025). Functional connectomics spanning multiple areas of mouse visual cortex. *Nature*, **640**(8058), 435–447.

[2] Bumbarger, D. J., Riebesell, M., Rödelsperger, C., and Sommer, R. J. (2013). System-wide rewiring underlies behavioral differences in predatory and bacterial-feeding nematodes. *Cell*, **152**(1), 109–119.

[3] DeTone, D., Malisiewicz, T., and Rabinovich, A. (2018). Superpoint: Self-supervised interest point detection and description. In *Proceedings of the IEEE conference on computer vision and pattern recognition workshops*, pages 224–236.

[4] Heinrich, L., Bennett, D., Ackerman, D., Park, W., Bogovic, J., Eckstein, N., Petruncio, A., Clements, J., Pang, S., Xu, C. S., *et al.* (2021). Whole-cell organelle segmentation in volume electron microscopy. *Nature*, **599**(7883), 141–146.

[5] Jia, X., Bartlett, J., Zhang, T., Lu, W., Qiu, Z., and Duan, J. (2022). U-net vs transformer: Is u-net outdated in medical image registration? In *International Workshop on Machine Learning in Medical Imaging*, pages 151–160. Springer.

[6] Kasthuri, N., Hayworth, K. J., Berger, D. R., Schalek, R. L., Conchello, J. A., Knowles-Barley, S., Lee, D., Vázquez-Reina, A., Kaynig, V., Jones, T. R., *et al.* (2015). Saturated reconstruction of a volume of neocortex. *Cell*, **162**(3), 648–661.

[7] Lewis, R. M., Baskaran, H., Green, J., Tashev, S., Palaiologou, E., Lofthouse, E. M., Cleal, J. K., Page, A., Chatelet, D. S., Goggin, P., *et al.* (2022). 3d visualization of trans-syncytial nanopores provides a pathway for paracellular diffusion across the human placental syncytiotrophoblast. *Iscience*, **25**(12).

[8] Popovych, S., Macrina, T., Kemnitz, N., Castro, M., Nehoran, B., Jia, Z., Bae, J. A., Mitchell, E., Mu, S., Trautman, E. T., *et al.* (2024). Petascale pipeline for precise alignment of images from serial section electron microscopy. *Nature Communications*, **15**(1), 289.

[9] Pu, M., Huang, Y., Guan, Q., and Ling, H. (2021). Rindnet: Edge detection for discontinuity in reflectance, illumination, normal and depth. In *Proceedings of the IEEE/CVF international conference on computer vision*, pages 6879–6888.

[10] Saalfeld, S., Cardona, A., Hartenstein, V., and Tomančák, P. (2010). As-rigid-as-possible mosaicking and serial section registration of large sstem datasets. *Bioinformatics*, **26**(12), i57–i63.

[11] Saalfeld, S., Fetter, R., Cardona, A., and Tomancak, P. (2012). Elastic volume reconstruction from series of ultra-thin microscopy sections. *Nature methods*, **9**(7), 717–720.

[12] Simard, P. Y., Steinkraus, D., Platt, J. C., *et al.* (2003). Best practices for convolutional neural networks applied to visual document analysis. In *Icdar*, volume 3. Edinburgh.

[13] Takemura, S.-y., Bharioke, A., Lu, Z., Nern, A., Vitaladevuni, S., Rivlin, P. K., Katz, W. T., Olbris, D. J., Plaza, S. M., Winston, P., *et al.* (2013). A visual motion detection circuit suggested by drosophila connectomics. *Nature*, **500**(7461), 175–181.

[14] Thevenaz, P., Ruttimann, U. E., and Unser, M. (1998). A pyramid approach to subpixel registration based on intensity. *IEEE transactions on image processing*, **7**(1), 27–41.

[15] Valanarasu, J. M. J. and Patel, V. M. (2022). Unext: Mlp-based rapid medical image segmentation network. In *International conference on medical image computing and computer-assisted intervention*, pages 23–33. Springer.

[16] Xu, C. S., Pang, S., Shtengel, G., Müller, A., Ritter, A. T., Hoffman, H. K., Takemura, S.-y., Lu, Z., Pasolli, H. A., Iyer, N., *et al.* (2021). An open-access volume electron microscopy atlas of whole cells and tissues. *Nature*, **599**(7883), 147–151.

[17] Zheng, Z., Lauritzen, J. S., Perlman, E., Robinson, C. G., Nichols, M., Milkie, D., Torrens, O., Price, J., Fisher, C. B., Sharifi, N., *et al.* (2018). A complete electron microscopy volume of the brain of adult drosophila melanogaster. *Cell*, **174**(3), 730–743.

Table S12: List of symbols and their definitions

| **Symbol** | **Definition** |
|---|---|
| $I_t$ | The $t$-th slice in the image stack |
| $\{S_i\}_{i=0}^N, \{T_i\}_{i=0}^N$ | Source keypoint set and target keypoint set |
| $\delta(i, j)$ | If 1, $(S_i, T_j)$ is a bidirectional match; if 0, it is not |
| $NN(\cdot)$ | The nearest neighbor function, returns a boolean value |
| $X, Y$ | Source keypoint set and target keypoint set after geometric consistency constraints |
| $U\Sigma V$ | The SVD matrix |
| $R_t$ | The rotation matrix for the rigid rotation of the $t$-th slice |
| $t_t$ | The translation vector for the rigid displacement of the $t$-th slice |
| $\bar{I}_t$ | The $t$-th slice after rigid transformation |
| $\varphi_i$ | The deformation field used to warp the $i$-th slice |
| $\varphi_{i,j}$ | The deformation field that registers slice $I_i$ to slice $I_j$ |
| $\varphi_{i,j,t}$ | Deformation field output from the optical flow estimation network at iteration $t$ |
| $\hat{I}_{m,t}$ | Warped image obtained by applying the deformation field $\varphi_{m,f,t}$ at iteration $t$ |
| $\{C_f^s\}_{s=1}^3$ | Contextual feature pyramids constructed in the optical flow estimation network |
| $\{F_f^s\}_{s=0}^3$ | Feature pyramid of the fixed image constructed in the optical flow estimation network |
| $\{F_m^s\}_{s=0}^3$ | Feature pyramid of the moving image constructed in the optical flow estimation network |
| $\{\varphi_{m,f,t}^s\}_{s=1}^3$ | Intermediate deformation fields in the $t$-th iteration of the optical flow estimation network |
| $r$ | The receptive field radius of the 1D Gaussian filter |
| $L$ | The maximum registration distance |
| $g_i$ | The weight at position $i$ in the 1D Gaussian filtering |
| $\varphi_{i,j}^{tmp}$ | The intermediate deformation field generated in the Gaussian filtering strategy |
| $I_i^{tmp}$ | The intermediate slice generated from slice $I_i$ in the Gaussian filtering strategy |
| $g_i^{tmp}$ | The intermediate weight generated from the Gaussian weight $w_i$ in the Gaussian filtering strategy |
| $\bar{g}_i^{tmp}$ | The normalized intermediate weight |
| $D$ | The difference image |
| $\nabla^2 D$ | The Laplacian-filtered image |
| $H$ | The image entropy |
| $SNR$ | The signal-to-noise ratio |
| $\{DoG_i\}_{i=0}^3$ | Difference of Gaussian pyramid |
| $GLCM_i$ | The gray level co-occurrence matrix at the $i$-th layer |
| $Con_i$ | The contrast at the $i$-th layer |
