# Supplementary material for: vEMRec: High‐Resolution Volume Electron Microscopy Reconstruction Based on Structure‐Preserving and High‐Fidelity 3D Alignment
Source: Adv Sci (Weinh). 2026 Feb 20;13(23):e19098. doi: 10.1002/advs.202519098 (PMC13104135; doi:10.1002/advs.202519098)
Supplement: Supplementary file 2 — Supporting File 2: advs74367‐sup‐0002‐SuppMat.pdf. [file ADVS-13-e19098-s001.pdf]

vEMRec: High-Resolution Volume Electron Microscopy Reconstruction Based on Structure-Preserving and High-Fidelity 3D Alignment

Contents

S1 Overview **2**

S2 Installation **2**

S3 Walkthrough **3**

S3.1 3D rigid alignment 3

S3.2 3D Elastic Registration 3

S3.2.1 Testing 3

S3.2.2 Training 4

S4 Availability of data **5**

S1 Overview

Here, we introduce vEMRec, a feature-based 3D rigid alignment and Gaussian-filter-based 3D elastic registration method aimed at eliminating rigid misalignment and nonlinear distortion to restore the true 3D structure of biological specimens. The method has two key stages: sequential rigid alignment to correct rotation and displacement, followed by Gaussian filtering to address nonlinear distortions. During rigid alignment, stable edge features are extracted and matched to compute transformation parameters. In the elastic registration phase, a 1D Gaussian filter is applied to decouple nonlinear distortions from natural deformations. This approach effectively corrects distortions while preserving the integrity of the biological structure, providing a strong foundation for further analysis.

S2 Installation

- **Clone this repository**

```
https://drive.google.com/drive/folders/1fkLuQgJ5rGAd5RV2oY-gx6fdRXBNpnlh?usp=sharing
```

- **Create conda environment and activate**

```
conda create -n vEMRec python=3.9
conda activate vEMRec
```

- **Install dependencies**

```
pip install -r requirements.txt
```

S3 Walkthrough

S3.1 3D rigid alignment

The following explains how to perform edge detection and 3D rigid alignment on a series of PNG images using vEMRec. The images should be correctly numbered (e.g., 0000.png, 0001.png, etc.) in the source folder.

- **Edge detection**

```
cd src/rigid/edge
python evaluate_edge.py --input_path /path/to/img_folder --output_dir /path/to/mask_folder
```

Next, you can run the following code to perform 3D rigid alignment:

- **Alignment**

```
cd src/rigid
python main.py --iters 5 --input_dir /path/to/img_folder --input_mask /path/to/mask_folder --output_dir /path/to/output_folder --use_ransac 1
```

S3.2 3D Elastic Registration

In this section, this guide will explain how to use vEMRec for 3D elastic registration.

S3.2.1 Testing

vEMRec supports two forms of 3D elastic registration. For small-sized images (around 1024 pixels), you can run the following code to achieve 3D elastic registration.

- **small-sized**

```
cd src/elastic
python single_process.py --input_dir /path/to/img_folder --output_dir /path/to/output_folder --model_path /path/to/model
```

For large-sized images, you can run the following code to perform 3D elastic registration.

- **large-sized**

```
cd src/elastic
python process_big.py --input_dir /path/to/img_folder --output_dir /path/to/output_folder --model_path /path/to/model --height large_image_height --width large_image_width --patch_sz 1024 --overlap 50
```

S3.2.2 Training

vEMRec estimates the displacement field between slices using a optical flow neural network and integrates the displacement field with a Gaussian filter. Here, we outline the preparation of training data and the training for the network.

S3.2.2.1 Data preparation

The optical flow network in vEMRec is trained on the CREMI[1] and fine-tuned on the dataset provided by OpenOrganelle[2]. Download the training data on Cremi website. Then, run the following code:

```
cd src/utils
python aug_data.py --input_file /path/to/sample_A_padded_20160501.hdf --output_dir /path/to/train_data/a_padded --size 1024 --border 80
python deform_serial.py --input_file /path/to/train_data/a_padded --output_dir /path/to/train_data/a_padded_warp --alpha 4.0 --sigma
```

[1] https://cremi.org/
[2] https://openorganelle.janelia.org/

```
    0.08
```

For the data provided by OpenOrganelle, run the following code to generate the training dataset.

```
cd src/utils
sh download.sh
```

S3.2.2.2 Train

Run the following code to train the model:

```
cd src/elastic
python train.py --dataset cremi --root_dataset /path/to/train_data --
    base_path /path/to/result
```

S4 Availability of data

The complete experimental data for vEMRec is stored in Aliyun Cloud. You can use ossutil (`https://github.com/aliyun/ossutil`) to access our data. You can download the data using the access account we provide.

```
accessKeyID=LTAI5tPQCgcrv2kPjLGqZN2d
accessKeySecret=IQCaXWo0NEHL5c0gC0BELpqxkLnneb
```
